# Efficacy and Safety of Biologic Therapies Targeting Type 2 and Epithelial-Alarmin Pathways in Chronic Obstructive Pulmonary Disease: A Systematic Review and Network Meta-Analysis of Randomized Controlled Trials

**DOI:** 10.7759/cureus.115334

**Published:** 2026-08-28

**Authors:** Yasser A Khoshaim, Ahmad R Almaghrabi, Ahmed A Alruwaythi, Abdulkhaliq E Alotaibi, Ibrahim M Alhoshan, Talal A Almalki, Abdullah A Sabbah, Abdullah W Al-Shuaibi, Osama N Almarzoqi, OBai T Mesawa, Alwaleed Alabdali

**Affiliations:** 1 Department of Medicine, King Abdullah Medical Complex, Jeddah, SAU; 2 Faculty of Medicine, King Abdulaziz University, Rabigh, SAU; 3 Faculty of Medicine, King Abdulaziz University, Jeddah, SAU; 4 Department of Home Health Care, Dr. Soliman Fakeeh Hospital, Jeddah, SAU; 5 Department of Clinical Sciences, Fakeeh College for Medical Sciences, Jeddah, SAU

**Keywords:** biologics, copd, dupilumab, eosinophils, exacerbations, grade, network meta-analysis

## Abstract

People with chronic obstructive pulmonary disease (COPD) who have type 2/eosinophilic inflammation may remain at high exacerbation risk despite optimized triple inhaled therapy. Seven monoclonal antibodies targeting four pathways, namely, interleukin (IL)-4/13, IL-5/IL-5 receptor α (IL-5Rα), IL-33/suppression of tumorigenicity 2 (ST2), and thymic stromal lymphopoietin (TSLP), have now been tested in COPD, and previous COPD-biologics network meta-analyses provided cross-agent rankings and certainty assessments but predated the 2025-26 pivotal and alarmin-trial wave and did not include the complete seven-agent, three-pathway evidence set. We aimed to compare and rank biologics targeting type 2 and epithelial-alarmin pathways for moderate/severe COPD exacerbations. We conducted a systematic review and random-effects network meta-analysis of placebo-controlled randomized controlled trials (RCTs) of eligible biologics in adults with COPD, prospectively registered in the International Prospective Register of Systematic Reviews (PROSPERO; CRD420261448694) and reported according to the PRISMA 2020 statement and its extension for network meta-analysis (PRISMA-NMA). MEDLINE via PubMed, CENTRAL, Europe PMC, ClinicalTrials.gov, the WHO International Clinical Trials Registry Platform (WHO ICTRP), and Google Scholar were searched to 12 July 2026. Two independent reviewers screened records, with disagreements resolved by adjudication and quality control. Risk of bias was assessed using Cochrane Risk of Bias 2 (RoB 2). The primary outcome was the annualized rate of moderate/severe exacerbations, expressed as a rate ratio (RR) with 95% confidence interval (CI) versus placebo and analysed with frequentist netmeta (placebo-anchored, star network), with Bayesian sensitivity analyses and P-score ranking; certainty was assessed using Grading of Recommendations Assessment, Development and Evaluation (GRADE) via Confidence in Network Meta-Analysis (CINeMA). Of 2,719 records identified and 2,321 screened, 15 trial nodes were included; the primary maintenance exacerbation network comprised nine RCTs (placebo plus four agents: dupilumab, mepolizumab, benralizumab, astegolimab). All four agents reduced moderate/severe exacerbations versus placebo, three significantly. Dupilumab had the largest estimated relative reduction and highest P-score (RR: 0.68, 95% CI: 0.59-0.79; P-score: 0.9947), followed by astegolimab (RR: 0.85, 95% CI: 0.75-0.96; P-score: 0.5766), mepolizumab (RR: 0.88, 95% CI: 0.79-0.97; P-score: 0.4708) and benralizumab (RR: 0.88, 95% CI: 0.77-1.01; P-score: 0.4471). Heterogeneity was low (I² = 9.68%). Dupilumab was favored over each other agent in indirect comparisons via the common placebo; the astegolimab/mepolizumab/benralizumab cluster did not differ significantly. Dupilumab remained first across all sensitivity analyses and all three analytic engines, but the complete ranking was not stable. GRADE-CINeMA certainty was moderate for dupilumab and low for the other three agents. Across completed COPD biologic RCTs, dupilumab (in an eosinophil-enriched population) is the most effective agent for reducing moderate/severe exacerbations; astegolimab, mepolizumab, and benralizumab produce smaller, similar reductions of uncertain relative order. The evidence is limited by a star-shaped network (no head-to-head trials; transitivity untestable) and by mixing of eosinophil populations across trials.

## Introduction and background

Chronic obstructive pulmonary disease (COPD) is a leading cause of morbidity and mortality worldwide [1,2], and 20-40% of patients have type 2/eosinophilic inflammation [3,4] associated with continued exacerbations despite optimized triple inhaled therapy with an inhaled corticosteroid (ICS), a long-acting β2-agonist (LABA), and a long-acting muscarinic antagonist (LAMA) [4,5]. Exacerbations drive lung-function decline, hospitalization, and death [6,7], and a residual, biologically targetable inflammatory burden has motivated trials of monoclonal antibodies directed at type 2 cytokines and their upstream epithelial alarmins.

Seven biologics across three pathways have now been tested in COPD randomized controlled trials (RCTs): anti-interleukin (IL)-4 receptor α (IL-4Rα; dupilumab), which blocks the receptor subunit shared by IL-4 and IL-13 signaling [8]; anti-IL-5/IL-5 receptor α (IL-5Rα; mepolizumab, benralizumab), which inhibit or deplete the eosinophil-maturation and -survival cytokine IL-5 [9]; and epithelial-alarmin agents targeting IL-33/suppression of tumorigenicity 2 (ST2) and thymic stromal lymphopoietin (TSLP), itepekimab, tozorakimab, astegolimab, and tezepelumab, cytokines released by airway epithelium upstream of both type 2-high and type 2-low inflammation [10]. Blood eosinophil count remains the principal biomarker used to guide ICS and, increasingly, biologic treatment escalation in COPD [11,12]. Dupilumab and mepolizumab have entered guidelines [5,13], and dupilumab became the first biologic to receive regulatory approval for COPD in 2024 [14]; the alarmin agents reported pivotal or phase 2 results in 2025-2026 with mixed, largely eosinophil-independent effects, a pattern that mirrors the eosinophil-independent efficacy already established for TSLP blockade in severe asthma [15].

Pitre et al. synthesized COPD-biologics trials through July 2024 using frequentist network meta-analysis and the Grading of Recommendations Assessment, Development and Evaluation (GRADE) approach [16]. Subsequent publication of MATINEE [17], ALIENTO/ARNASA [18], and FRONTIER-4 [19] materially expanded the evidence base. We therefore updated the network through July 2026 across the complete seven-agent, three-pathway scope and explicitly assessed eosinophil-population and pathway-related transitivity.

Objective

To estimate and rank the comparative efficacy of type 2- and epithelial-alarmin-targeting biologics for reducing moderate/severe exacerbations and improving lung function and health status in adults with COPD and to summarize safety outcomes narratively.

## Review

Materials and methods

Protocol and Registration

The review was prospectively registered (PROSPERO CRD420261448694, published 2026-07-12, before the formal search and screening) and follows PRISMA 2020 [20], the PRISMA-NMA extension [21], and PRISMA-S [22] for search reporting. The protocol (v0.3) was finalized on 2026-07-11 and is deposited, together with the extraction workbook, the analysis code, and the complete audit trail, in a public repository. Every protocol deviation is logged and disclosed in "Protocol deviations".

Eligibility Criteria (PICO)

The following are the eligibility criteria: (1 )Population, adults (≥18 years) with spirometry-confirmed COPD (post-bronchodilator FEV₁/FVC <0.70) on maintenance inhaled therapy - eosinophil phenotype recorded, not required; (2) Interventions (network nodes), dupilumab, mepolizumab, benralizumab, itepekimab, tozorakimab, astegolimab, tezepelumab, any dose/schedule, added to standard care; (3) Comparator, placebo plus standard care; (4) Outcomes, primary - annualized rate of moderate/severe exacerbations (rate ratio); secondary efficacy - change in pre-/post-bronchodilator FEV₁, SGRQ, E-RS: COPD, time to first exacerbation, rescue-medication use; and safety - TEAEs, SAEs, discontinuations for AEs, all-cause death, and AEs of special interest. This also includes the following: design, randomized, placebo-controlled, parallel-group trials. Maintenance add-on trials in stable COPD form the primary network; trials initiating a biologic at/after hospitalization for an acute exacerbation were analysed separately (rubric I6). The operational inclusion/exclusion rubric (codes I1-I7/E1-E7), with worked application notes, was deposited online (link in the Appendix).

Information Sources

Because institutional access to Embase, Scopus, and Web of Science was unavailable (deviation 2), we searched free-access sources only: PubMed/MEDLINE, Cochrane CENTRAL, Europe PMC, and Google Scholar (supplement), plus the ClinicalTrials.gov and WHO ICTRP registries, to 2026-07-12. OpenAlex/Crossref/Semantic Scholar/scite supported citation chasing and deduplication; ATS/ERS proceedings were checked for grey literature; manufacturer results records were checked for outcome completeness, without language or date restriction. Full strategies and each tool's role are given in the Appendix (PRISMA-S).

Selection Process

Two independent reviewers screened every title and abstract on 12 July 2026, resolving the 388 disagreement/code rows by adjudication, with review of a 214-record agreement quality-control sample and a risk-based check of borderline decisions. Full-text eligibility was assessed independently in duplicate by the two reviewers against the completeness and E6 criteria, and the two count-changing disagreements were resolved by discussion before the final included set was agreed. Multiple reports of one trial were linked to a single node to prevent double-counting.

Data Collection and Items

Data were extracted into a standardized workbook covering trial identifiers, agent/pathway, design, country/sites, N per arm, population (baseline blood eosinophils and threshold, background therapy, global initiative for chronic obstructive lung disease (GOLD) stage/forced expiratory volume in one second (FEV₁), exacerbation history, smoking), dose/schedule, duration, funding/sponsor and conflict-of-interest information, all outcomes with effect estimates and variances, per-arm safety counts, and eosinophil-subgroup estimates where reported. All results compatible with the prespecified outcome domains that were available in eligible sources were sought across eligible doses and reported time points. The primary synthesis used each trial's prespecified primary maintenance endpoint and the pivotal/primary-analysis dose according to the pool map; non-pivotal arms were retained for narrative or sensitivity analysis. Missing or unclear values were not imputed or treated as zero; they were retained as not reported/not obtainable, and source conflicts and computed variances were logged explicitly. Data were extracted independently in duplicate by two reviewers and reconciled by discussion; the workbook was subsequently reopened and corrected against fresh primary evidence during a data-verification audit (see "Protocol deviations"). Every pooled value traced to a verified extraction entry; the finalized workbook was completed on 2026-07-15.

Risk of Bias

Risk of bias was assessed with the Cochrane RoB 2 tool [23] for parallel-group trials (effect of assignment to intervention), across five domains plus an overall judgment, for the primary exacerbation result, and visualised with robvis [24]. Two reviewers assessed every trial independently and in duplicate. Inter-rater agreement between the two independent assessments, before any adjudication, was unweighted Cohen's κ 0.464 across 90 paired judgments (raw agreement: 78.9%); κ was 0.463 across the 75 domain-level judgments and 0.405 across the 15 overall judgments.

Every judgment was then adjudicated by the guarantor against the source evidence, including trial protocols, statistical analysis plans, registry participant flow, and posted results records. Adjudication was evidence-based rather than a reconciliation of votes: in 17 of the 90 judgments the adjudicated result differed from both independent assessments, in most cases because a statistical analysis plan was found to be dated after primary completion, which was recorded as a domain 5 concern that neither initial assessment had captured. The adjudicated judgments, each with its supporting evidence, and the resulting overall ratings were signed off by the guarantor. Assessor-level judgments, the adjudication record with per-row supporting evidence, and the agreement calculation are provided in the data deposit so that every judgment can be traced to its source.

Risk-of-bias judgments inform the certainty assessment only; they do not enter the network estimates, so no adjudication decision can affect any reported rate ratio or the ranking.

Effect Measures

Exacerbations were analysed as a rate ratio (log-RR, generic inverse variance from adjusted annualized-rate intervals; direct adjusted 95% CIs from the raw registry results for GALATHEA and TERRANOVA [25]; the prespecified two-sided 90% CI for COURSE [26]). Standard errors were calculated as

*(ln(upper) &minus; ln(lower)) / (2z)*

using z = 1.959964 for 95% intervals and the corresponding 90% quantile for COURSE. Continuous outcomes were analysed as mean difference, and binary safety as risk/odds ratio.

Synthesis and Network Meta-Analysis

Nine maintenance RCTs formed the primary network; four phase-2/2a trials with compatible rate ratios entered the sensitivity layer; COPD-HELP was analysed separately [27]; FRONTIER-4 remained unconnected because it reported a hazard ratio rather than a rate ratio. We fit a random-effects, placebo-anchored network meta-analysis using generic inverse variance and the frequentist graph-theoretical method [28] (netmeta 3.3.1 [29], R 4.4.2), with a common DerSimonian-Laird heterogeneity estimate. We reported each agent's network RR versus placebo, all pairwise league estimates, 95% prediction intervals, a forest plot, network graph, and P-scores [30]. One node per agent was used at its pivotal/primary-analysis dose; non-pivotal arms were retained for narrative reporting or the astegolimab schedule sensitivity. Sensitivities added the phase 2 layer, restricted to phase 3 trials, used a common-effect model, removed one agent at a time, ran protocol-specified multinma [31], added a gemtc [32] cross-check, and applied the post-protocol astegolimab schedule analysis. The planned acute pooled sensitivity was not possible because only one acute-initiation trial remained; eosinophil meta-regression was attempted but rank-deficient. A pre-specified sanity check required dupilumab versus placebo ≈0.68.

Transitivity, Subgroups, and Reporting Bias

Because no head-to-head biologic trials exist, the network is star-shaped with no closed loops; statistical inconsistency (node-splitting, design-by-treatment) is not estimable, and validity rests on the transitivity assumption, assessed clinically across baseline eosinophils,exacerbation history, background therapy, severity, and trial duration. Pre-specified subgroup/meta-regression analyses were eosinophil band (<150/150-<300/≥300 cells/µL), pathway class, duration and phase; eosinophil-stratified networks were the primary novel analysis. Reporting bias was to be assessed by comparison-adjusted funnel plot only where a contrast had ≥10 studies; otherwise, narratively with registry-vs-publication outcome checking.

Certainty

Certainty of network estimates used GRADE for NMA via CINeMA [33] (within-study bias, reporting bias, indirectness, imprecision, heterogeneity, incoherence), summarized in the "Summary of Findings" table. An assessment-time range of equivalence (RR: 0.80-1.25) was used for imprecision; this was not protocol-pre-specified and is disclosed as deviation 5. It was accepted on 2026-07-15, and the letter grades are unchanged under a null-only threshold. The CINeMA six-domain framework was applied to the current remediated network outputs and RoB 2 judgments; the native web application can optionally reproduce the report from the prepared input file if required by the journal.

Protocol Deviations and Post-lock Amendments (Disclosed)

(1) The scope was broadened on 2026-07-11 from a dupilumab-only pairwise meta-analysis to a biologics-class network meta-analysis (retitled; a replacement PROSPERO record was registered). (2) The search was restricted to free-access sources because institutional access to Embase, Scopus, and Web of Science was unavailable. (3) During a data-verification audit, it was found that the ClinicalTrials.gov query used during data extraction had omitted data posted in the Results section, resulting in genuine MATINEE secondary and safety values being misclassified as unavailable; a direct registry (REST v2) retrieval of all 15 trials corrected those classifications before synthesis, and no pooled primary point estimate changed. An erroneous author attribution for MATINEE was also identified and corrected. (4) A subsequent audit found that the posted adjusted direct 95% treatment-comparison CIs for GALATHEA and TERRANOVA should replace the variance reconstructed from marginal arm CIs; the analysis and reporting were rerun, and point estimates, rank order and substantive conclusions were unchanged while exact network CIs, common heterogeneity and P-scores shifted slightly (remediated package finalized 2026-07-15). (5) The CINeMA imprecision range of equivalence of RR 0.80-1.25 was introduced at the certainty-assessment stage rather than pre-specified; it is disclosed as a deviation and was accepted on 2026-07-15. In addition, a WHO ICTRP row-level export gap removed 33 records before screening, and five pivotal phase3 programs (RESOLUTE, AERIFY-1/2, TITANIA, OBERON) had press-release toplines but no peer-reviewed or extractable results by the search date (excluded E6). Secondary and safety networks were not poolable and are reported narratively.

Results

Study Selection

**Figure 1:**
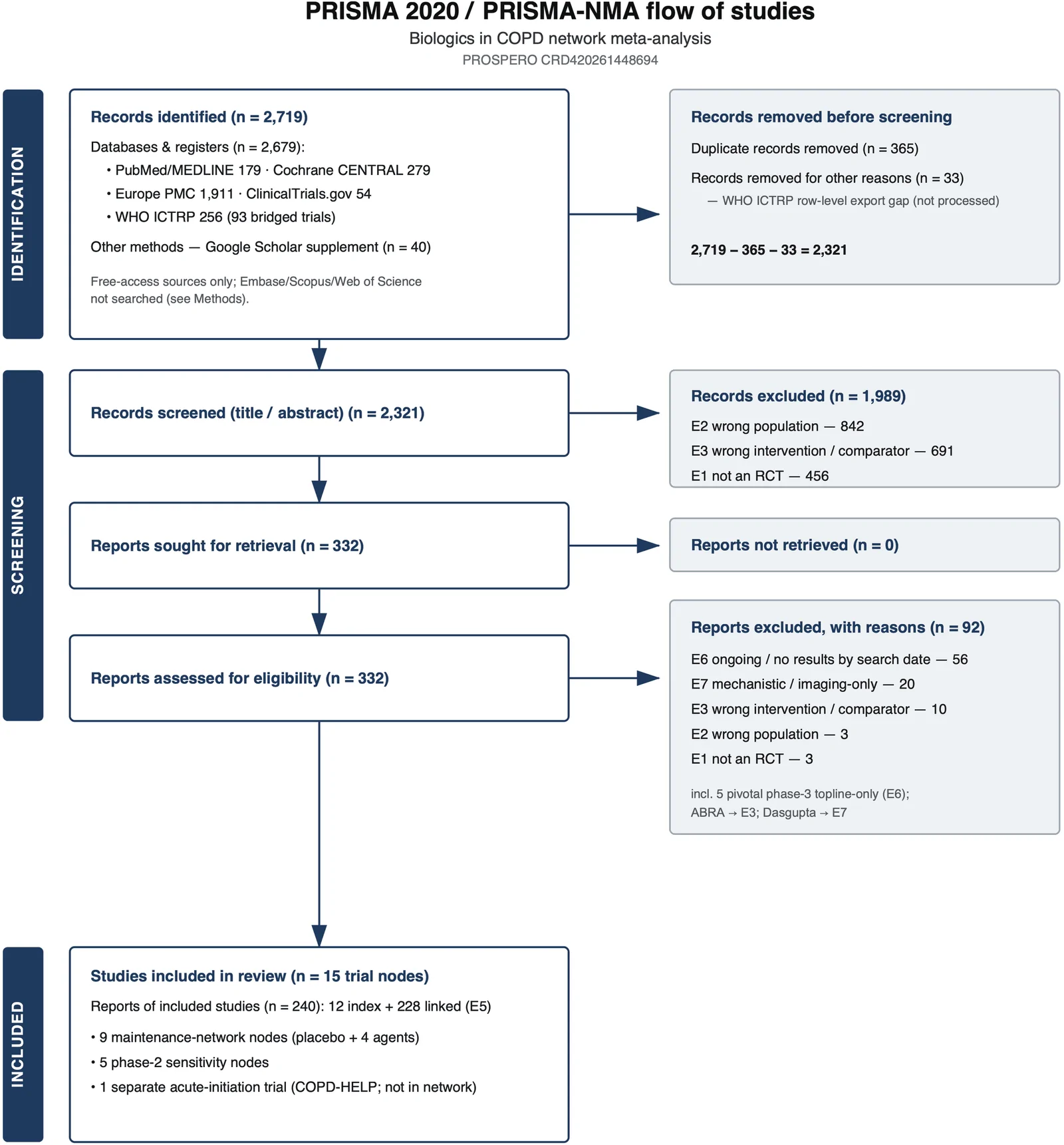
PRISMA 2020/PRISMA-NMA study selection flow Searches identified 2,719 records. After removal of 365 duplicates and 33 records affected by the WHO International Clinical Trials Registry Platform (ICTRP) row-level export gap, 2,321 records underwent dual title/abstract screening; 332 reports were assessed, 92 were excluded with reasons, and 240 reports were linked to 15 unique trials. The included set comprised nine maintenance trials forming the primary network (k = 9), four phase-2/2a trials entering the sensitivity layer only, one unconnected trial reporting a hazard ratio rather than a rate ratio (FRONTIER-4), and one separately analysed acute-initiation trial (COPD-HELP).

The search identified 2,719 records (databases/registers 2,679 plus Google Scholar supplement 40). After removing 365 duplicates and 33 records via the ICTRP export gap, 2,321 records were screened; 1,989 were excluded (E2 wrong population 842, E3 wrong intervention/comparator 691, E1 not an RCT 456) and 332 reports were sought and assessed (0 not retrieved). Ninety-two reports were excluded with reasons (E6 ongoing/no results 56, E7 mechanistic/imaging-only 20, E3 10, E2 3, E1 3), leaving 240 reports of 15 included trials (12 index publications; three papers each report a paired program) (Figure 1). Adjudicated exclusions included the Dasgupta mepolizumab pilot (NCT01463644, E7) and benralizumab ABRA (NCT04098718, E3; active comparator, mixed population).

Study and Network Characteristics

**Figure 2:**
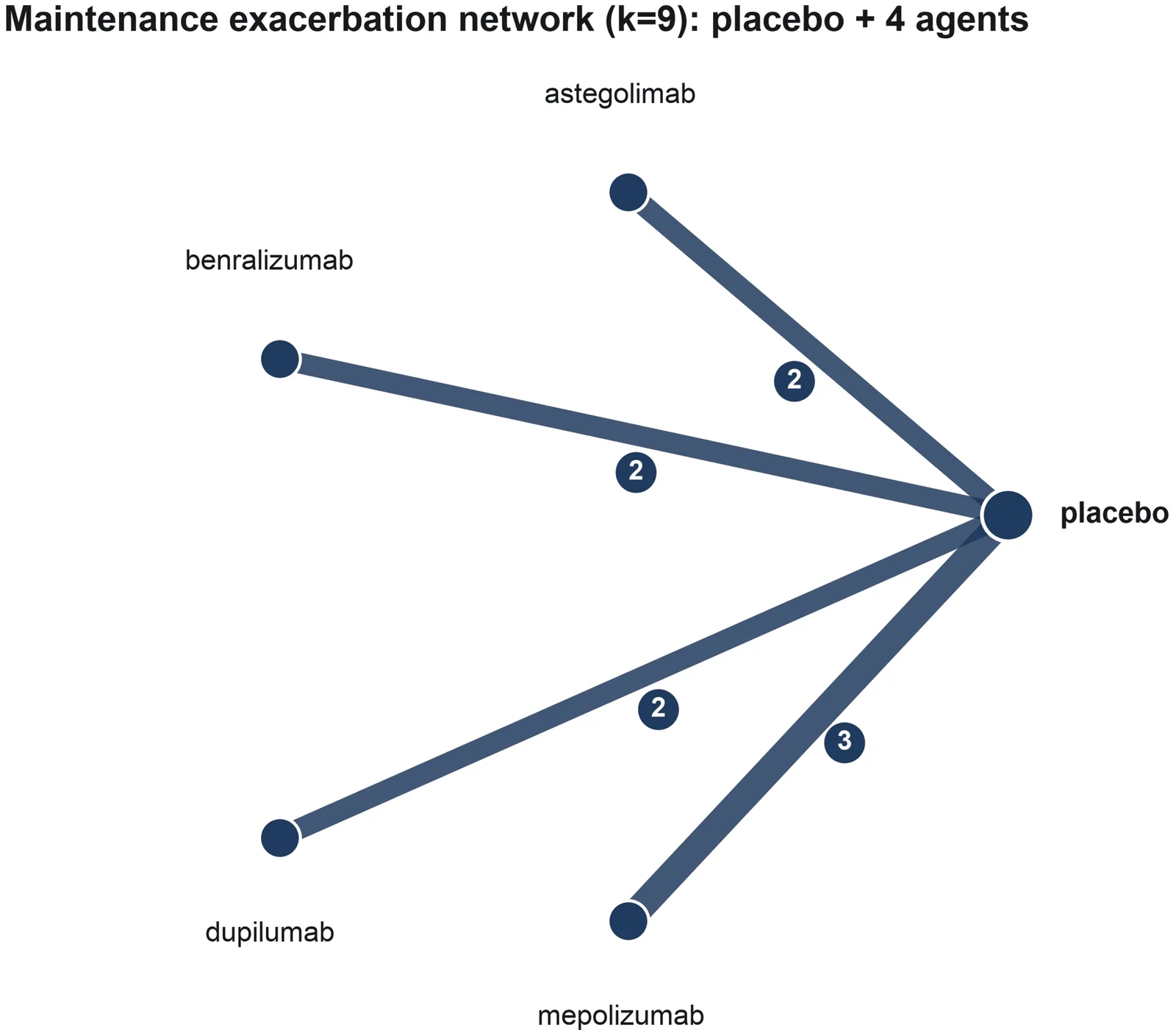
Geometry of the primary maintenance exacerbation network The network has five vertices, placebo and the four connected biologics, and is fitted on nine maintenance trials (k = 9). Edge width and the printed edge number give the number of direct placebo-controlled trials on that edge: dupilumab two, astegolimab two, mepolizumab three, and benralizumab two, summing to the nine trials of the primary network. The remaining six of the 15 included trials do not appear here: four enter the sensitivity layer only, FRONTIER-4 is unconnected, and COPD-HELP is analysed separately. No active-agent edges or closed loops exist; all active-active estimates are indirect through placebo.

Fifteen trials met the inclusion criteria, and they contribute to the analysis in four distinct ways, recorded for every trial in the Contributes to column of Table 1. Nine maintenance trials form the primary network and are the k = 9 fitted in Table 2: BOREAS [34] and NOTUS [35] (dupilumab); METREX, METREO [36] and MATINEE [17] (mepolizumab); GALATHEA and TERRANOVA [25] (benralizumab); and ALIENTO and ARNASA [18] (astegolimab). Four phase-2/2a trials report a commensurable rate ratio but no maintenance-network edge and enter the sensitivity layer only: the itepekimab proof-of-concept trial [37], COPD-ST2OP [38], COURSE [26], and the benralizumab phase-2a trial [39]. FRONTIER-4 (tozorakimab) [19] reports a COPDCompEx time-to-first-event hazard ratio rather than an annualized rate ratio and is therefore not connected to any network. COPD-HELP initiates mepolizumab at hospitalization for an acute exacerbation rather than in stable maintenance and is analysed separately [27]. This 9 + 4 + 1 + 1 accounting reconciles the 15 trials listed in Table 1, with the nine trials fitted in the primary network (k = 9) and with the five vertices shown in Figure 2. Full characteristics are in Table 1. All were randomized, double-blind, placebo-controlled, parallel-group trials of a biologic added to background inhaled therapy in adults with COPD (FEV₁ ≈20-80% predicted). Entry criteria differed materially in baseline eosinophils, from ≥ 300 cells/µL required (dupilumab, MATINEE) to eosinophil-agnostic (astegolimab), a key transitivity consideration (see "Transitivity, secondary and safety outcomes").

**Table 1:** Characteristics of the 15 included trials Node eosinophil band is the analysis population feeding the network. All 15 RCTs of a biologic added to background inhaled therapy in adults with COPD (post-bronchodilator FEV₁/FVC <0.70; FEV₁ ≈20-80% predicted, GOLD 2-4). No head-to-head biologic comparisons exist, giving a star-shaped network. The benralizumab node uses the EOS ≥ 220/µL primary-analysis subgroup of GALATHEA/TERRANOVA. FRONTIER-4 (tozorakimab) reports only a COPDCompEx time-to-first-event hazard ratio and is not connected in the exacerbation network. COPD-HELP is the single acute-initiation (post-hospitalization) trial and was analysed separately. † Enrollment denominators follow the source convention (registry enrollment vs publication treated/as-treated) and did not enter synthesis; where arm-level counts were unavailable, only the total is shown. ‡ Annualized rate ratio for moderate or severe COPD exacerbations vs placebo, as reported and used in the network. Where ‡ marks an individual estimate, it is not a whole-randomized-population figure with a 95% CI: GALATHEA and TERRANOVA give the blood eosinophil ≥220 cells/µL primary-analysis subgroup; COURSE reports a 90% CI. ‡‡ COPD-HELP randomized totals are from the ClinicalTrials.gov participant-flow STARTED milestone (119 per arm); safety-population denominators were 119 for placebo and 118 for mepolizumab. § MATINEE used a variable follow-up of 52-104 weeks depending on enrollment date, rather than a fixed endpoint. ¶ The ≥220 cells/µL band for GALATHEA and TERRANOVA is a pre-specified primary-analysis subgroup, not an enrollment criterion; both enrolled without a blood eosinophil requirement. ** COPD-HELP is the only acute-initiation trial, randomizing at hospitalization for an exacerbation. Its eosinophil population is defined at the index event; it does not contribute to the maintenance network. Source data were deposited online.

Trial (NCT) (ref)	Agent · pathway	Phase	N randomized†	Treatment duration	Eosinophil entry criterion	Node eos band	Prior-exacerbation entry	Background therapy	Exacerbation RR vs placebo (95% CI)‡	RoB 2 overall	Contributes to
BOREAS [34] (NCT03930732)	Dupilumab · IL-4/13	3	939 (468/471)	52 wk	Blood eos ≥300/µL required	≥300	≥2 mod or ≥1 sev in prior yr	Triple (ICS+LABA+LAMA)	0.70 (0.58-0.86)	Low	Primary network (k = 9)
NOTUS [35] (NCT04456673)	Dupilumab · IL-4/13	3	935 (470/465)	52 wk	Blood eos ≥300/µL required	≥300	≥2 mod or ≥1 sev in prior yr	Triple	0.66 (0.54-0.82)	Low	Primary network (k = 9)
METREX [36] (NCT02105948)	Mepolizumab · IL-5	3	837† (arms NR)	52 wk	None (unselected)	Unselected	≥2 mod or ≥1 sev in prior yr	Triple (ICS-based)	0.98 (0.85-1.12)	Some concerns	Primary network (k = 9)
METREO [36] (NCT02105961)	Mepolizumab · IL-5	3	674† (arms NR)	52 wk	≥150/µL at screening OR ≥300/µL historical (enriched)	≥150	≥2 mod or ≥1 sev in prior yr	Triple	0.80 (0.65-0.98)	Some concerns	Primary network (k = 9)
MATINEE [17] (NCT04133909)	Mepolizumab · IL-5	3	804 (403/401)	52-104 wk (variable)§	≥300/µL at screening (+≥150 historical)	≥300	≥2 mod or ≥1 sev in prior yr	Triple (ICS-based)	0.79 (0.66-0.94)	Some concerns	Primary network (k = 9)
GALATHEA [25] (NCT02138916)	Benralizumab · IL-5Rα	3	1,656† (arms NR)	56 wk	None required; stratified/primary-analysis at ≥220/µL	≥220¶	≥2 mod or ≥1 sev in prior yr	Double or triple	0.83 (0.69-1.00)‡	Some concerns	Primary network (k = 9)
TERRANOVA [25] (NCT02155660)	Benralizumab · IL-5Rα	3	2,255† (arms NR)	56 wk	None required; stratified/primary-analysis at ≥220/µL	≥220¶	≥2 mod or ≥1 sev in prior yr	Double or triple	0.93 (0.78-1.10)‡	Some concerns	Primary network (k = 9)
ALIENTO [18] (NCT05037929)	Astegolimab · IL-33/ST2	2b	1,334 randomized; 1,301 initiated† (Q2W 433/Q4W 437/pbo 431)	52 wk	None (eosinophil-agnostic)	Unselected	≥2 mod or sev in 12 mo (within prior 24 mo)	ICS+LABA, LAMA+LABA, or triple	0.85 (0.72-1.00)	High	Primary network (k = 9)
ARNASA [18] (NCT05595642)	Astegolimab · IL-33/ST2	3	1,375† (Q2W 459/Q4W 459/pbo 457)	52 wk	None (eosinophil-agnostic)	Unselected	≥2 mod or sev in prior 12 mo	ICS+LABA, LAMA+LABA, or triple	0.85 (0.72-1.01)	High	Primary network (k = 9)
Itepekimab PoC [37] (NCT03546907)	Itepekimab · IL-33	2a	343 (172/171)	24-52 wk (variable)	None	Unselected	≥2 mod or ≥1 sev in prior yr	Double or triple	0.81 (0.61-1.07)	Low	Sensitivity layer only
FRONTIER-4 [19] (NCT04631016)	Tozorakimab · IL-33	2a	137 (69/68)	28 wk (primary wk 12)	None (BEC stratifier only)	Not in network‖	≥1 mod/sev in prior 24 mo	Double or triple	- (no rate ratio reported)‖	Some concerns*	Not connected - hazard ratio only
COPD-ST2OP [38] (NCT03615040)	Astegolimab · IL-33/ST2	2a	81† (arms NR)	48 wk	None (>170/µL subgroup stratifier only)	Unselected	≥2 mod/sev in prior 12 mo	BTS standard-of-care	0.78 (0.53-1.14)	Some concerns	Sensitivity layer only
COURSE [26] (NCT04039113)	Tezepelumab · TSLP	2a	333 as-treated (420 mg 165/pbo 168; registry enrolment 337)	52 wk	None	Unselected	≥2 mod/sev in prior yr	Medium/high-dose triple	0.83 (90% CI 0.64-1.06)‡	Some concerns	Sensitivity layer only
Benralizumab ph2a [39] (NCT01227278)	Benralizumab · IL-5Rα	2a	101 (51/50)	48-56 wk	Sputum eos ≥3% (blood unselected)	Unselected	≥1 exacerbation in prior yr	Standardized by FEV_1_ stratum	1.03 (0.67-1.58)	High	Sensitivity layer only
COPD-HELP [27] (NCT04075331)	Mepolizumab · IL-5	2/3	238 (119/119)‡‡	48 wk	Blood eos ≥300/µL (at index admission or prior 12 mo)	Separate**	≥2 in prior yr not required - enrolled at index acute event	ICS established pre-admission	analysed separately**	Some concerns	Analysed separately - acute initiation

Network Geometry

The primary maintenance exacerbation network is star-shaped: placebo plus four agents (dupilumab, mepolizumab, benralizumab, astegolimab), with no biologic-versus-biologic trials and therefore no closed loops (Figure 2). Itepekimab, tozorakimab, and tezepelumab have no published maintenance trials (all E6) and enter only through the phase 2 sensitivity layer; tozorakimab (FRONTIER-4) reports only a COPDCompEx hazard ratio, not a commensurable rate ratio, and is not connected in the exacerbation network.

**Figure 3:**
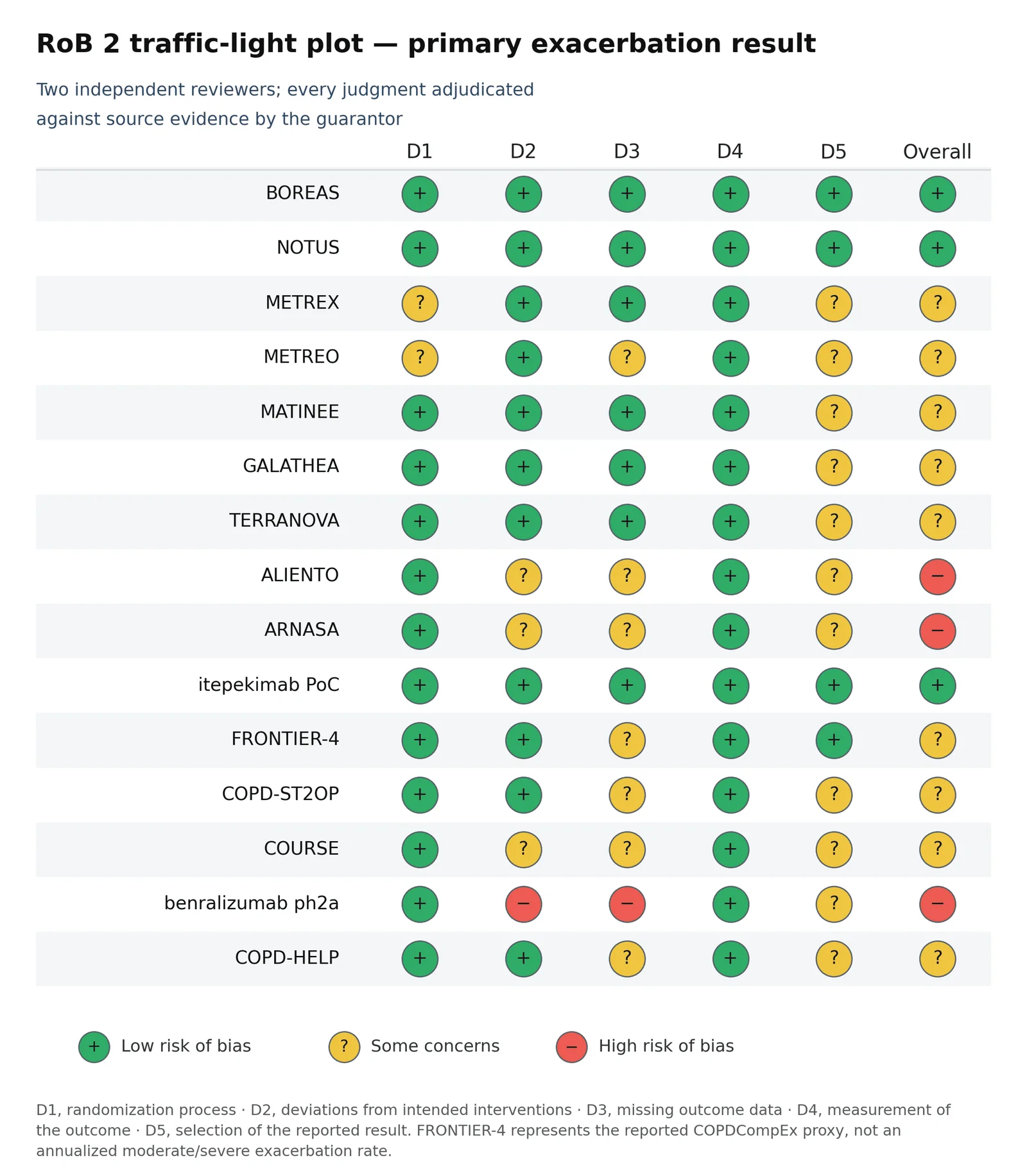
RoB 2 traffic light plot RoB 2 traffic light plot for the primary exacerbation result across all 15 included trials, showing domain-level and overall judgments for the effect of assignment to intervention. Judgments reflect independent assessment by two reviewers, followed by adjudication against the source evidence by the guarantor, who signed off every final judgment. All 15 included trials are shown, not only the nine in the primary network. FRONTIER-4 (tozorakimab) represents the reported COPDCompEx proxy result, not an annualized moderate/severe exacerbation rate. Domains: D1, bias arising from the randomization process; D2, bias due to deviations from intended interventions; D3, bias due to missing outcome data; D4, bias in measurement of the outcome; D5, bias in selection of the reported result. Symbols: + Low risk of bias; ? Some concerns; − High risk of bias

**Figure 4:**
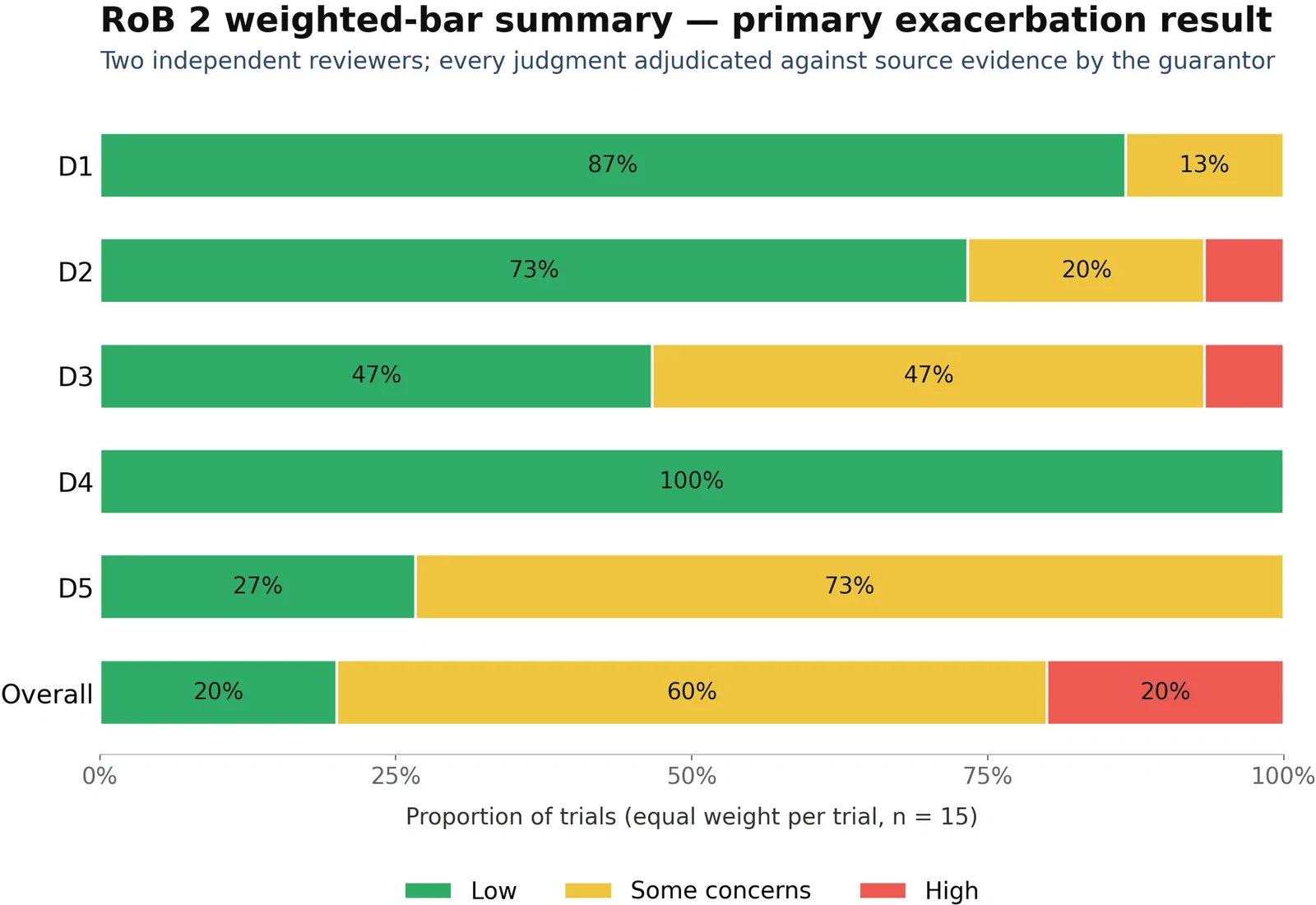
Weighted RoB 2 summary Weighted RoB 2 summary across all 15 included trials for domains D1-D5 and the overall judgment; percentages use one judgment per trial per domain, with equal weight per trial. Domains and Low/Some concerns/High judgments are as defined for Figure 3. The distribution locates the residual bias concern precisely: D4 is Low in every trial and D1 in 87%, whereas D5 is Some concerns in 73% of trials and D3 is split evenly between Low and Some concerns at 47% each. High-risk judgments are confined to single trials in D2 and D3. Overall, 20% of trials are Low risk, 60% Some concerns, and 20% High.

Risk of bias: RoB 2 overall judgments for the primary exacerbation result were "Low" (BOREAS, NOTUS), "Some concerns" (METREX, METREO, MATINEE, GALATHEA, TERRANOVA), and "High" (ALIENTO, ARNASA), with the phase 2 nodes "Low" to "High" (Figures 3-4). "Some concerns" judgments were driven mainly by statistical-analysis-plan timing (domain 5); the two astegolimab trials were "High" overall despite "Low" domain 1 (randomization) evidence recovered from the published manuscript. The top-ranked agent (dupilumab) rests on two "Low" RoB trials, whereas the astegolimab node is "High" RoB, relevant to certainty independent of the point estimates.

Figure 3 is the RoB 2 traffic light plot: One row per included trial, one column per domain (D1 randomization, D2 deviations from intended interventions, D3 missing outcome data, D4 outcome measurement, D5 selection of the reported result) plus an overall judgment, for the effect of assignment to intervention on the primary exacerbation outcome. It shows that the "Some concerns" judgments concentrate in D5, where 11 of the 15 trials are rated "Some concerns," while D1 and D4 are "Low" in nearly every trial; the residual bias concern in this network is therefore about analysis plan and reported result selection rather than randomization or outcome measurement. D3 (missing outcome data) is the secondary locus of concern, split evenly between "Low" and "Some concerns."

Figure 4 is the weighted RoB 2 summary: The same judgments were aggregated as the percentage of trials at each level within each domain and overall, one judgment per trial per domain with equal weight per trial. Read together, Figure 3 identifies which trial drives which concern, and Figure 4 gives the distribution across the evidence base. Both cover all 15 included trials, not only the nine in the primary network; FRONTIER-4 is shown against its reported COPDCompEx proxy result rather than an annualized exacerbation rate. Adjudication departed from at least one of the two independent assessments in 17 of the 90 judgments, and each override carries written, evidence-based support in the adjudication record deposited online. 

**Figure 5:**
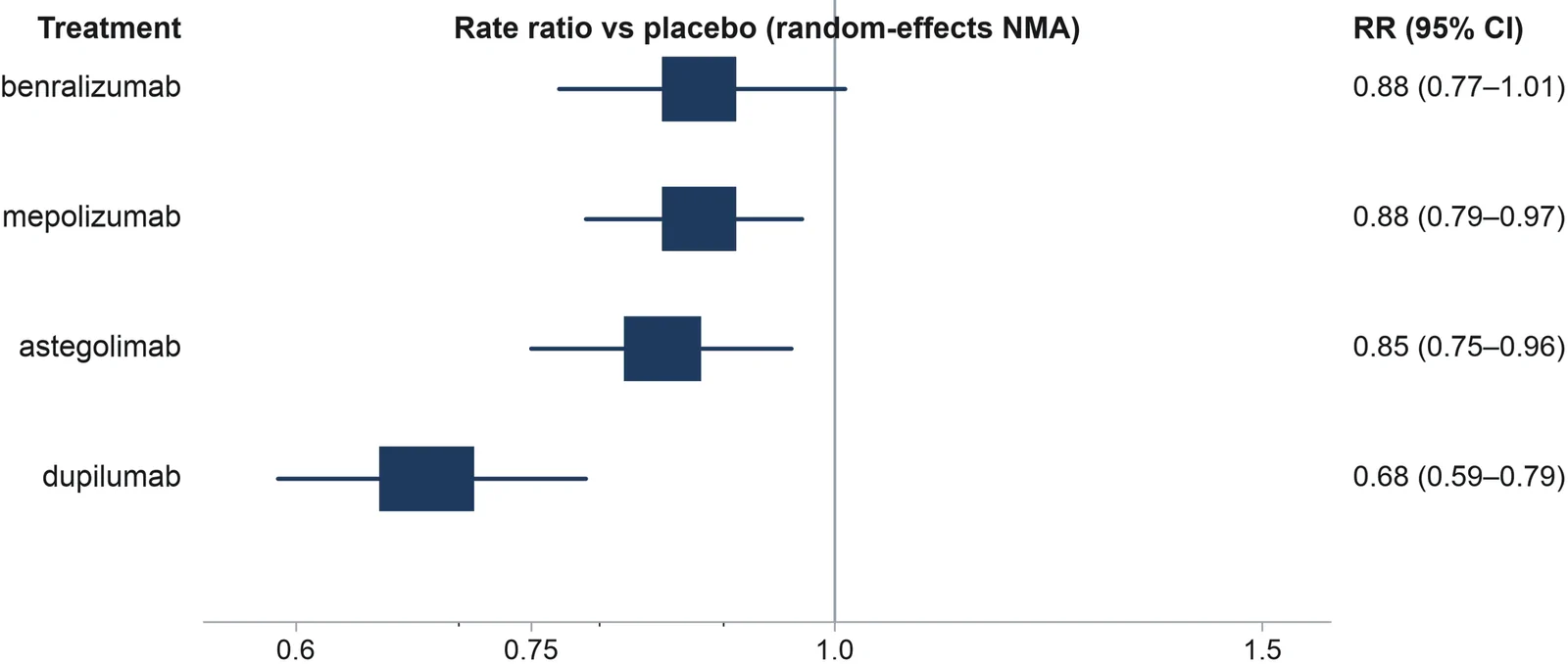
Random-effects network estimates for moderate/severe exacerbations versus placebo Random-effects network estimates for moderate/severe exacerbations versus placebo. The horizontal axis is the rate ratio versus placebo; the vertical line marks no effect at RR = 1; points to the left of that line favour the biologic. Squares are point estimates, horizontal lines are 95% confidence intervals. Dupilumab (RR 0.68, 95% CI 0.59-0.79) sits furthest left with an interval entirely below 1, the largest and most precisely estimated reduction. Astegolimab (0.85, 0.75-0.96) and mepolizumab (0.88, 0.79-0.97) also have intervals lying below 1. Benralizumab (0.88, 0.77-1.01) has an interval that crosses 1, so its reduction is not statistically significant. Estimates come from the nine-trial maintenance network of Figure 2 (k = 9), fitted with a common heterogeneity parameter; they are not based on all 15 included trials. No biologic-versus-biologic comparison is shown because no head-to-head trial exists.

Primary outcome, moderate/severe exacerbation rate: In the maintenance network (k = 9), all four agents reduced exacerbations versus placebo, three significantly (Table 2, Figure 5): dupilumab, RR: 0.68 (95% CI: 0.59-0.79), P-score: 0.9947, rank 1; astegolimab, RR: 0.85 (95% CI: 0.75-0.96), P-score: 0.5766, rank 2; and mepolizumab, RR: 0.88 (95% CI: 0.79-0.97), P-score: 0.4708, rank 3; benralizumab, RR: 0.88 (95% CI: 0.77-1.01), P-score: 0.4471, rank 4 (borderline, CI upper bound 1.007). The ranking is as follows: dupilumab > astegolimab > mepolizumab > benralizumab > placebo. Heterogeneity was low (τ² = 0.00087, I² = 9.68%, 95% CI: 0-77.08%). The pre-specified sanity check passed (dupilumab vs placebo 0.6808 ≈ 0.68). In the league table (Table 3), dupilumab was favored over every other agent in indirect network comparisons through placebo (vs astegolimab: 0.80, 95% CI: 0.66-0.97; vs mepolizumab: 0.78, 95% CI: 0.65-0.93; vs benralizumab: 0.77, 95% CI: 0.63-0.94), while astegolimab, mepolizumab, and benralizumab did not differ significantly from one another.

**Table 2:** Network meta-analysis estimates and ranking: primary maintenance exacerbation network Random-effects netmeta (v3.3.1), placebo-anchored, summary measure = rate ratio, k = 9 trials. The model is fitted on nine of the 15 included trials - those marked Primary network in Table 1 - and the trials contributing (k) column shows how the nine distribute across the four comparisons (2 + 3 + 2 + 2). A rate ratio below 1 favours the biologic; dupilumab showed the largest estimated reduction (RR: 0.68, a ≈32% lower exacerbation rate) and the three IL-5, IL-5Rα and ST2 agents clustered at RR: 0.85-0.88. Ranking best→worst by exacerbation-rate reduction: dupilumab > astegolimab > mepolizumab > benralizumab > placebo. Heterogeneity τ² = 0.00087, I² = 9.68% (95% CI: 0-77.08%) - low, common across the network. Between-designs inconsistency not estimable (star network, no closed loops). Pre-specified sanity check (dupilumab vs placebo ≈0.68) passed at 0.6808. All four agents reduced exacerbations versus placebo; three reached significance (benralizumab borderline, upper CI: 1.007). All active-active comparisons are indirect through placebo and depend on transitivity. Source data, the analysis dataset, and the code reproducing every estimate are deposited online.

Rank	Agent (pathway)	Direct trials (ref)	Trials contributing (k)	RR (95% CI) vs placebo	95% prediction interval	P-score	p-value	Node RoB 2
1	Dupilumab (IL-4/13)	BOREAS [34], NOTUS [35]	2	0.68 (0.59-0.79)	0.55-0.84	0.9947	<0.0001	Low, Low
2	Astegolimab (IL-33/ST2)	ALIENTO [18], ARNASA [18]	2	0.85 (0.75-0.96)	0.71-1.02	0.5766	0.0107	High, High
3	Mepolizumab (IL-5)	METREX [36], METREO [36], MATINEE [17]	3	0.88 (0.79-0.97)	0.75-1.02	0.4708	0.0114	Some concerns ×3
4	Benralizumab (IL-5Rα)	GALATHEA [25], TERRANOVA [25]	2	0.88 (0.77-1.01)	0.73-1.07	0.4471	0.0636	Some concerns ×2
5	Placebo (reference)	-	9 (comparator in every trial)	1.00 (ref)	-	0.0107	-	-

**Table 3:** League table: network estimates for all pairwise comparisons Random-effects netmeta (v3.3.1), ordered by rank; reciprocal cells computed from full-precision estimates before rounding. Each cell is the network rate ratio of moderate/severe exacerbations for the ROW treatment versus the COLUMN treatment; RR <1 favours the row treatment. No active biologic was compared directly with another; every active-versus-active cell is an indirect network estimate through the common placebo comparator and depends on transitivity ("pairwise" here does not mean head-to-head trial evidence). Dupilumab is favored over every other agent (vs astegolimab: 0.80, 95% CI: 0.66-0.97; vs mepolizumab: 0.78, 95% CI: 0.65-0.93; vs benralizumab: 0.77, 95% CI: 0.63-0.94; vs placebo: 0.68, 95% CI: 0.59-0.79); astegolimab, mepolizumab, and benralizumab do not differ significantly. Source data were deposited online.

Row vs Column	Dupilumab	Astegolimab	Mepolizumab	Benralizumab	Placebo
Dupilumab	-	0.80 (0.66-0.97)	0.78 (0.65-0.93)	0.77 (0.63-0.94)	0.68 (0.59-0.79)
Astegolimab	1.25 (1.03-1.52)	-	0.97 (0.83-1.14)	0.96 (0.80-1.16)	0.85 (0.75-0.96)
Mepolizumab	1.29 (1.07-1.54)	1.03 (0.88-1.21)	-	0.99 (0.84-1.17)	0.88 (0.79-0.97)
Benralizumab	1.30 (1.06-1.58)	1.04 (0.86-1.24)	1.01 (0.85-1.19)	-	0.88 (0.77-1.01)
Placebo	1.47 (1.27-1.70)	1.18 (1.04-1.33)	1.14 (1.03-1.26)	1.13 (0.99-1.29)	-

Sensitivity analyses: Dupilumab remained first in every sensitivity analysis and all three analytic engines (Table 2; the full set is in Table 4). Table 4 lists the six executed sensitivity models and one pre-specified analysis that could not be run, giving the number of trials in each model and its effect on the primary ranking, so robustness can be judged against the specific perturbation applied rather than asserted. Adding the phase 2 layer (k = 13), restricting to phase 3 trials (k = 8), common- versus random-effects, and leave-one-agent-out preserved the four-agent order. Bayesian re-analysis in gemtc and multinma reproduced the frequentist point estimates and order to the third decimal (dupilumab, RR: 0.680-0.681; SUCRA ≈0.97 [40]); R-hat was <1.05, although the multinma fit retained 11 post-warmup divergent transitions, so it is a corroborative sensitivity rather than the primary result. The complete ranking was not stable: the astegolimab dose-schedule sensitivity weakened astegolimab to RR: 0.874 (p = 0.0555) and exchanged its position with mepolizumab. The pre-specified eosinophil network meta-regression was not estimable (rank-deficient: three of four agents have no within-node eosinophil-threshold variation). 

**Table 4:** Sensitivity analyses for the primary maintenance exacerbation network Random-effects netmeta (v3.3.1). k = number of trials in the corresponding sensitivity model. Not-run analyses (node-splitting/design-by-treatment inconsistency; comparison-adjusted funnel plot/Egger's test) are documented as not estimable given the star network geometry and the absence of any contrast with ≥10 studies; reporting bias for these contrasts was instead assessed narratively. —: A pre-specified analysis that carries no sequence number because it could not be estimated. Source data, all sensitivity scripts, and the fitted model objects were deposited online.

#	Sensitivity analysis	k	Effect on the primary ranking
1	Full network incl. phase-2 layer (adds itepekimab, tezepelumab)	13	Primary four-agent order preserved; full order dupilumab(1) > itepekimab(2) > astegolimab(3) > tezepelumab(4) > mepolizumab(5) > benralizumab(6). New phase-2 nodes non-significant, wide CIs.
2	Phase-3-only (drops ALIENTO, phase 2b)	8	Ranking unchanged; astegolimab node (ARNASA alone) CI widens to non-significant; τ² rises (precision loss, not inconsistency).
3	Common-effect vs random-effects	9	Near-identical (I² = 9.68%); ranking unchanged. Robust to model choice.
4	Leave-one-agent-out	6-7	Relative order among surviving agents identical in all 12 scenarios (order_preserved = TRUE; rank_shift = 0).
5	Bayesian re-analysis - gemtc/JAGS and pre-specified multinma/Stan	9	Both reproduce the frequentist point estimates/order; R-hat/PSRF <1.05. The multinma fit retained 11 divergent transitions, disclosed as a sensitivity-model limitation.
6	Astegolimab dose-schedule (Q4W-for-both substitution)	9	The one analysis that changes the ranking: astegolimab weakens to RR 0.874 (P=0.0555) and swaps rank with mepolizumab (2→3).
—	Eosinophil network meta-regression	9	Pre-specified but not estimable (rank-deficient: 3 of 4 agents have no within-node eosinophil-threshold variation). Confirms eosinophil-population mixing as the key indirectness limitation.

Read as a set, Table 4 separates what is robust from what is not. The four-agent order survives every perturbation of both the evidence base and the model: adding the phase 2 layer (k = 13), restricting to phase 3 trials (k = 8), switching from random- to common-effects, removing each agent in turn (k = 6-7 across all 12 leave-one-out scenarios, order preserved, rank shift 0), and re-fitting in two independent Bayesian engines. Only one analysis changes the ranking - the astegolimab dose-schedule substitution (analysis 6), which weakens astegolimab to RR: 0.874 (p = 0.0555) and exchanges ranks 2 and 3 with mepolizumab. That is the reason the complete ordering of the three non-dupilumab agents is reported as unstable and carries low certainty in the "GRADE-CINeMA assessment" table. The pre-specified eosinophil network meta-regression could not be estimated because three of the four agents have no within-node variation in eosinophil entry threshold, which corroborates eosinophil-population mixing as the leading indirectness limitation rather than resolving it. Node-splitting, design-by-treatment inconsistency testing, and comparison-adjusted funnel plots are documented in Table 4 as not estimable, given the star geometry and the absence of any contrast reaching 10 studies.

Transitivity, secondary, and safety outcomes: The primary whole-population network mixes distinct baseline-eosinophil populations (eos ≥300, ≥220 subgroup, ≥150-enriched, and unselected nodes); because the network is star-shaped, this indirectness cannot be tested statistically, and the eosinophil-stratified networks were not estimable, the leading limitation (see Limitations). Secondary efficacy and safety estimates were retained at study level and are reported in full in Table 5, which gives individual study results for all 15 included trials across the 12 prespecified outcome domains - 180 trial × domain rows in one continuous table, covering the exacerbation rate, pre- and post-bronchodilator FEV₁, SGRQ, and the safety domains - with the effect measure, time point, denominator, and precision exactly as reported in each source. Table 5 is the evidence base for the decision not to pool any secondary outcome, and reading it makes the reason visible. Definitions and time points do not align across trials: FEV₁ is reported pre-bronchodilator in some trials and post-bronchodilator in others, at week 12 in the phase 2 trials and at weeks 52 or 104 in the maintenance trials, and SGRQ is reported as a least-squares mean difference in some trials with no obtainable standard error or confidence interval in others. Many cells are NR - no trial-specific numeric result was present in the source, or the corresponding registry field was unpopulated - and the pattern of missingness is outcome-dependent rather than trial-dependent. Values marked "Derived: Yes" are the only reconstructions from arm-level data; every other value is transcribed as reported, and randomized, treated, safety, and analysis denominators are reproduced as labelled in each source rather than forced into a single convention. No secondary outcome therefore yielded a set of trials sharing a commensurable effect measure, scale, and time point sufficient for a valid connected network, and all secondary results are reported narratively and at study level. The separate acute-initiation trial (COPD-HELP) is reported apart from the network. Reporting bias for the primary synthesis could not be assessed statistically (no contrast reaches ≥10 studies; largest = mepolizumab, k = 3); it was assessed narratively via registry-vs-publication outcome checking. The primary annualized exacerbation rate was consistently reported in the registered pivotal trials, with no selective outcome reporting identified for this outcome.

**Table 5:** Individual study results for all included trials Coverage is complete at the protocol-domain level - 15 included trials × 12 prespecified outcome domains = 180 rows, presented in one continuous table. NR: not reported/not obtainable (no trial-specific numeric result was present in the extraction workbook, or the corresponding registry field was not populated). “Derived: Yes” is used only when the workbook explicitly documents a reconstruction or calculation from arm-level data; “No” indicates the value is transcribed as reported. Randomized, treated, safety, and analysis denominators are reproduced as labelled in the source and are not forced into a single convention. Full abbreviation list is given at the end of the table. FEV_1_: forced expiratory volume in one second Source data, including the extraction workbook and the per-trial provenance record for all 15 trials, are deposited online.

Trial	Arm(s)/denominator	Prespecified outcome/timepoint	Per-arm summary	Comparative effect and precision	Source(s)	Derived?	
S1.1 BOREAS (NCT03930732) [34]							
BOREAS	Active: 468 (dupilumab 300 mg q2w, single active arm). Placebo: 471	Moderate/severe exacerbation rate. Outcome-specific timepoint NR; trial follow-up context: 52-week treatment period (~68 weeks total, including 4-week screening and 12-week follow-up)	Exacerbation - adjusted annualized rate, active arm (dupilumab 300 mg q2w): 0.78 (95% CI: 0.64-0.93). Exacerbation - adjusted annualized rate, placebo arm: 1.10 (95% CI: 0.93-1.30)	Eosinophil 150-<300 cells/µL - exacerbation RR vs placebo (95% CI): Not obtainable as a BOREAS-only estimate - BOREAS’s ≥300 cells/µL eligibility threshold means no 150-<300 subgroup exists within BOREAS’s own randomized population. A POOLED BOREAS+NOTUS sensitivity-analysis estimate does exist and is verified: RR 0.67 (95% CI: 0.52-0.87) for this eosinophil stratum (vs mepolizumab METREX mITT high-stratum RR 0.92, 95% CI: 0.73-1.16), reported in Bhatt et al. [35], Pulm Ther 2025. This pooled figure must NOT be mislabeled as a BOREAS-specific result for this trial node (not obtainable). Eosinophil-subgroup - exacerbation-rate-reduction interaction (not a per-stratum RR): Post-hoc BOREAS analysis: exacerbation-rate reduction with dupilumab was numerically greater in patients with higher baseline blood eosinophil count (interaction p=0.0056) and higher baseline FeNO (interaction p=0.043). No per-stratum rate ratios or CIs are reported - only the interaction p-values; BOREAS enrolled only ≥300 cells/µL patients by design. Exacerbation - rate ratio vs placebo, 95% CI: rate ratio: 0.70 (95% CI: 0.58-0.86), p<0.001	ClinicalTrials.gov NCT03930732; PMC12992723; PMID 41004068; PMID 40651490; PMID 37272521	No - transcribed as reported; S1 adds no calculation.	
	Active: 468 (dupilumab 300 mg q2w, single active arm). Placebo: 471	Pre-bronchodilator FEV1 week 12, week 52, week 12, week 52	FEV1 - LS-mean change from baseline at week 12, active arm: +160 mL (95% CI: 126-195), pre-bronchodilator FEV1 at week 12 (dupilumab arm). No numeric week-52 value is reported in the abstract. FEV1 - LS-mean change from baseline at week 12, placebo arm: +77 mL (95% CI: 42-112), pre-bronchodilator FEV1 at week 12 (placebo arm). No numeric week-52 value is reported in the abstract - only described qualitatively as “sustained through week 52.” FEV1 - LS-mean change from baseline at week 52 (placebo and active arms) and LS-mean difference at week 52: Change from baseline in pre-BD FEV1 at week 52 (liters): placebo=0.070; dupilumab 300 mg q2w=0.153; ANALYSIS: LS Mean Difference=0.083, CI: 0.038-0.128, p=0.0003	Eosinophil <150 cells/µL - any outcome (exacerbation RR, FEV1, SGRQ, E-RS): Not obtainable/not applicable - BOREAS’s inclusion criterion restricts enrollment to blood eosinophils ≥300 cells/µL, so no <150 cells/µL subgroup exists within this trial’s randomized population by design (not obtainable). FEV1 - LS-mean difference vs placebo (mL) at week 12, 95% CI: 83 mL (95% CI: 42-125), p<0.001, at week 12. Abstract states this difference was “sustained through week 52” but reports no separate numeric week 52 LS-mean difference. Note: an apparently similar “83.4 mL (95% CI: 36.1-130.7)” figure in Bhatt et al. [35], Pulm Ther 2025 is a DIFFERENT statistic - a dupilumab-vs-mepolizumab indirect-comparison estimate at week 52, not BOREAS’s own week 12 vs placebo results; the resemblance in the rounded headline number is coincidental and must not be logged as corroboration. FeNO ≥20 ppb subgroup - FEV1 and exacerbation-rate outcomes: Pre-BD FEV1 Wk12 (FeNO>=20, liters): placebo=0.108; dupilumab=0.232; LS mean difference=0.124, CI: 0.045-0.203, p=0.0022. Pre-BD FEV1 week 52 (FeNO>=20, liters): placebo=0.120; dupilumab=0.247; LS mean difference=0.127, CI: 0.042-0.212, p=0.0034. Annualized moderate/severe exacerbation rate (FeNO>=20, per participant-year): placebo=1.117 (0.831-1.502); dupilumab=0.699 (0.510-0.958); risk difference=-0.418, CI: -0.728-0.109, p=0.0052	ClinicalTrials.gov NCT03930732; PMID 37272521; PMC12992723; PMID 41004068	No - transcribed as reported; S1 adds no calculation.	
	Active: 468 (dupilumab 300 mg q2w, single active arm). Placebo: 471	Post-bronchodilator FEV1. Outcome-specific timepoint NR; trial follow-up context: 52-week treatment period (~68 weeks total, including 4-week screening and 12-week follow-up)	NR/not obtainable - no trial-specific numeric result for this domain was present in the extraction workbook.	NR/not obtainable - no trial-specific numeric result for this domain was present in the extraction workbook.	Extraction workbook (no matching trial-specific outcome row).	Not applicable - NR/not obtainable.	
	Active: 468 (dupilumab 300 mg q2w, single active arm). Placebo: 471	SGRQ total score week: 52	SGRQ - LS-mean change from baseline at week 52, active arm: -9.7 (95% CI: -11.3 to -8.1), SGRQ - LS-mean change from baseline at week 52, placebo arm: -6.4 (95% CI: -8.0 to -4.8)	SGRQ - LS-mean difference vs placebo at week 52, 95% CI: -3.4 (95% CI: -5.5 to -1.3), p=0.002	PMID 37272521	No - transcribed as reported; S1 adds no calculation.	
	Active: 468 (dupilumab 300 mg q2w, single active arm). Placebo: 471	E-RS:COPD week 52	E-RS - LS-mean change from baseline at week 52, active arm: -2.7 (95% CI: -3.2 to -2.2)E-RS - LS-mean change from baseline at week 52, placebo arm: -1.6 (95% CI: -2.1 to -1.1)	E-RS - LS-mean difference vs placebo at week 52, 95% CI: -1.1 (95% CI: -1.8 to -0.4), p=0.001	PMID 37272521	No - transcribed as reported; S1 adds no calculation.	
	Active: 468 (dupilumab 300 mg q2w, single active arm). Placebo: 471	Time to first exacerbation. Outcome-specific timepoint NR; trial follow-up context: 52-week treatment Period (~68 weeks total, including 4-week screening and 12-week follow-up)	NR/not obtainable - no trial-specific numeric result for this domain was present in the extraction workbook.	NR/not obtainable - no trial-specific numeric result for this domain was present in the extraction workbook.	Extraction workbook (no matching trial-specific outcome row).	Not applicable - NR/not obtainable.	
	Active: 468 (dupilumab 300 mg q2w, single active arm). Placebo: 471	Rescue-medication use. Outcome-specific timepoint NR; trial follow-up context: 52-week treatment period (~68 weeks total including 4-week screening and 12-week follow-up)	NR/not obtainable - no trial-specific numeric result for this domain was present in the extraction workbook.	NR/not obtainable - no trial-specific numeric result for this domain was present in the extraction workbook.	Extraction workbook (no matching trial-specific outcome row).	Not applicable - NR/not obtainable.	
	Active: 468 (dupilumab 300 mg q2w, single active arm). Placebo: 471	Any treatment-emergent adverse event. Outcome-specific timepoint NR; trial follow-up context: 52-week treatment period (~68 weeks total, including 4-week screening and 12-week follow-up)	Safety - TEAE, n/N per arm: Any TEAE: placebo=359; dupilumab 300 mg q2w=365 (N = safety population: placebo=470; dupilumab 300 mg q2w=469)	NR/not obtainable - no comparative effect with precision was present; see per-arm summary.	ClinicalTrials.gov NCT03930732	No - transcribed as reported; S1 adds no calculation.	
	Active: 468 (dupilumab 300 mg q2w, single active arm). Placebo: 471	Serious adverse events. Outcome-specific timepoint NR; trial follow-up context: 52-week treatment period (~68 weeks total, including 4-week screening and 12-week follow-up)	Safety - SAE, n/N per arm: placebo: serious. Affected=74/470; dupilumab=300 mg q2w: serious. Affected=65/469	NR/not obtainable - no comparative effect with precision was present; see per-arm summary.	ClinicalTrials.gov NCT03930732	No - transcribed as reported; S1 adds no calculation.	
	Active: 468 (dupilumab 300 mg q2w, single active arm). Placebo: 471	AEs leading to discontinuation. Outcome-specific timepoint NR; trial follow-up context: 52-week treatment period (~68 weeks total, including 4-week screening and 12-week follow-up)	Safety - discontinuation due to AE, n/N per arm: placebo 9/470 started; dupilumab: 7/469 started withdrew because of an adverse event.	NR/not obtainable: no comparative effect with precision was present; see per-arm summary.	ClinicalTrials.gov NCT03930732	No - transcribed as reported; S1 adds no calculation.	
	Active: 468 (dupilumab 300 mg q2w, single active arm). Placebo: 471	All-cause death. Outcome-specific timepoint NR; trial follow-up context: 52-week treatment period (~68 weeks total, including 4-week screening and 12-week follow-up)	Safety - death, n/N per arm: placebo: deaths=9/470; dupilumab 300 mg q2w: deaths=8/469	NR/not obtainable - no comparative effect with precision was present; see per-arm summary.	ClinicalTrials.gov NCT03930732	No - transcribed as reported; S1 adds no calculation.	
	Active: 468 (dupilumab 300 mg q2w, single active arm). Placebo: 471	AEs of special interest. Outcome-specific timepoint NR; trial follow-up context: 52-week treatment period (~68 weeks total, including 4-week screening and 12-week follow-up)	NR/not obtainable - no trial-specific numeric result for this domain was present in the extraction workbook.	NR/not obtainable - no trial-specific numeric result for this domain was present in the extraction workbook.	Extraction workbook (no matching trial-specific outcome row).	Not applicable - NR/not obtainable.	
S1.2 NOTUS (NCT04456673) [35]							
NOTUS	Active: 470 randomized to dupilumab 300 mg SC q2w add-on to background therapy; 468 randomized-and-treated; 469 in the safety population. Placebo: 465 randomized to placebo; 465 randomized-and-treated; 464 in the Safety Population (1 participant allocated to placebo but inadvertently dosed with dupilumab 300 mg q2w on Day 113, so analyzed under the dupilumab arm for safety)	Moderate/severe exacerbation rate. Outcome-specific timepoint NR; trial follow-up context: 52-week double-blind treatment period, within a total study duration of ~68 weeks (4-week screening + 52-week treatment + 12-week follow-up); primary/key secondary endpoints assessed to week 52 (FEV1 also assessed at week 12). Separately, AE/SAE/mortality data collection window extended to last dose of study treatment + 98 days (up to 506 days).	Exacerbation - adjusted annualized rate, active arm (dupilumab 300 mg q2w): 0.859 moderate-or-severe exacerbations/participant-year (95% CI: 0.699-1.057), ITT population N=470 (CT.Gov exact); rounds to the NEJM abstract’s reported 0.86 (95% CI: 0.70-1.06). Exacerbation - adjusted annualized rate, placebo arm: 1.295 moderate-or-severe exacerbations/participant-year (95% CI: 1.048-1.600), ITT population N=465 (CT.Gov exact); rounds to the NEJM abstract’s reported 1.30 (95% CI: 1.05-1.60)	Eosinophil subgroup <150 cells/µL - exacerbation outcome estimates: Not applicable - NOTUS enrolled only patients with blood eosinophils ≥300 cells/µL by design; no <150 cells/µL subgroup exists in this trial and no such outcome measure is registered on CT.Gov (not obtainable).Eosinophil ≥300 cells/µL (whole trial population, by design) - exacerbation outcome estimates: Duplicates the primary exacerbation-rate outcome (NOTUS enrolled ONLY eosinophils ≥300 by design, so the whole-ITT-population result IS this “subgroup”): rate ratio: 0.66 (95% CI: 0.54-0.82), p<0.001 (NEJM abstract); risk difference -0.435/participant-year (95% CI: -0.682 to -0.188), p=0.0002 (CT.Gov). Exacerbation - rate ratio (or risk difference) vs placebo, 95% CI: two distinct, non-contradictory statistics from the same underlying negative binomial Model: (1) rate ratio 0.66 (95% CI: 0.54-0.82), p<0.001 - reported in the NEJM abstract. (2) Risk difference -0.435 exacerbations/participant-year (95% CI: -0.682 to -0.188), p=0.0002 - the statistic posted on CT.Gov, labelled “Risk Difference (RD)”. Internal consistency: 0.859/1.295 = 0.663 ≈ 0.66, confirming both derive from the same model outputs. Supplementary - BOREAS+NOTUS pooled analysis exacerbation rate (context only, NOT NOTUS-specific): pooled dupilumab n=938, placebo n=936 (1874 total, from BOREAS+NOTUS combined); pooled annualized moderate/severe exacerbation rate: 0.794 (dupilumab) vs 1.156 (placebo), IRR: 0.687 (95% CI: 0.595-0.793), p<0.0001; severe-exacerbation-only rate not significantly reduced (0.084 vs 0.124/yr, RR: 0.674, 95% CI: 0.438-1.037, p=0.073); pooled former-smoker proportion 1316/1874 (70.2%). TEAE/SAE/discontinuation-due-to-AE/death described as “similar between groups” with no n/N given in the abstract.	ClinicalTrials.gov NCT04456673; PMID 38767614; PMID 39900091	No - transcribed as reported; S1 adds no calculation.	
	Active: 470 randomized to dupilumab 300 mg SC q2w add-on to background therapy; 468 randomized-and-treated; 469 in the safety population. Placebo: 465 randomized to placebo; 465 randomized-and-treated; 464 in the safety population (1 participant allocated to placebo but inadvertently dosed with dupilumab 300 mg q2w on day 113, so analyzed under the dupilumab arm for safety)	Pre-bronchodilator FEV1 week 12, week 52	FEV1 - LS-mean change from baseline to week 12, active arm (dupilumab): 0.139 L/139 mL (SE 0.017), n=464 (CT.Gov exact); consistent with NEJM abstract’s 139 mL (95% CI: 105-173)FEV1: LS-mean change from baseline to week 12, placebo arm: 0.057 L/57 mL (SE 0.017), n=461 (CT.Gov exact); consistent with NEJM abstract’s 57 mL (95% CI: 23-91); FEV1 - LS-mean change from baseline, active arm (week 52): 0.115 L (SE 0.021), n=359, week 52 (CT.Gov exact). FEV1 - LS-mean change from baseline, placebo arm (week 52): 0.054 L (SE 0.020), n=356, week 52 (CT.Gov exact)	FEV1 - LS-mean difference vs placebo (mL), week 12: 82 mL (0.082 L) difference vs placebo, 95% CI: 40-124 mL (0.040-0.124 L), p=0.0001 (CT.Gov exact); NEJM abstract reports the same point estimate as 82 mL, P<0.001 (abstract does not give the CI for the between-group difference). FEV1: LS-mean difference vs placebo (mL), week 52: 62 mL (0.062 L) difference vs placebo, 95% CI: 11-113 mL (0.011-0.113 L), p=0.0182 (CT.Gov exact); NEJM abstract reports the same point estimate as 62 mL, p=0.02 (abstract does not give the CI).	ClinicalTrials.gov NCT04456673; PMID 38767614	No: transcribed as reported; S1 adds no calculation.	
	Active: 470 randomized to dupilumab 300 mg SC q2w add-on to background therapy; 468 randomized-and-treated; 469 in the safety population. Placebo: 465 randomized to placebo; 465 randomized-and-treated; 464 in the safety population (1 participant allocated to placebo but inadvertently dosed with dupilumab 300 mg q2w on day 113, so analyzed under the dupilumab arm for safety)	Post-bronchodilator FEV1. Outcome-specific timepoint NR; trial follow-up context: 52-week double-blind treatment period, within a total study duration of ~68 weeks (4-week screening + 52-week treatment + 12-week follow-up); primary/key secondary endpoints assessed to week 52 (FEV1 also assessed at week 12). Separately, AE/SAE/mortality data collection window extended to last dose of study treatment + 98 days (up to 506 days).	NR/not obtainable - no trial-specific numeric result for this domain was present in the extraction workbook.	NR/not obtainable - no trial-specific numeric result for this domain was present in the extraction workbook.	Extraction workbook (no matching trial-specific outcome row).	Not applicable - NR/not obtainable.	
	Active: 470 randomized to dupilumab 300 mg SC q2w add-on to background therapy; 468 randomized-and-treated; 469 in the safety population. Placebo: 465 randomized to placebo; 465 randomized-and-treated; 464 in the safety population (1 participant allocated to placebo but inadvertently dosed with dupilumab 300 mg q2w on day 113, so analyzed under the dupilumab arm for safety)	SGRQ total score week 52, 52 weeks	SGRQ - LS-mean change from baseline, active arm (dupilumab): -9.816 points (SE 0.920), n=350, week 52, ITT-with-opportunity-to-reach - week 52 population (CT.Gov exact). Real, accurate figure - same significance caveat as the placebo-arm SGRQ row applies to interpretation. SGRQ - LS-mean change from baseline, placebo arm: -6.444 points (SE 0.922), n=342, week 52, ITT-with-opportunity-to-reach-week-52 population (CT.Gov exact). This posted number is real and accurate but see the paired “SGRQ - LS-mean difference vs placebo” row for the required significance caveat.	SGRQ - LS-mean difference vs placebo, SE/95% CI: CT.Gov posts a nominal LS-mean difference of -3.371 points (95% CI: -5.811 to -0.931), nominal P=0.0068. However, the trial’s own peer-reviewed NEJM publication states: “No significant between-group difference was observed in the change in SGRQ scores from baseline to 52 weeks.” Both are accurate and reconcilable: a pre-specified hierarchical (gatekeeping) multiplicity-adjustment testing procedure covered the primary endpoint plus the first 4 secondary endpoints tested sequentially; the SGRQ endpoint’s position in that sequence meant it did not clear the multiplicity-adjusted significance bar even though its raw/nominal 95% CI: excludes zero.	ClinicalTrials.gov NCT04456673; PMID 38767614	No - transcribed as reported; S1 adds no calculation.	
	Active: 470 randomized to dupilumab 300 mg SC q2w add-on to background therapy; 468 randomized-and-treated; 469 in the safety population. Placebo: 465 randomized to placebo; 465 randomized-and-treated; 464 in the safety population (1 participant allocated to placebo but inadvertently dosed with dupilumab 300 mg q2w on day 113, so analyzed under the dupilumab arm for safety).	E-RS:COPD. Outcome-specific timepoint NR; trial follow-up context: 52-week double-blind treatment period, within a total study duration of ~68 weeks (4-week screening + 52-week treatment + 12-week follow-up); primary/key secondary endpoints assessed to week 52 (FEV1 also assessed at week 12). Separately, AE/SAE/mortality data collection window extended to last dose of study treatment + 98 days (up to 506 days).	E-RS - LS-mean change from baseline, active arm: NR/not obtainable - workbook value cell is empty (not obtainable).E-RS - LS-mean change from baseline, placebo arm: NR/not obtainable - workbook value cell is empty (not obtainable).	E-RS - LS-mean difference vs placebo, SE/95% CI: NR/not obtainable - workbook value cell is empty (not obtainable).	Same as E-RS placebo-arm rowClinicalTrials.gov NCT04456673Same as E-RS rows above	Not applicable - NR/not obtainable.	
	Active: 470 randomized to dupilumab 300 mg SC q2w add-on to background therapy; 468 randomized-and-treated; 469 in the safety population. Placebo: 465 randomized to placebo; 465 randomized-and-treated; 464 in the Safety Population (1 participant allocated to placebo but inadvertently dosed with dupilumab 300 mg q2w on day 113, so analyzed under the dupilumab arm for safety).	Time to first exacerbation. Outcome-specific timepoint NR; trial follow-up context: 52-week double-blind treatment period, within a total study duration of ~68 weeks (4-week screening + 52-week treatment + 12-week follow-up); primary/key secondary endpoints assessed to week 52 (FEV1 also assessed at week 12). Separately, AE/SAE/mortality data collection window extended to last dose of study treatment + 98 days (up to 506 days).	NR/not obtainable - no trial-specific numeric result for this domain was present in the extraction workbook.	NR/not obtainable - no trial-specific numeric result for this domain was present in the extraction workbook.	Extraction workbook (no matching trial-specific outcome row).	Not applicable - NR/not obtainable.	
	Active: 470 randomized to dupilumab 300 mg SC q2w add-on to background therapy; 468 randomized-and-treated; 469 in the safety population. Placebo: 465 randomized to placebo; 465 randomized-and-treated; 464 in the safety population (1 participant allocated to placebo but inadvertently dosed with dupilumab 300 mg q2w on Day 113, so analyzed under the dupilumab arm for safety).	Rescue-medication use. Outcome-specific timepoint NR; trial follow-up context: 52-week double-blind treatment period, within a total study duration of ~68 weeks (4-week screening + 52-week treatment + 12-week follow-up); primary/key secondary endpoints assessed to week 52 (FEV1 also assessed at week 12). Separately, AE/SAE/mortality data collection window extended to last dose of study treatment + 98 days (up to 506 days).	NR/not obtainable - no trial-specific numeric result for this domain was present in the extraction workbook.	NR/not obtainable - no trial-specific numeric result for this domain was present in the extraction workbook.	Extraction workbook (no matching trial-specific outcome row).	Not applicable - NR/not obtainable.	
	Active: 470 randomized to dupilumab 300 mg SC q2w add-on to background therapy; 468 randomized-and-treated; 469 in the safety population. Placebo: 465 randomized to placebo; 465 randomized-and-treated; 464 in the safety population (1 participant allocated to placebo but inadvertently dosed with dupilumab 300 mg q2w on day 113, so analyzed under the dupilumab arm for safety)	Any treatment-emergent adverse event. Outcome-specific timepoint NR; trial follow-up context: 52-week double-blind treatment period, within a total study duration of ~68 weeks (4-week screening + 52-week treatment + 12-week follow-up); primary/key secondary endpoints assessed to week 52 (FEV1 also assessed at week 12). Separately, AE/SAE/mortality data collection window extended to last dose of study treatment + 98 days (up to 506 days).	Safety - TEAE, n/N per arm: placebo: 330/464 (71.1%); dupilumab: 322/469 (68.7%). Safety population (TEAE/TESAE registered as one combined CT.Gov outcome measure, not two separate ones).	NR/not obtainable - no comparative effect with precision was present; see per-arm summary.	ClinicalTrials.gov NCT04456673	No - transcribed as reported; S1 adds no calculation.	
	Active: 470 randomized to dupilumab 300 mg SC q2w add-on to background therapy; 468 randomized-and-treated; 469 in the safety population. Placebo: 465 randomized to placebo; 465 randomized-and-treated; 464 in the safety population (1 participant allocated to placebo but inadvertently dosed with dupilumab 300 mg q2w on day 113, so analyzed under the dupilumab arm for safety).	Serious adverse events. Outcome-specific timepoint NR; trial follow-up context: 52-week double-blind treatment period, within a total study duration of ~68 weeks (4-week screening + 52-week treatment + 12-week follow-up); primary/key secondary endpoints assessed to week 52 (FEV1 also assessed at week 12). Separately, AE/SAE/mortality data collection window extended to last dose of study treatment + 98 days (up to 506 days).	Safety - SAE, n/N per arm: placebo: 79/464 (17.0%); dupilumab: 65/469 (13.9%). Safety Population.	NR/not obtainable - no comparative effect with precision was present; see per-arm summary.	ClinicalTrials.gov NCT04456673	No - transcribed as reported; S1 adds no calculation.	
	Active: 470 randomized to dupilumab 300 mg SC q2w add-on to background therapy; 468 randomized-and-treated; 469 in the safety population. Placebo: 465 randomized to placebo; 465 randomized-and-treated; 464 in the safety population (1 participant allocated to placebo but inadvertently dosed with dupilumab 300 mg q2w on day 113, so analyzed under the dupilumab arm for safety)	AEs leading to discontinuation. Outcome-specific timepoint NR; trial follow-up context: 52-week double-blind treatment period, within a total study duration of ~68 weeks (4-week screening + 52-week treatment + 12-week follow-up); primary/key secondary endpoints assessed to week 52 (FEV1 also assessed at week 12). Separately, AE/SAE/mortality data collection window extended to last dose of study treatment + 98 days (up to 506 days).	Safety - discontinuation due to AE, n/N per arm: placebo: 7/465; dupilumab: 14/470 (denominator is randomized N, not safety population N). This is not a separately registered CT.Gov outcome measure - it is only recoverable from the participant-flow drop/withdrawal table.	NR/not obtainable - no comparative effect with precision was present; see per-arm summary.	ClinicalTrials.gov NCT04456673	No - transcribed as reported; S1 adds no calculation.	
	Active: 470 randomized to dupilumab 300 mg SC q2w add-on to background therapy; 468 randomized-and-treated; 469 in the safety population. Placebo: 465 randomized to placebo; 465 randomized-and-treated; 464 in the safety population (1 participant allocated to placebo but inadvertently dosed with dupilumab 300 mg q2w on day 113, so analyzed under the dupilumab arm for safety)	All-cause death. Outcome-specific timepoint NR; trial follow-up context: 52-week double-blind treatment period, within a total study duration of ~68 weeks (4-week screening + 52-week treatment + 12-week follow-up); primary/key secondary endpoints assessed to week 52 (FEV1 also assessed at week 12). Separately, AE/SAE/mortality data collection window extended to last dose of study treatment + 98 days (up to 506 days).	Safety - death, n/N per arm: placebo: 8/464; dupilumab: 14/469. Safety population. This is not a separately registered CT.Gov outcome measure - only recoverable from the adverse-events module’s group-level death counts.	NR/not obtainable - no comparative effect with precision was present; see per-arm summary.	ClinicalTrials.gov NCT04456673	No - transcribed as reported; S1 adds no calculation.	
	Active: 470 randomized to dupilumab 300 mg SC q2w add-on to background therapy; 468 randomized-and-treated; 469 in the safety population. Placebo: 465 randomized to placebo; 465 randomized-and-treated; 464 in the safety population (1 participant allocated to placebo but inadvertently dosed with dupilumab 300 mg q2w on day 113, so analyzed under the dupilumab arm for safety)	AEs of special interest. Outcome-specific timepoint NR; trial follow-up context: 52-week double-blind treatment period, within a total study duration of ~68 weeks (4-week screening + 52-week treatment + 12-week follow-up); primary/key secondary endpoints assessed to week 52 (FEV1 also assessed at week 12). Separately, AE/SAE/mortality data collection window extended to last dose of study treatment + 98 days (up to 506 days).	NR/not obtainable - no trial-specific numeric result for this domain was present in the extraction workbook.	NR/not obtainable - no trial-specific numeric result for this domain was present in the extraction workbook.	Extraction workbook (no matching trial-specific outcome row).	Not applicable - NR/not obtainable.	
S1.3 METREX (NCT02105948) [36]							
METREX	Active: STARTED: mepolizumab 100 mg - high stratum=233; mepolizumab 100 mg - low stratum=184 → arm total 233+184=417 (baseline denom confirms same; total=836). Placebo: STARTED: placebo - high stratum=229; placebo - low stratum=190 → arm total 229+190=419 (baseline denom confirms same; total=836)	Moderate/severe exacerbation rate. Outcome-specific timepoint NR; trial follow-up context: 52-week double-blind treatment plus 8-week safety follow-up (~62 weeks total incl. 1-2-week screening).	Exacerbation - adjusted annualized rate, active arm (mepolizumab 100 mg, eosinophilic-phenotype mITT high stratum, n=462): 1.40 exacerbations/year - mepolizumab 100 mg arm, METREX mITT eosinophilic-phenotype population (n=462). Exacerbation - adjusted annualized rate, active arm (overall mITT, n=836): (PRIMARY). Rate of moderate or severe exacerbations in the mITT population: mepolizumab 100 mg=1.49(1.35-1.64) (LS-mean per year). Exacerbation - adjusted annualized rate, placebo arm (eosinophilic-phenotype mITT high stratum, n=462): 1.71 exacerbations/year - mepolizumab 100 mg vs placebo, moderate/severe exacerbation rate, METREX mITT population with eosinophilic phenotype (n=462, stratified by blood eosinophil count ≥150/µL at screening or ≥300/µL in prior year). Exacerbation - adjusted annualized rate, placebo arm (overall mITT, n=836): (PRIMARY) Rate of Moderate or Severe Exacerbations in the mITT Population: placebo=1.52 (1.38-1.68) (LS-mean per year) (POOLED METREX+METREO, N=1136 - NOT METREX-only) Exacerbation adjusted annualized rate, mepolizumab 100 mg arm: 1.32 exacerbations/year (mepolizumab 100 mg arm); RR vs placebo 0.82 (95% CI: 0.71-0.95), p=0.006.	Eosinophil 150-<300 cells/µL - exacerbation RR vs placebo (95% CI), METREX mITT high stratum: RR 0.92 (95% CI: 0.73-1.16) - mepolizumab 100 mg vs placebo, moderate/severe exacerbation rate, baseline eosinophils 150-300 cells/µL, METREX mITT high-stratum cohort; reported as a sensitivity analysis (Table S9) in Bhatt et al. [35]. Eosinophil <150 cells/µL (screening, no ≥300 in prior year) - exacerbation outcome, METREX-specific: Not obtainable as a METREX-specific numeric rate/RR. The pooled METREX+METREO paper states only, qualitatively, that patients with screening eosinophils <150 cells/µL but a historical count ≥300 cells/µL in the prior year “exhibited the greatest reductions” in exacerbations and HRQoL - no numeric estimate is given, and METREX is not isolated from METREO in this statement (not obtainable).Eosinophil subgroup - categorical band-level exacerbation RR (150-<300/300-<500/≥500 cells/µL) (POOLED): Pooled METREX+METREO mepolizumab 100 mg vs placebo exacerbation RR (95% CI): <150 cells/µL 1.10 (0.91-1.34); 150-<300 0.92 (0.76-1.11); 300-<500 0.75 (0.55-1.00); ≥500 0.72 (0.48-1.09).Eosinophil subgroup - modelled exacerbation RR at 300 cells/µL (POOLED, N=1510): Predicted 14% reduction; RR 0.86 (95% CI: 0.75-0.99) at 300 cells/µL screening eosinophil threshold, eosinophil-response model across N=1510 pooled METREX+METREO patients. POOLED - not METREX-alone. Eosinophil subgroup - modelled exacerbation RR at 500 cells/µL (POOLED): Predicted 23% reduction; RR 0.77 (95% CI: 0.63-0.93) at 500 cells/µL. POOLED - not METREX-alone. Eosinophil subgroup - modelled exacerbation RR at 750 cells/µL (POOLED): Predicted 30% reduction; RR 0.70 (95% CI: 0.55-0.90) at 750 cells/µL. POOLED - not METREX-alone. Eosinophil subgroup - pooled aggregate exacerbation rate/RR, mepolizumab 100 mg vs placebo (METREX+METREO POOLED): N=1136 high-eosinophil mITT population pooled across METREX+METREO (mepolizumab 100 mg, N=456, mepolizumab 300 mg N=225, placebo N=455): annual moderate/severe exacerbation rate 1.32 (mepo100) vs 1.61 (placebo)/year; RR 0.82 (95% CI: 0.71-0.95), p=0.006. POOLED - not decomposable to METREX-alone from this source. Eosinophil subgroup - predicted exacerbation rate reduction for OCS-treated exacerbations by eosinophil count (METREX+METREO POOLED): eosinophil-response-model-predicted rate reductions for exacerbations requiring OCS treatment: 22% at 300 cells/µL, 35% at 500 cells/µL, and a third value printed in the source itself as “43% at 500 cells/µL” (an apparent duplicate label; most plausibly intended as ~750 cells/µL by analogy to the general-exacerbation model, but not confirmable from extracted text). Antibiotic-requiring exacerbations showed no clear eosinophil-count relationship. POOLED - not METREX-alone. Eosinophil subgroup - severe exacerbations (METREX+METREO POOLED): RR 0.88 (95% CI: 0.62-1.25), p=0.475 (not significant) for severe exacerbations, mepolizumab 100 mg vs placebo, POOLED METREX+METREO. Exacerbation - rate ratio vs placebo, 95% CI (eosinophilic-phenotype mITT subgroup, n=462): rate ratio: 0.82, 95% CI: 0.68-0.98, adjusted p=0.04 (statistically significant, co-primary endpoint) - confirmed verbatim. Exacerbation - rate ratio vs placebo, 95% CI (overall mITT, n=836): Rate ratio 0.98, 95% CI: 0.85-1.12, adjusted p>0.99 (not statistically significant) - confirmed verbatim (POOLED METREX+METREO - NOT METREX-only, POST HOC). Eosinophil-stratified predicted rate reduction for exacerbations requiring corticosteroids: predicted 22% at 300 cells/µL and 35% at 500 cells/µL; a third value printed verbatim in the source as “43% at 500 cells/µL” (likely intended as 750 cells/µL given the general-exacerbation model’s pattern, but unconfirmed). No RR/CI reported. Antibiotic-requiring exacerbations showed no clear eosinophil relationship (POOLED METREX+METREO - NOT METREX-only) Severe exacerbations - RR vs placebo: RR 0.88 (95% CI: 0.62-1.25), p=0.475 (POOLED METREX+METREO, N=1510 - NOT METREX-only). Eosinophil ≥300 cells/µL - predicted exacerbation RR vs placebo: predicted 14% reduction; RR: 0.86 (95% CI: 0.75-0.99) at 300 cells/µL. Same underlying fact as the pass1 single-pass row for this threshold - both pipelines independently and correctly extracted the identical published number (POOLED METREX+METREO, N=1510 - NOT METREX-only). Eosinophil ≥500 cells/µL - predicted exacerbation RR vs placebo: predicted 23% reduction; RR 0.77 (95% CI: 0.63-0.93) at 500 cells/µL (POOLED METREX+METREO, N=1510: NOT METREX-only). Eosinophil ≥750 cells/µL - predicted exacerbation RR vs placebo: predicted 30% reduction; RR: 0.70 (95% CI: 0.55-0.90) at 750 cells/µL.	PMC12992723; PMC8215850; PMID 28893134 abstractClinicalTrials.gov NCT02105948; PMID 28893134 abstract	No - transcribed as reported; S1 adds no calculation.	
	Active: STARTED: mepolizumab 100 mg - high stratum=233; mepolizumab 100 mg - low stratum=184 → arm total 233+184=417 (baseline denom confirms same; total=836). Placebo: STARTED: placebo - high stratum=229; placebo - low stratum=190 → arm total 229+190=419 (baseline denom confirms same; total=836)	Pre-bronchodilator FEV1. Outcome-specific timepoint NR; trial follow-up context: 52-week double-blind treatment plus 8-week safety follow-up (~62 weeks total incl. 1-2-week screening).	FEV1 - LS-mean change from baseline, active arm: not obtainable for METREX alone - same reason as placebo-arm FEV1 row (not obtainable). FEV1 - LS-mean change from baseline, placebo arm: not obtainable for METREX alone. Pooled METREX+METREO figure confirmed exact: pre-bronchodilator FEV1 differences of 5-31 mL across all timepoints between mepo 100 mg and placebo (not obtainable).	FEV1 - LS-mean difference vs placebo (mL), SE/95% CI: not obtainable for METREX alone. Pooled METREX+METREO exploratory figure confirmed exact (5-31 mL range across timepoints, no METREX-specific point estimate/CI) (not obtainable) (POOLED METREX+METREO - NOT METREX-only) FEV1 - LS-mean change, pre-bronchodilator, both arms: Between-group difference in pre-bronchodilator FEV1 ranged 5-31 mL across all timepoints; no single-timepoint LS-mean, SE, or CI reported in the source text.	not obtainable from any accessed source for METREX-alonePMC8215850	No - transcribed as reported; S1 adds no calculation.	
	Active: STARTED: mepolizumab 100 mg - high stratum=233; mepolizumab 100 mg - low stratum=184 → arm total 233+184=417 (baseline denom confirms same; total=836). Placebo: STARTED: placebo - high stratum=229; placebo - low stratum=190 → arm total 229+190=419 (baseline denom confirms same; total=836)	Post-bronchodilator FEV1. Outcome-specific timepoint NR; trial follow-up context: 52-week double-blind treatment plus 8-week safety follow-up (~62 weeks total incl. 1-2-week screening).	NR/not obtainable - no trial-specific numeric result for this domain was present in the extraction workbook.	NR/not obtainable: no trial-specific numeric result for this domain was present in the extraction workbook.	Extraction workbook (no matching trial-specific outcome row).	Not applicable - NR/not obtainable.	
	Active: STARTED: mepolizumab 100 mg - High stratum=233; mepolizumab 100 mg - low stratum=184 → arm total 233+184=417 (baseline denom confirms same; total=836). Placebo: STARTED: placebo - high stratum=229; placebo - low stratum=190 → arm total 229+190=419 (baseline denom confirms same; total=836)	SGRQ total score. week 52, week 12-1, week 24-3, week 52-2, week 52, week 12-1, week 24-3, week 52-2	SGRQ - LS-mean change from baseline, active arm: change from baseline in mean total SGRQ, mITT. Population: mepolizumab 100 mg=-3.2 (high stratum measure: mepolizumab 100 mg=-2.8). SGRQ - LS-mean change from baseline, placebo arm: change from baseline in mean total SGRQ, mITT population: placebo=-4.0 (high stratum measure: placebo=-3.0)	Eosinophil subgroup - SGRQ and CAT LS-mean difference vs placebo at week 52 (METREX+METREO POOLED): SGRQ total, mepolizumab 100 mg vs placebo, POOLED METREX+METREO: week 52 difference -0.7 (95% CI: -2.7 to 1.3, p=0.486, NS); week 12-1.5 (95% CI: -3.1 to 0.0); week 24-3.4 (95% CI: -5.2 to -1.6). SGRQ symptom domain week 52-2.9 (95% CI: -5.3 to -0.5, p=0.020). SGRQ responders (≥4pt), week 52: 42% (mepo) vs 32% (placebo), OR 1.23 (95% CI: 0.94-1.61, p=0.138). CAT week 52 difference -0.9 (95% CI: -1.8 to 0, p=0.039). CAT responders (≥2pt) week 52: 39% vs 33%, OR: 1.39 (95% CI: 1.04-1.85, p=0.024).SGRQ: LS-mean difference vs placebo, SE/95% CI: SGRQ mITT analysis: Mean difference (mepolizumab - placebo)=0.7 CI (-1.5 to 2.9) p>0.999 (adjusted), p=0.532 (nominal); high stratum analysis: Mean difference=0.2, CI: -2.8 to 3.2 (POOLED METREX+METREO -NOT METREX-only) SGRQ total score - LS-mean difference vs placebo at week 52: difference -0.7 (95% CI: -2.7 to 1.3), p=0.486 at week 52; week k12-1.5 (95% CI: -3.1,0.0); week 24-3.4 (95% CI: -5.2,-1.6); symptom-domain week 52-2.9 (95% CI: -5.3, -0.5) p=0.020; responders 42% mepo vs 32% placebo, OR 1.23 (95% CI: 0.94-1.61), p=0.138.	PMC8215850; ClinicalTrials.gov NCT02105948	No - transcribed as reported; S1 adds no calculation.	
	Active: STARTED: mepolizumab 100 mg - high stratum=233; mepolizumab 100 mg - low stratum=184 → arm total 233+184=417 (baseline denom confirms same; total=836). Placebo: STARTED: placebo - high stratum=229; placebo - low stratum=190 → arm total 229+190=419 (baseline denom confirms same; total=836).	E-RS:COPD. Outcome-specific timepoint NR; trial follow-up context: 52-week double-blind treatment plus 8-week safety follow-up (~62 weeks total incl. 1-2-week screening).	E-RS - LS-mean change from baseline, active arm: not obtainable/not applicable - same reason as placebo-arm E-RS row (not obtainable).E-RS - LS-mean change from baseline, placebo arm: not obtainable/not applicable - confirmed: the trial’s registered secondary outcomes list only SGRQ and CAT, no E-RS measure registered for METREX. No E-RS data in the NEJM abstract or PMC8215850 (not obtainable).	E-RS - LS-mean difference vs placebo, SE/95% CI: not obtainable/not applicable - same reason as above (not obtainable).	ClinicalTrials.gov NCT02105948	Not applicable - NR/not obtainable.	
	Active: STARTED: mepolizumab 100 mg - high stratum=233; mepolizumab 100 mg - low stratum=184 → arm total 233+184=417 (baseline denom confirms same; Total=836). Placebo: STARTED: placebo - high stratum=229; placebo - low stratum=190 → arm total 229+190=419 (baseline denom confirms same; total=836)	Time to first exacerbation218 days, 155 days	NR/not obtainable - no per-arm summary was present; see comparative/qualitative result.	Eosinophil subgroup - time to first moderate/severe exacerbation (METREX+METREO POOLED): Kaplan-Meier median: 218 days (mepolizumab 100 mg) vs 155 days (placebo); HR: 0.80, 95% CI: 0.68-0.94, p=0.006 - confirmed exact. POOLED, not METREX-alone.	PMC8215850	No - transcribed as reported; S1 adds no calculation.	
	Active: STARTED: mepolizumab 100 mg - high stratum=233; mepolizumab 100 mg - low stratum=184 → arm total 233+184=417 (baseline denom confirms same; total=836). Placebo: STARTED: placebo - high stratum=229; placebo - low stratum=190 → arm total 229+190=419 (baseline denom confirms same; total=836)	Rescue-medication use. Outcome-specific timepoint NR; trial follow-up context: 52-week double-blind treatment plus 8-week safety follow-up (~62 weeks total incl. 1-2-week screening).	NR/not obtainable - no trial-specific numeric result for this domain was present in the extraction workbook.	NR/not obtainable - no trial-specific numeric result for this domain was present in the extraction workbook.	Extraction workbook (no matching trial-specific outcome row).	Not applicable - NR/not obtainable.	
	Active: STARTED: mepolizumab 100 mg - high stratum=233; mepolizumab 100 mg - low stratum=184 → arm total 233+184=417 (baseline denom confirms same; total=836). Placebo: STARTED: placebo - high stratum=229; placebo - low stratum=190 → arm total 229+190=419 (baseline denom confirms same; total=836)	Any treatment-emergent adverse event. Outcome-specific timepoint NR; trial follow-up context: 52-week double-blind treatment plus 8-week safety follow-up (~62 weeks total incl. 1-2-week screening).	NR/not obtainable - no trial-specific numeric result for this domain was present in the extraction workbook.	NR/not obtainable - no trial-specific numeric result for this domain was present in the extraction workbook.	Extraction workbook (no matching trial-specific outcome row).	Not applicable - NR/not obtainable.	
	Active: STARTED: mepolizumab 100 mg - high stratum=233; mepolizumab 100 mg - low stratum=184 → arm total 233+184=417 (baseline denom confirms same; total=836). Placebo: STARTED: placebo - high stratum=229; placebo - low stratum=190 → arm total 229+190=419 (baseline denom confirms same; total=836)	Serious adverse events. Outcome-specific timepoint NR; trial follow-up context: 52-week double-blind treatment plus 8-week safety follow-up (~62 weeks total incl. 1-2-week screening).	Safety - SAE, n/N per arm: serious. Affected - placebo high=80/229, low=51/190 → 131/419; mepolizumab 100 mg, high=65/233, low=50/184 → 115/417	NR/not obtainable - no comparative effect with precision was present; see per-arm summary.	ClinicalTrials.gov NCT02105948	No - transcribed as reported; S1 adds no calculation.	
	Active: STARTED: mepolizumab 100 mg - high stratum=233; mepolizumab 100 mg - low stratum=184 → arm total 233+184=417 (baseline denom confirms same; total=836). Placebo: STARTED: placebo - high stratum=229; placebo - low stratum=190 → arm total 229+190=419 (baseline denom confirms same; total=836)	AEs leading to discontinuation. Outcome-specific timepoint NR; trial follow-up context: 52-week double-blind treatment plus 8-week safety follow-up (~62 weeks total incl. 1-2-week screening).	Safety - discontinuation due to AE, n/N per arm: withdrew IP due to adverse event - placebo high=20, low=15 → 35/419; mepolizumab 100 mg, high=16, low=13 → 29/417	NR/not obtainable - no comparative effect with precision was present; see per-arm summary.	ClinicalTrials.gov NCT02105948	No - transcribed as reported; S1 adds no calculation.	
	Active: STARTED: mepolizumab 100 mg - high stratum=233; mepolizumab 100 mg - low stratum=184 → arm total 233+184=417 (baseline denom confirms same; total=836). Placebo: STARTED: placebo - high stratum=229; placebo - low stratum=190 → arm total 229+190=419 (baseline denom confirms same; total=836)	All-cause death. Outcome-specific timepoint NR; trial follow-up context: 52-week double-blind treatment plus 8-week safety follow-up (~62 weeks total incl. 1-2-week screening).	Safety - death, n/N per arm: deaths - placebo high=8/229, low=9/190 → 17/419; mepolizumab 100 mg high=6/233, low=10/184 → 16/417	NR/not obtainable - no comparative effect with precision was present; see per-arm summary.	ClinicalTrials.gov NCT02105948	No - transcribed as reported; S1 adds no calculation.	
	Active: STARTED: mepolizumab 100 mg - High stratum=233; mepolizumab 100 mg - low stratum=184 → arm total 233+184=417 (baseline denom confirms same; total=836). Placebo: STARTED: placebo - high stratum=229; placebo - low stratum=190 → arm total 229+190=419 (baseline denom confirms same; total=836)	AEs of special interest. Outcome-specific timepoint NR; trial follow-up context: 52-week double-blind treatment plus 8-week safety follow-up (~62 weeks total incl. 1-2-week screening).	NR/not obtainable - no trial-specific numeric result for this domain was present in the extraction workbook.	NR/not obtainable - no trial-specific numeric result for this domain was present in the extraction workbook.	Extraction workbook (no matching trial-specific outcome row).	Not applicable - NR/not obtainable.	
S1.4 METREO (NCT02105961) [36]							
METREO	Active: mepolizumab 100 mg SC=223; mepolizumab 300 mg SC=225 (STARTED: placebo=226; mepolizumab 100 mg SC=223; mepolizumab 300 mg SC=225). Active: mepolizumab 100 mg SC=223; mepolizumab 300 mg SC=225Placebo: placebo=226 (STARTED: placebo=226; baseline denom. Placebo=226).	Moderate/severe exacerbation rate. Outcome-specific timepoint NR; trial follow-up context: 52-week randomized treatment period, preceded by 1-2-week screening and followed by 8-week (safety) follow-up (total participation ~62 weeks)	Exacerbation - adjusted annualized rate, active arm 300 mg: 1.27 exacerbations/year (moderate/severe, mITT). Exacerbation - adjusted annualized rate, active arm(s): mepolizumab 100 mg: 1.19 exacerbations/year; mepolizumab 300 mg: 1.27 exacerbations/year (moderate/severe, mITT population); placebo: 1.49/year. Exacerbation - adjusted annualized rate, placebo arm: 1.49 exacerbations/year (moderate/severe, mITT)	Eosinophil-stratified exacerbation RR by screening count - pooled METREX+METREO predictive model (mepo 100mg vs placebo), NOT METREO-isolated: Predictive fractional-polynomial model (pooled METREX+METREO, N=1510, mepolizumab 100 mg vs placebo, NOT METREO-isolated): at 300 cells/µL, RR: 0.86 (95% CI: 0.75-0.99), 14% reduction; at 500 cells/µL, RR: 0.77 (95% CI: 0.63-0.93), 23% reduction; at 750 cells/µL, RR: 0.70 (95% CI: 0.55-0.90), 30% reduction. Post hoc, for corticosteroid-requiring exacerbations: 22% reduction at 300 cells/µL, 35% at 500 cells/µL, and 43% at a threshold the source literally repeats as “500 cells/µL” - a genuine duplication/error present in the published article itself, plausibly (but not verifiably) intended as 750 cells/µL. Eosinophil-subgroup - predicted reduction in corticosteroid-requiring exacerbations by screening BEC (pooled METREX+METREO, post hoc): Predicted rate reductions with mepolizumab 100 mg vs placebo: 22% at BEC=300 cells/µL, 35% at BEC=500 cells/µL, and 43% at a third threshold the source article literally repeats as “500 cells/µL” - confirmed genuinely present verbatim in the article text, not an extraction artifact; plausibly (not verifiably) intended as 750 cells/µL by analogy with the exacerbation-rate model. No CI given for any of the three figures. Same pooled N=1510 METREX+METREO model. Exacerbation - rate ratio vs placebo, 95% CI: mepolizumab 100 mg vs placebo: RR 0.80 (95% CI: 0.65-0.98), adjusted p=0.07. mepolizumab 300 mg vs placebo: RR 0.86 (95% CI: 0.70-1.05), adjusted p=0.14. Neither comparison reached statistical significance after pre-specified multiplicity correction. Exacerbation - rate ratio vs placebo, 95% CI (300 mg): Rate ratio 0.86 (95% CI: 0.70-1.05) vs placebo; adjusted p=0.14 - not statistically significant.	PMC8215850; PMID 28893134, PMID 28893134	No - transcribed as reported; S1 adds no calculation.	
	Active: mepolizumab 100 mg SC=223; mepolizumab 300 mg SC=225 (STARTED: placebo=226; mepolizumab 100 mg SC=223; mepolizumab 300 mg SC=225). Active: mepolizumab 100 mg SC=223; mepolizumab 300 mg SC=225. Placebo: placebo=226 (STARTED: placebo=226; baseline denom. Placebo=226)	Pre-bronchodilator FEV1. Outcome-specific timepoint NR; trial follow-up context: 52-week randomized treatment period, preceded by 1-2-week screening and followed by 8-week (safety) follow-up (total participation ~62 weeks).	FEV1 - LS-mean change from baseline, active arm(s): not obtainable - same reasoning as placebo-arm FEV1 row (not obtainable). FEV1- LS-mean change from baseline, placebo arm: Not obtainable - FEV1 does not appear among CT.Gov’s outcome measures for this trial (confirmed: only 1 primary (exacerbation rate) and 4 secondary outcomes (time to first exacerbation, ED/hosp exacerbation rate, SGRQ change, CAT change) were returned, no FEV1); NEJM abstract gives no FEV1 data (not obtainable).	FEV1 - LS-mean difference vs placebo (mL), SE/95% CI: not obtainable for METREO alone - CT.Gov did not return FEV1 as an outcome measure; PMC8215850 gives only a pooled METREX+METREO 5-31 mL range across timepoints, no SE/CI, not METREO-isolated (not obtainable).	ClinicalTrials.gov NCT02105961; PMC8215850	Not applicable - NR/not obtainable.	
	Active: mepolizumab 100 mg SC=223; mepolizumab 300 mg SC=225 (STARTED: placebo=226; mepolizumab 100 mg SC=223; mepolizumab 300 mg SC=225)Active: mepolizumab 100 mg SC=223; mepolizumab 300 mg SC=225Placebo: placebo=226 (STARTED: placebo=226; baseline denom. Placebo=226)	Post-bronchodilator FEV1. Outcome-specific timepoint NR; trial follow-up context: 52-week randomized treatment period, preceded by 1-2-week screening, followed by 8-week (safety) follow-up (total participation ~62 weeks)	NR/not obtainable - no trial-specific numeric result for this domain was present in the extraction workbook.	NR/not obtainable - no trial-specific numeric result for this domain was present in the extraction workbook.	Extraction workbook (no matching trial-specific outcome row).	Not applicable - NR/not obtainable.	
	Active: mepolizumab 100 mg SC=223; mepolizumab 300 mg SC=225 (STARTED: placebo=226; mepolizumab 100 mg SC=223; mepolizumab 300 mg SC=225)Active: mepolizumab 100 mg SC=223; mepolizumab 300 mg SC=225Placebo: placebo=226 (STARTED: placebo=226; baseline denom. Placebo=226)	SGRQ total score. Outcome-specific timepoint NR; trial follow-up context: 52-week randomized treatment period, preceded by 1-2- week screening and followed by 8-week (safety) follow-up (total participation ~62 weeks)	SGRQ - LS-mean change from baseline, active arm(s): mepolizumab 100 mg SC=-5.0; mepolizumab 300 mg SC=-3.3 (SGRQ change: placebo=-3.1; mepolizumab 100 mg SC=-5.0; mepolizumab 300 mg SC=-3.3). SGRQ - LS-mean change from baseline, placebo arm: placebo=-3.1.	SGRQ - LS-mean difference vs placebo, SE/95% CI: mean difference (mepolizumab 100 - placebo)=-1.8 CI: -4.5, 0.8; mean difference (mepolizumab 300 - placebo)=-0.1 CI: -2.8, 2.6.	ClinicalTrials.gov NCT02105961	No - transcribed as reported; S1 adds no calculation.	
	Active: mepolizumab 100 mg SC=223; mepolizumab 300 mg SC=225 (STARTED: placebo=226; mepolizumab 100 mg SC=223; mepolizumab 300 mg SC=225). Active: mepolizumab 100 mg SC=223; mepolizumab 300 mg SC=225. Placebo: placebo=226 (STARTED: placebo=226; baseline denom. Placebo=226)	E-RS:COPD. Outcome-specific timepoint NR; trial follow-up context: 52-week randomized treatment period, preceded by 1-2-week screening and followed by 8-week (safety) follow-up (total participation ~62 weeks)	E-RS - LS-mean change from baseline, active arm(s): Not obtainable - E-RS not a specified/registered endpoint for this trial (not obtainable). E-RS - LS-mean change from baseline, placebo arm: Not obtainable - E-RS was not among the outcome measures CT.Gov returned for this trial; the trial used CAT and SGRQ instead (secondary outcomes = time-to-exacerbation, ED/hosp rate, SGRQ, CAT - no E-RS) (not obtainable).	E-RS - LS-mean difference vs placebo, SE/95% CI: Not obtainable - E-RS not a specified/registered endpoint for this trial (not obtainable).	ClinicalTrials.gov NCT02105961	Not applicable - NR/not obtainable.	
	Active: mepolizumab 100 mg SC=223; mepolizumab 300 mg SC=225 (STARTED: placebo=226; mepolizumab 100 mg SC=223; mepolizumab 300 mg SC=225). Active: mepolizumab 100 mg SC=223; mepolizumab 300 mg SC=225. Placebo: placebo=226 (STARTED: placebo=226; baseline denom. Placebo=226)	Time to first exacerbation. Outcome-specific timepoint NR; trial follow-up context: 52-week randomized treatment period, preceded by 1-2-week screening and followed by 8-week (safety) follow-up (total participation ~62 weeks).	NR/not obtainable - no trial-specific numeric result for this domain was present in the extraction workbook.	NR/not obtainable - no trial-specific numeric result for this domain was present in the extraction workbook.	Extraction workbook (no matching trial-specific outcome row).	Not applicable - NR/not obtainable.	
	Active: mepolizumab 100 mg SC=223; mepolizumab 300 mg SC=225 (STARTED: placebo=226; mepolizumab 100 mg SC=223; mepolizumab 300 mg SC=225). Active: mepolizumab 100 mg SC=223; mepolizumab 300 mg SC=225. Placebo: placebo=226 (STARTED: placebo=226; baseline denom. Placebo=226).	Rescue-medication use. Outcome-specific timepoint NR; trial follow-up context: 52-week randomized treatment period, preceded by 1-2-week screening and followed by 8-week (safety) follow-up (total participation ~62 weeks).	NR/not obtainable - no trial-specific numeric result for this domain was present in the extraction workbook.	NR/not obtainable - no trial-specific numeric result for this domain was present in the extraction workbook.	Extraction workbook (no matching trial-specific outcome row).	Not applicable - NR/not obtainable.	
	Active: mepolizumab 100 mg SC=223; mepolizumab 300 mg SC=225 (STARTED: placebo=226; mepolizumab 100 mg SC=223; mepolizumab 300 mg SC=225). Active: mepolizumab 100 mg SC=223; mepolizumab 300 mg SC=225. Placebo: placebo=226 (STARTED: placebo=226; baseline denom. Placebo=226).	Any treatment-emergent adverse event. Outcome-specific timepoint NR; trial follow-up context: 52-week randomized treatment period, preceded by 1-2-week screening and followed by 8-week (safety) follow-up (total participation ~62 weeks).	NR/not obtainable - no trial-specific numeric result for this domain was present in the extraction workbook.	NR/not obtainable - no trial-specific numeric result for this domain was present in the extraction workbook.	Extraction workbook (no matching trial-specific outcome row).	Not applicable - NR/not obtainable.	
	Active: mepolizumab 100 mg SC=223; mepolizumab 300 mg SC=225 (STARTED: placebo=226; mepolizumab 100 mg SC=223; mepolizumab 300 mg SC=225). Active: mepolizumab 100 mg SC=223; mepolizumab 300 mg SC=225. Placebo: placebo=226 (STARTED: placebo=226; baseline denom. Placebo=226).	Serious adverse events. Outcome-specific timepoint NR; trial follow-up context: 52-week randomized treatment period, preceded by 1-2-week screening and followed by 8-week (safety) follow-up (total participation ~62 weeks)	Safety - SAE, n/N per arm: placebo serious. Affected=68/226; mepolizumab 100 mg SC serious. Affected=57/223; mepolizumab 300 mg SC serious. Affected=60/225	NR/not obtainable - no comparative effect with precision was present; see per-arm summary.	ClinicalTrials.gov NCT02105961	No - transcribed as reported; S1 adds no calculation.	
	Active: mepolizumab 100 mg SC=223; mepolizumab 300 mg SC=225 (STARTED: placebo=226; mepolizumab 100 mg SC=223; mepolizumab 300 mg SC=225). Active: mepolizumab 100 mg SC=223; mepolizumab 300 mg SC=225. Placebo: placebo=226 (STARTED: placebo=226; baseline denom. Placebo=226)	AEs leading to discontinuation. Outcome-specific timepoint NR; trial follow-up context: 52-week randomized treatment period, preceded by 1-2-week screening and followed by 8-week (safety) follow-up (total participation ~62 weeks).	Safety - discontinuation due to AE, n/N per arm: withdrew IP due to adverse event: placebo=27/226; mepolizumab 100 mg SC=9/223; mepolizumab 300 mg SC=25/225.	NR/not obtainable - no comparative effect with precision was present; see per-arm summary.	ClinicalTrials.gov NCT02105961	No - transcribed as reported; S1 adds no calculation.	
	Active: mepolizumab 100 mg SC=223; mepolizumab 300 mg SC=225 (STARTED: placebo=226; mepolizumab 100 mg SC=223; mepolizumab 300 mg SC=225)Active: mepolizumab 100 mg SC=223; mepolizumab 300 mg SC=225. Placebo: placebo=226 (STARTED: placebo=226; baseline denom. Placebo=226).	All-cause death. Outcome-specific timepoint NR; trial follow-up context: 52-week randomized treatment period, preceded by 1-2-week screening and followed by 8-week (safety) follow-up (total participation ~62 weeks).	Safety - death, n/N per arm: placebo deaths=9/226; mepolizumab 100 mg SC deaths=4/223; mepolizumab 300 mg SC deaths=8/225.	NR/not obtainable - no comparative effect with precision was present; see per-arm summary.	ClinicalTrials.gov NCT02105961	No - transcribed as reported; S1 adds no calculation.	
	Active: mepolizumab 100 mg SC=223; mepolizumab 300 mg SC=225 (STARTED: placebo=226; mepolizumab 100 mg SC=223; mepolizumab 300 mg SC=225). Active: mepolizumab 100 mg SC=223; mepolizumab 300 mg SC=225. Placebo: placebo=226 (STARTED: placebo=226; baseline denom. Placebo=226).	AEs of special interest. Outcome-specific timepoint NR; trial follow-up context: 52-week randomized treatment period, preceded by 1-2-week screening and followed by 8-week (safety) follow-up (total participation ~62 weeks).	NR/not obtainable - no trial-specific numeric result for this domain was present in the extraction workbook.	NR/not obtainable - no trial-specific numeric result for this domain was present in the extraction workbook.	Extraction workbook (no matching trial-specific outcome row).	Not applicable - NR/not obtainable.	
S1.5 MATINEE (NCT04133909) [17]							
MATINEE	Active: 403 (mepolizumab 100 mg SC q4w). Placebo: 401 (of 804 total randomized: 403 mepolizumab + 401 placebo).	Moderate/severe exacerbation rate. week 104, 52-week, 52 weeks, 104 weeks.	Exacerbation - adjusted annualized rate, active arm(s): mepolizumab 100 mg: adjusted annualized moderate/severe exacerbation rate 0.80/year (95% CI: 0.70-0.91), up to week 104. Exacerbation - adjusted annualized rate, placebo arm: placebo: adjusted annualized moderate/severe exacerbation rate 1.01/year (95% CI: 0.89-1.15), up to week 104.	Eosinophil 150-300 cells/µL subgroup - MATINEE-specific exacerbation reporting status: MATINEE’s enrollment required essentially all participants to have BEC ≥300 cells/µL at screening (plus a ≥150 cells/µL historical requirement), so there is no <300 cells/µL comparator within MATINEE itself, and a secondary ITC source explicitly confirms MATINEE did not report exacerbation rates stratified by the 150-300 cells/µL band (that analysis is only available for BOREAS/NOTUS/METREX). Eosinophil <150 cells/µL - exacerbation benefit (POOLED METREX+METREO+MATINEE, NOT MATINEE-specific): Patients with persistently low BEC (<150 cells/µL) experienced no exacerbation benefit with mepolizumab. Qualitative statement only, no numeric rate ratio/CI, pooled across three trials, not broken out by individual trial. Eosinophil ≥150 cells/µL (elevated-BEC subgroup) - ED-visit/hospitalization exacerbation benefit (POOLED METREX+METREO+MATINEE, NOT MATINEE-specific): Across various subgroups with elevated BEC, starting from ≥150 cells/µL, mepolizumab reduced exacerbations requiring an ED visit and/or hospitalization. Qualitative statement only, no numeric RR/CI given, pooled across three trials, not MATINEE-specific. Eosinophil ≥300 cells/µL at any pre-randomization timepoint - exacerbation reduction (POOLED METREX+METREO+MATINEE, NOT MATINEE-specific): ~21% reduction in annualized moderate/severe exacerbation rate vs placebo in patients with BEC ≥300 cells/µL at any pre-randomization timepoint; 12-27% reduction in patients with persistently elevated BEC; 22-36% reduction in patients with variable BEC over time. Pooled across METREX+METREO+MATINEE, not MATINEE-specific; no numeric 95% CI given in the abstract. Exacerbation - rate ratio vs placebo, 95% CI (52-week fixed-duration subgroup only, NOT full randomized cohort): Rate ratio 0.85 (95% CI> 0.62-1.16) in the 52-week fixed-duration subgroup (pre-amendment enrollees) - more modest than, and not directly comparable to, the full-cohort primary result (RR 0.79). This figure is only reported in the secondary ITC publication, not in the primary NEJM trial report. Exacerbation - rate ratio vs placebo, 95% CI (primary endpoint, full randomized cohort): Rate ratio 0.79 (95% CI> 0.66-0.94), p=0.01, full variable-duration cohort up to week 104 (primary endpoint). Secondary ITC-paper framing: mepolizumab reduced exacerbations by ~15% over 52 weeks and ~25% over 104 weeks (total reduction 18%) vs placebo.	ClinicalTrials.gov NCT04133909; PMC12992723; PMID 42265988; DOI 10.1093/ajrccm/aamag288; PMID 40305712	No - transcribed as reported; S1 adds no calculation.	
	Active: 403 (mepolizumab 100 mg SC q4w). Placebo: 401 (of 804 total randomized: 403 mepolizumab + 401 placebo)	Pre-bronchodilator FEV152-week	FEV1 - LS-mean change from baseline, active arm(s): Not obtainable - same limitation as placebo-arm FEV1 row (not obtainable). FEV1 - LS-mean change from baseline, placebo arm: Not obtainable. The raw registry contains a posted results section, but no FEV1-change outcome for MATINEE; the accessible publication abstract also supplies no per-arm placebo FEV1 change (not obtainable).	FEV1 - LS-mean difference vs placebo (mL), SE/95% CI: not obtainable for MATINEE-alone. Confirmed the 83.4 mL (95% CI: 36.1130.7) figure in PMC12992723 is explicitly the dupilumab-vs-mepolizumab ITC contrast (using pooled MATINEE 52-week-fixed + METREX mITT-high-stratum data), NOT a MATINEE-alone mepolizumab-vs-placebo estimate (not obtainable).	ClinicalTrials.gov NCT04133909; PMC12992723	Not applicable - NR/not obtainable.	
	Active: 403 (mepolizumab 100 mg SC q4w). Placebo: 401 (of 804 total randomized: 403 mepolizumab + 401 placebo).	Post-bronchodilator FEV1. Outcome-specific timepoint NR; trial follow-up context: Variable: 52-104 weeks. Originally designed as a 52-week study; amended mid-trial (due to unexpectedly low exacerbation rates during the COVID-19 pandemic) to extend to 104 weeks and increase sample size. Patients enrolled before the amendment could self-select the extension; patients enrolled afterward were automatically assigned the 104-week duration. Trial start date 2019-10-30; primary completion and completion dates both 2024-08-08.	NR/not obtainable - no trial-specific numeric result for this domain was present in the extraction workbook.	NR/not obtainable - no trial-specific numeric result for this domain was present in the extraction workbook.	Extraction workbook (no matching trial-specific outcome row).	Not applicable - NR/not obtainable.	
	Active: 403 (mepolizumab 100 mg SC q4w). Placebo: 401 (of 804 total randomized: 403 mepolizumab + 401 placebo)	SGRQ total score52-week, week 52	SGRQ - LS-mean change from baseline, active arm(s): not obtainable - same limitation as placebo-arm SGRQ row (not obtainable). SGRQ - LS-mean change from baseline, placebo arm: Not obtainable as a continuous LS-mean change. The CT.Gov-registered SGRQ-C outcome is a dichotomized “≥4-point responder” percentage (see separate row), not a continuous LS-mean change score - confirmed via re-read of trial.secondary_outcomes (not obtainable).	SGRQ - LS-mean difference vs placebo, SE/95% CI: not obtainable for MATINEE-alone. Confirmed OR 1.16 (95% CI: 0.86-1.56) in PMC12992723 is explicitly the dupilumab-vs-mepolizumab ITC odds ratio for proportion achieving ≥4-point SGRQ improvement (pooled MATINEE 52-week-fixed + METREX mITT-high-stratum), NOT a MATINEE-alone placebo-controlled estimate (not obtainable). SGRQ-C ≥4-point responders at week 52, % (OR vs placebo) (secondary outcome): (SECONDARY), percentage of St. George’s Respiratory Questionnaire for COPD (SGRQ) Total Score Responders With >=4 Point Reduction From Baseline at week 52: mepolizumab 100 mg=50; placebo=46; ANALYSIS: odds ratio (OR)=1.17 CI: 0.87-1.57, p=0.291	ClinicalTrials.gov NCT04133909; PMC12992723	No - transcribed as reported; S1 adds no calculation.	
	Active: 403 (mepolizumab 100 mg SC q4w). Placebo: 401 (of 804 total randomized: 403 mepolizumab + 401 placebo)	E-RS:COPD. Weeks 49-52	E-RS - LS-mean change from baseline, active arm(s): not obtainable - same limitation as placebo-arm E-RS row (not obtainable). E-RS - LS-mean change from baseline, placebo arm: Not obtainable. The CT.Gov-registered E-RS: COPD outcome is a dichotomized “≥2-point responded” percentage (weeks 49-52 vs baseline), not a continuous LS-mean change score - confirmed via re-read of trial.secondary_outcomes (not obtainable).	E-RS - LS-mean difference vs placebo, SE/95% CI: not obtainable for MATINEE-alone. Confirmed OR: 1.76 (95% CI: 1.20-2.54) in PMC12992723 is explicitly the dupilumab-vs-mepolizumab ITC odds ratio for E-RS:COPD responder proportion, using pooled BOREAS+NOTUS vs MATINEE’s 52-week fixed cohort only (E-RS:COPD was not evaluated in METREX) - NOT a MATINEE-alone placebo-controlled estimate (not obtainable).E-RS:COPD ≥2-point responders (weeks 49-52), % (OR vs placebo) (secondary outcome): (SECONDARY). Percentage of Evaluating Respiratory Symptoms in COPD (E-RS: COPD) Responders With >=2 Point Reduction From Baseline: mepolizumab 100 mg=31; placebo=34; ANALYSIS: Odds Ratio (OR)=0.82, CI: 0.60-1.12 p=0.209	ClinicalTrials.gov NCT04133909; PMC12992723	No - transcribed as reported; S1 adds no calculation.	
	Active: 403 (mepolizumab 100 mg SC q4w). Placebo: 401 (of 804 total randomized: 403 mepolizumab + 401 placebo)	Time to first exacerbation: 419 days, 321 days	NR/not obtainable: no per-arm summary was present; see comparative/qualitative result.	Time to first moderate/severe exacerbation: HR vs placebo, 95% CI (secondary outcome): HR 0.77 (95% CI: 0.64-0.93), P=0.009; Kaplan-Meier median time to first exacerbation: mepolizumab 419 days vs placebo 321 days: CONFIRMED verbatim against primary source.	PMID 40305712	No: transcribed as reported; S1 adds no calculation.	
	Active: 403 (mepolizumab 100 mg SC q4w). Placebo: 401 (of 804 total randomized: 403 mepolizumab + 401 placebo)	Rescue-medication use. Outcome-specific timepoint NR; trial follow-up context: Variable 52-104 weeks. Originally designed as a 52-week study; amended mid-trial (due to unexpectedly low exacerbation rates during the COVID-19 pandemic) to extend to 104 weeks and increase sample size. Patients enrolled before the amendment could self-select the extension; patients enrolled afterward were automatically assigned the 104-week duration. Trial start date 2019-10-30; primary completion and completion dates both 2024-08-08.	NR/not obtainable: no trial-specific numeric result for this domain was present in the extraction workbook.	NR/not obtainable: no trial-specific numeric result for this domain was present in the extraction workbook.	Extraction workbook (no matching trial-specific outcome row).	Not applicable: NR/not obtainable.	
	Active: 403 (mepolizumab 100 mg SC q4w). Placebo: 401 (of 804 total randomized: 403 mepolizumab + 401 placebo)	Any treatment-emergent adverse event. Outcome-specific timepoint NR; trial follow-up context: Variable 52-104 weeks. Originally designed as a 52-week study; amended mid-trial (due to unexpectedly low exacerbation rates during the COVID-19 pandemic) to extend to 104 weeks and increase sample size. Patients enrolled before the amendment could self-select the extension; patients enrolled afterward were automatically assigned the 104-week duration. Trial start date 2019-10-30; primary completion and completion dates both 2024-08-08.	Safety: TEAE (combined any-AE), n/N per arm: Aggregate any-TEAE n/N remains not obtainable. The raw registry contains an adverse-events module with a 3% reporting threshold and posts participants with threshold-reported other/non-serious events: mepolizumab 195/403 and placebo 182/401. Those threshold-reported counts are not equivalent to any-TEAE incidence; the accessible publication text is qualitative (not obtainable).	NR/not obtainable: no comparative effect with precision was present; see per-arm summary.	ClinicalTrials.gov NCT04133909; PMC12992723	Not applicable: NR/not obtainable.	
	Active: 403 (mepolizumab 100 mg SC q4w). Placebo: 401 (of 804 total randomized: 403 mepolizumab + 401 placebo)	Serious adverse events. Outcome-specific timepoint NR; trial follow-up context: Variable 52-104 weeks. Originally designed as a 52-week study; amended mid-trial (due to unexpectedly low exacerbation rates during the COVID-19 pandemic) to extend to 104 weeks and increase sample size. Patients enrolled before the amendment could self-select the extension; patients enrolled afterward were automatically assigned the 104-week duration. Trial start date 2019-10-30; primary completion and completion dates both 2024-08-08.	Safety: SAE, n/N per arm: mepolizumab 100 mg: serious. Affected=101/403; placebo: serious. Affected=115/401	NR/not obtainable: no comparative effect with precision was present; see per-arm summary.	ClinicalTrials.gov NCT04133909	No: transcribed as reported; S1 adds no calculation.	
	Active: 403 (mepolizumab 100 mg SC q4w). Placebo: 401 (of 804 total randomized: 403 mepolizumab + 401 placebo)	AEs leading to discontinuation. Outcome-specific timepoint NR; trial follow-up context: variable 52-104 weeks. Originally designed as a 52-week study; amended mid-trial (due to unexpectedly low exacerbation rates during the COVID-19 pandemic) to extend to 104 weeks and increase sample size. Patients enrolled before the amendment could self-select the extension; patients enrolled afterward were automatically assigned the 104-week duration. Trial start date: 2019-10-30; primary completion and completion dates both 2024-08-08.	Safety: discontinuation due to AE, n/N per arm: mepolizumab 4/404 started; placebo 5/402 started; withdrew because of an adverse event.	NR/not obtainable: no comparative effect with precision was present; see per-arm summary.	ClinicalTrials.gov NCT04133909	No: transcribed as reported; S1 adds no calculation.	
	Active: 403 (mepolizumab 100 mg SC q4w). Placebo: 401 (of 804 total randomized: 403 mepolizumab + 401 placebo)	All-cause death. Outcome-specific timepoint NR; trial follow-up context: Variable 52-104 weeks. Originally designed as a 52-week study; amended mid-trial (due to unexpectedly low exacerbation rates during the COVID-19 pandemic) to extend to 104 weeks and increase sample size. Patients enrolled before the amendment could self-select the extension; patients enrolled afterward were automatically assigned the 104-week duration. Trial start date 2019-10-30; primary completion and completion dates both 2024-08-08.	Safety: death, n/N per arm: mepolizumab 100 mg: deaths=11/403; placebo: deaths=11/401	NR/not obtainable: no comparative effect with precision was present; see per-arm summary.	ClinicalTrials.gov NCT04133909	No: transcribed as reported; S1 adds no calculation.	
	Active: 403 (mepolizumab 100 mg SC q4w). Placebo: 401 (of 804 total randomized: 403 mepolizumab + 401 placebo)	AEs of special interest. Outcome-specific timepoint NR; trial follow-up context: variable 52-104 weeks. Originally designed as a 52-week study; amended mid-trial (due to unexpectedly low exacerbation rates during the COVID-19 pandemic) to extend to 104 weeks and increase sample size. Patients enrolled before the amendment could self-select the extension; patients enrolled afterward were automatically assigned the 104-week duration. Trial start date 2019-10-30; primary completion and completion dates both 2024-08-08.	NR/not obtainable: no trial-specific numeric result for this domain was present in the extraction workbook.	NR/not obtainable: no trial-specific numeric result for this domain was present in the extraction workbook.	Extraction workbook (no matching trial-specific outcome row).	Not applicable: NR/not obtainable.	
S1.6 GALATHEA (NCT02138916)							
GALATHEA	Active: STARTED: benralizumab 30 mg=554; benralizumab 100 mg=552 (baseline denom confirms 554 and 552). Placebo: STARTED: placebo=550 (baseline denom confirms placebo=550)	Moderate/severe exacerbation rate30 days, 90 days, week 56	Exacerbation: adjusted annualized rate, active arm(s): benralizumab 30 mg: 1.19/year (95% CI: 1.04-1.36); benralizumab 100 mg: 1.03/year (95% CI: 0.90-1.19): GALATHEA, EOS≥220/µL population, week 56. Exacerbation: adjusted annualized rate, placebo arm: 1.24 exacerbations/year (95% CI: 1.08-1.42): GALATHEA, EOS≥220/µL primary-endpoint analysis population, week 56.	Author attribution for the pooled recurrent-exacerbation post hoc analysis (PMID 37533773 / PMC10390712 / DOI 10.2147/COPD.S418944): Singh D, Criner GJ, Agustí A, Bafadhel M, Söderström J, Luporini Saraiva G, Song Y, Licaj I, Jison M, Martin UJ, Psallidas I. Benralizumab Prevents Recurrent Exacerbations in Patients with Chronic Obstructive Pulmonary Disease: A Post Hoc Analysis. Int J Chron Obstruct Pulmon Dis. 2023;18:1595-1599.Eosinophil <220/µL subgroup: exacerbation rate/rate ratio vs placebo: Annual COPD Exacerbation Rate (EOS<220/uL): benralizumab 30 mg=1.4(1.19-1.64); benralizumab 100 mg=1.32(1.12-1.56); placebo=1.30(1.11-1.52); RR 30mg vs placebo=1.07, CI: 0.86-1.34) p=0.5236; RR 100mg vs placebo=1.02, CI: 0.82-1.27) p=0.8812Eosinophil ≥220/µL subgroup: combined criteria (≥3 exacerbations/yr + triple therapy) RR vs placebo (POOLED GALATHEA+TERRANOVA, not GALATHEA-only): CAUTION: pooled across GALATHEA+TERRANOVA, not separable to GALATHEA alone. EOS≥220/µL + ≥3 prior-year exacerbations + triple therapy: benralizumab 100 mg RR 0.70 (95% CI: 0.56-0.88); 30 mg RR 0.99 (95% CI: 0.79-1.23) vs placebo. Eosinophil ≥300/µL responder subgroup: recurrent SEVERE exacerbation RR, 30-day and 90-day windows, benralizumab 100 mg vs placebo (not statistically significant) (POOLED GALATHEA+TERRANOVA, n=145): Same responder subpopulation (n=145). 30-day window: 3 events (benralizumab) vs 17 events (placebo), RR 0.33 (95% CI: 0.08-1.30; P=0.1133): not significant. 90-day window: 8 events (benralizumab) vs 24 events (placebo), RR 0.48 (95% CI: 0.16-1.43; P=0.1862): not significant. Eosinophil ≥300/µL responder subgroup: recurrent exacerbation RR, 30-day window, benralizumab 100 mg vs placebo (POOLED GALATHEA+TERRANOVA, n=145): Post hoc, pooled GALATHEA+TERRANOVA, benralizumab 100 mg only. Subpopulation: bEOS≥300 cells/µL + ≥3 prior-year exacerbations + triple therapy (n=145 total; benralizumab n=73, placebo n=72; median bEOS 450.0 cells/µL, range 300-3860). Within 30 days of an index exacerbation: 14 events (benralizumab) vs 62 events (placebo), RR 0.40 (95% CI: 0.22-0.72; P=0.0022) for recurrent moderate/severe exacerbation. Eosinophil ≥300/µL responder subgroup: recurrent exacerbation RR, 90-day window, benralizumab 100 mg vs placebo (POOLED GALATHEA+TERRANOVA, n=145): Same responder subpopulation (n=145). Within 90 days of an index exacerbation: 48 events (benralizumab) vs 127 events (placebo), RR 0.58 (95% CI: 0.41-0.81; P=0.0017) for recurrent moderate/severe exacerbation. Exacerbation: rate ratio vs placebo, 95% CI: 30 mg vs placebo RR 0.96 (95% CI: 0.80-1.15), P=0.65; 100 mg vs placebo RR 0.83 (0.69-1.00), P=0.0525. Pooled EOS≥300/µL + ≥3 prior-yr exacerbations subgroup (100mg only, GALATHEA+TERRANOVA pooled): recurrent SEVERE exacerbation RR vs placebo, within 30 days: Rate ratio 0.33 (95% CI: 0.08-1.30; P=0.1133), not statistically significant; events benralizumab 3 vs placebo 17 (pooled GALATHEA+TERRANOVA, 100 mg arm only: NOT separable to GALATHEA alone). Pooled EOS≥300/µL + ≥3 prior-yr exacerbations subgroup (100mg only, GALATHEA+TERRANOVA pooled): recurrent SEVERE exacerbation RR vs placebo, within 90 days: Rate ratio 0.48 (95% CI: 0.16-1.43; P=0.1862), not statistically significant; events benralizumab 8 vs placebo 24 (pooled GALATHEA+TERRANOVA, 100 mg arm only: NOT separable to GALATHEA alone). Pooled EOS≥300/µL + ≥3 prior-yr exacerbations subgroup (100mg only, GALATHEA+TERRANOVA pooled): recurrent exacerbation RR vs placebo, within 30 days: Rate ratio 0.40 (95% CI: 0.22-0.72; P=0.0022) for recurrent moderate/severe exacerbation within 30 days of an initial exacerbation; events benralizumab 14 vs placebo 62; subgroup N benralizumab 73, placebo 72 (pooled GALATHEA+TERRANOVA, 100 mg arm only: NOT separable to GALATHEA alone). Subgroup additionally required triple therapy at baseline, baseline blood eosinophils ≥300 cells/µL, ≥3 exacerbations in the previous year, and ≥1 on-study exacerbation. Pooled EOS≥300/µL + ≥3 prior-yr exacerbations subgroup (100mg only, GALATHEA+TERRANOVA pooled): recurrent exacerbation RR vs placebo, within 90 days: Rate ratio: 0.58 (95% CI: 0.41-0.81; P=0.0017) for recurrent moderate/severe exacerbation within 90 days of an initial exacerbation; events benralizumab 48 vs placebo 127 (pooled GALATHEA+TERRANOVA, 100 mg arm only: NOT separable to GALATHEA alone).	PMID 37533773; ClinicalTrials.gov NCT02138916; PMID 31575508; PMC10390712; PMID 31112385	No: transcribed as reported; S1 adds no calculation.	
	Active: STARTED: benralizumab 30 mg=554; benralizumab 100 mg=552 (baseline denom confirms 554 and 552). Placebo: STARTED: placebo=550 (baseline denom confirms Placebo=550)	Pre-bronchodilator FEV1. Outcome-specific timepoint NR; trial follow-up context: Primary endpoint (annualized exacerbation rate) assessed over the 56-week treatment period (trial title also specifies “56 week”). CT.Gov secondary-outcome time frames additionally show 48 weeks (duration of study treatment administration, per protocol) and 60 weeks (immunogenicity follow-up, per protocol): different outcome-specific windows, not contradictions of the core 56-week figure.	FEV1: LS-mean change from baseline, active arm(s): benralizumab 30 mg=0.014 L; benralizumab 100 mg=0.031 LFEV1: LS-mean change from baseline, placebo arm: placebo=0.010 L	FEV1: LS-mean difference vs placebo (mL), SE/95% CI: 30 mg vs placebo: mean difference (final values)=0.007 L, CI: -0.035-0.048, p=0.7550; 100 mg vs placebo: 0.021 L,, CI: -0.021-0.062) p=0.3285Pooled EOS≥220/µL + ≥3 prior-yr exacerbations (± triple therapy, ± FEV1<40%) subgroups: exacerbation RR vs placebo by dose (GALATHEA+TERRANOVA pooled predictive-response analysis, N=2665-3910 depending on subgroup): Pooled GALATHEA+TERRANOVA predictive-response analysis (2665 patients with elevated baseline eosinophils; other subgroups up to N=3910 total analyzed): EOS≥220/µL + ≥3 prior-yr exacerbations: RR 0.69 (95% CI: 0.56-0.83) for 100 mg, 0.86 (0.71-1.04) for 30 mg. Post-BD FEV1<40% predicted: RR 0.76 (0.64-0.91) for 100 mg, 0.90 (0.76-1.06) for 30 mg. Post-BD bronchodilator response ≥15%: RR 0.67 (0.54-0.83) for 100 mg, 0.87 (0.71-1.07) for 30 mg. Combined subgroup (elevated EOS + ≥3 exacerbations/yr + triple therapy): RR 0.70 (0.56-0.88) for 100 mg, 0.99 (0.79-1.23) for 30 mg. CAUTION: all pooled across GALATHEA+TERRANOVA, not separable to GALATHEA alone.	ClinicalTrials.gov NCT02138916; PMID 31575508	No: transcribed as reported; S1 adds no calculation.	
	Active: STARTED: benralizumab 30 mg=554; benralizumab 100 mg=552 (baseline denom confirms 554 and 552). Placebo: STARTED: placebo=550 (baseline denom confirms Placebo=550)	Post-bronchodilator FEV1. Outcome-specific timepoint NR; trial follow-up context: Primary endpoint (annualized exacerbation rate) assessed over the 56-week treatment period (trial title also specifies “56 week”). CT.Gov secondary-outcome time frames additionally show 48 weeks (duration of study treatment administration, per protocol) and 60 weeks (immunogenicity follow-up, per protocol): different outcome-specific windows, not contradictions of the core 56-week figure.	NR/not obtainable: no per-arm summary was present; see comparative/qualitative result.	Eosinophil ≥220/µL subgroup: post-BD FEV1 <40% predicted RR vs placebo (POOLED GALATHEA+TERRANOVA, not GALATHEA-only): CAUTION: pooled, not GALATHEA-specific. EOS≥220/µL with post-BD FEV1 <40% predicted: benralizumab 100 mg RR 0.76 (95% CI: 0.64-0.91); 30 mg RR 0.90 (95% CI: 0.76-1.06) vs placebo.	PMID 31575508	No: transcribed as reported; S1 adds no calculation.	
	Active: STARTED: benralizumab 30 mg=554; benralizumab 100 mg=552 (baseline denom confirms 554 and 552). Placebo: STARTED: placebo=550 (baseline denom confirms Placebo=550)	SGRQ total score Outcome-specific timepoint NR; trial follow-up context: Primary endpoint (annualized exacerbation rate) assessed over the 56-week treatment period (trial title also specifies “56 week”). CT.Gov secondary-outcome time frames additionally show 48 weeks (duration of study treatment administration, per protocol) and 60 weeks (immunogenicity follow-up, per protocol): different outcome-specific windows, not contradictions of the core 56-week figure.	SGRQ: LS-mean change from baseline, active arm(s): benralizumab 30 mg=-5.025; benralizumab 100 mg=-6.723SGRQ: LS-mean change from baseline, placebo arm: placebo=-3.913	SGRQ: LS-mean difference vs placebo, SE/95% CI: 30 mg vs placebo: Mean Difference (Final Values)=-1.011, CI: -2.887-0.865) p=0.2906; 100 mg vs placebo: -2.136, CI: -4.020-0.251) p=0.0264	ClinicalTrials.gov NCT02138916	No: transcribed as reported; S1 adds no calculation.	
	Active: STARTED: benralizumab 30 mg=554; benralizumab 100 mg=552 (baseline denom confirms 554 and 552). Placebo: STARTED: placebo=550 (baseline denom confirms Placebo=550)	E-RS:COPD. Outcome-specific timepoint NR; trial follow-up context: Primary endpoint (annualized exacerbation rate) assessed over the 56-week treatment period (trial title also specifies “56 week”). CT.Gov secondary-outcome time frames additionally show 48 weeks (duration of study treatment administration, per protocol) and 60 weeks (immunogenicity follow-up, per protocol): different outcome-specific windows, not contradictions of the core 56-week figure.	E-RS: LS-mean change from baseline, active arm(s): benralizumab 30 mg=-1.085; benralizumab 100 mg=-1.354E-RS: LS-mean change from baseline, placebo arm: placebo=-0.504	E-RS: LS-mean difference vs placebo, SE/95% CI: 30 mg vs placebo: Mean Difference (Final Values)=-0.585, CI: -1.260-0.089) p=0.0889; 100 mg vs placebo: -0.703, CI: -1.378-0.028) p=0.0413	ClinicalTrials.gov NCT02138916	No: transcribed as reported; S1 adds no calculation.	
	Active: STARTED: benralizumab 30 mg=554; benralizumab 100 mg=552 (baseline denom confirms 554 and 552). Placebo: STARTED: placebo=550 (baseline denom confirms Placebo=550)	Time to first exacerbation. Outcome-specific timepoint NR; trial follow-up context: Primary endpoint (annualized exacerbation rate) assessed over the 56-week treatment period (trial title also specifies “56 week”). CT.Gov secondary-outcome time frames additionally show 48 weeks (duration of study treatment administration, per protocol) and 60 weeks (immunogenicity follow-up, per protocol): different outcome-specific windows, not contradictions of the core 56-week figure.	NR/not obtainable: no trial-specific numeric result for this domain was present in the extraction workbook.	NR/not obtainable: no trial-specific numeric result for this domain was present in the extraction workbook.	Extraction workbook (no matching trial-specific outcome row).	Not applicable: NR/not obtainable.	
	Active: STARTED: benralizumab 30 mg=554; benralizumab 100 mg=552 (baseline denom confirms 554 and 552). Placebo: STARTED: placebo=550 (baseline denom confirms Placebo=550)	Rescue-medication use. Outcome-specific timepoint NR; trial follow-up context: Primary endpoint (annualized exacerbation rate) assessed over the 56-week treatment period (trial title also specifies “56 week”). CT.Gov secondary-outcome time frames additionally show 48 weeks (duration of study treatment administration, per protocol) and 60 weeks (immunogenicity follow-up, per protocol): different outcome-specific windows, not contradictions of the core 56-week figure.	NR/not obtainable: no trial-specific numeric result for this domain was present in the extraction workbook.	NR/not obtainable: no trial-specific numeric result for this domain was present in the extraction workbook.	Extraction workbook (no matching trial-specific outcome row).	Not applicable: NR/not obtainable.	
	Active: STARTED: benralizumab 30 mg=554; benralizumab 100 mg=552 (baseline denom confirms 554 and 552). Placebo: STARTED: placebo=550 (baseline denom confirms Placebo=550)	Any treatment-emergent adverse event. Outcome-specific timepoint NR; trial follow-up context: Primary endpoint (annualized exacerbation rate) assessed over the 56-week treatment period (trial title also specifies “56 week”). CT.Gov secondary-outcome time frames additionally show 48 weeks (duration of study treatment administration, per protocol) and 60 weeks (immunogenicity follow-up, per protocol): different outcome-specific windows, not contradictions of the core 56-week figure.	Safety: TEAE, n/N per arm: Aggregate any-TEAE n/N remains not obtainable. The raw registry contains an adverse-events module with a 3% reporting threshold and posts participants with threshold-reported other/non-serious events: benralizumab 30 mg 293/554, 100 mg 318/552, and placebo 282/550. These threshold-reported counts are not equivalent to aggregate any-TEAE incidence; the publication reports only that adverse-event types and frequencies were similar (not obtainable).	NR/not obtainable: no comparative effect with precision was present; see per-arm summary.	ClinicalTrials.gov NCT02138916	Not applicable: NR/not obtainable.	
	Active: STARTED: benralizumab 30 mg=554; benralizumab 100 mg=552 (baseline denom confirms 554 and 552). Placebo: STARTED: placebo=550 (baseline denom confirms Placebo=550)	Serious adverse events. Outcome-specific timepoint NR; trial follow-up context: Primary endpoint (annualized exacerbation rate) assessed over the 56-week treatment period (trial title also specifies “56 week”). CT.Gov secondary-outcome time frames additionally show 48 weeks (duration of study treatment administration, per protocol) and 60 weeks (immunogenicity follow-up, per protocol): different outcome-specific windows, not contradictions of the core 56-week figure.	Safety: SAE, n/N per arm: benralizumab 30 mg: serious. Affected=151/554; benralizumab 100 mg=177/552; placebo=176/550	NR/not obtainable: no comparative effect with precision was present; see per-arm summary.	ClinicalTrials.gov NCT02138916	No: transcribed as reported; S1 adds no calculation.	
	Active: STARTED: benralizumab 30 mg=554; benralizumab 100 mg=552 (baseline denom confirms 554 and 552). Placebo: STARTED: placebo=550 (baseline denom confirms Placebo=550)	AEs leading to discontinuation. Outcome-specific timepoint NR; trial follow-up context: Primary endpoint (annualized exacerbation rate) assessed over the 56-week treatment period (trial title also specifies “56 week”). CT.Gov secondary-outcome time frames additionally show 48 weeks (duration of study treatment administration, per protocol) and 60 weeks (immunogenicity follow-up, per protocol): different outcome-specific windows, not contradictions of the core 56-week figure.	Safety: discontinuation due to AE, n/N per arm: benralizumab 30 mg 4/554 started; 100 mg 4/552; placebo 3/550 withdrew because of an adverse event.	NR/not obtainable: no comparative effect with precision was present; see per-arm summary.	ClinicalTrials.gov NCT02138916	No: transcribed as reported; S1 adds no calculation.	
	Active: STARTED: benralizumab 30 mg=554; benralizumab 100 mg=552 (baseline denom confirms 554 and 552). Placebo: STARTED: placebo=550 (baseline denom confirms Placebo=550)	All-cause death. Outcome-specific timepoint NR; trial follow-up context: Primary endpoint (annualized exacerbation rate) assessed over the 56-week treatment period (trial title also specifies “56 week”). CT.Gov secondary-outcome time frames additionally show 48 weeks (duration of study treatment administration, per protocol) and 60 weeks (immunogenicity follow-up, per protocol): different outcome-specific windows, not contradictions of the core 56-week figure.	Safety: death, n/N per arm: benralizumab 30 mg: deaths=15/554; benralizumab 100 mg=11/552; placebo=13/550	NR/not obtainable: no comparative effect with precision was present; see per-arm summary.	ClinicalTrials.gov NCT02138916	No: transcribed as reported; S1 adds no calculation.	
	Active: STARTED: benralizumab 30 mg=554; benralizumab 100 mg=552 (baseline denom confirms 554 and 552). Placebo: STARTED: placebo=550 (baseline denom confirms Placebo=550)	AEs of special interest. Outcome-specific timepoint NR; trial follow-up context: Primary endpoint (annualized exacerbation rate) assessed over the 56-week treatment period (trial title also specifies “56 week”). CT.Gov secondary-outcome time frames additionally show 48 weeks (duration of study treatment administration, per protocol) and 60 weeks (immunogenicity follow-up, per protocol): different outcome-specific windows, not contradictions of the core 56-week figure.	NR/not obtainable: no trial-specific numeric result for this domain was present in the extraction workbook.	NR/not obtainable: no trial-specific numeric result for this domain was present in the extraction workbook.	Extraction workbook (no matching trial-specific outcome row).	Not applicable: NR/not obtainable.	
S1.7 TERRANOVA (NCT02155660) [25]							
TERRANOVA	Active: STARTED: benralizumab 10 mg=562; benralizumab 30 mg=562; benralizumab 100 mg=562. Placebo: STARTED=568	Moderate/severe exacerbation rate30 days, 90 days	Eosinophil <220/µL subgroup - annualized exacerbation rate, TERRANOVA (all arms): benralizumab 10 mg=1.23(1.03-1.46); benralizumab 30 mg=1.27(1.05-1.53); benralizumab 100 mg=1.21(1.00-1.45); placebo=1.18(0.98-1.41). Exacerbation - adjusted annualized rate, active arm 100 mg, EOS≥220/µL subgroup: 1.09 per year (95% CI: 0.96-1.23). Exacerbation - adjusted annualized rate, active arms (all 3 doses), EOS≥220/µL subgroup: 10 mg: 0.99/year (95% CI: 0.87-1.13); 30 mg: 1.21/year (95% CI: 1.08-1.37); 100 mg: 1.09/year (95% CI: 0.96-1.23) - EOS≥220 cells/µL primary analysis population. Exacerbation - adjusted annualized rate, placebo arm, EOS≥220/µL subgroup: 1.17 exacerbations/year (95% CI: 1.04-1.32). Exacerbation - adjusted annualized rate, placebo arm, EOS≥220/µL subgroup: 1.17 per year (95% CI: 1.04-1.32).	Eosinophil <150/150-<300/≥300 cells/µL: exacerbation RR vs placebo (95% CI), any outcome: Not obtainable in this pass at the specific 150/150-300/≥300 cells/µL cutpoints requested: TERRANOVA’s own stratification uses a 220/mm3 cutoff, and no data at 150/300 cutpoints were found in CT.Gov or the NEJM abstract. A related but non-equivalent post-hoc analysis exists at an EOS≥300 cutoff combined with a ≥3 exacerbations/year requirement (pooled GALATHEA+TERRANOVA, PMID 37533773): see the four responder-subgroup rows below; it does not answer this field’s 150/300 cutpoint request and is not TERRANOVA-specific (not obtainable).Eosinophil <220/mm3 cohort - exacerbation rate/RR vs placebo: Rates: Benra 10 mg=1.23(1.03-1.46); 30 mg=1.27(1.05-1.53); 100 mg=1.21(1.00-1.45); placebo=1.18(0.98-1.41). RR vs placebo: 1.04, CI: 0.82-1.32); 1.08, CI: 0.84-1.37); 1.02, CI: 0.80-1.30)Eosinophil ≥220/µL + ≥3 prior-yr exacerbations subgroup - exacerbation RR vs placebo (95% CI), benralizumab 30mg/100mg (pooled GALATHEA+TERRANOVA, single-pass, pass2 only): RR 0.69 (95% CI: 0.56-0.83), benralizumab 100 mg vs placebo; RR 0.86 (95% CI: 0.71-1.04), benralizumab 30 mg vs placebo - in patients with baseline blood eosinophils ≥220/µL AND ≥3 exacerbations in the prior year, pooled post hoc analysis of GALATHEA+TERRANOVA combined (n=2665 patients with elevated eosinophil counts). NOT a TERRANOVA-only estimate. Eosinophil ≥300 cells/µL + ≥3 exacerbations/year responder subgroup - recurrent SEVERE-only exacerbation RR vs placebo within 30 days (pooled GALATHEA+TERRANOVA, benralizumab 100 mg only): RR 0.33 (95% CI: 0.08-1.30), P=0.1133 (not statistically significant); benralizumab 3 events vs placebo 17 events, in the pooled 145-patient (73 benralizumab/72 placebo) GALATHEA+TERRANOVA post hoc responder subgroup. Eosinophil ≥300 cells/µL + ≥3 exacerbations/year responder subgroup - recurrent SEVERE-only exacerbation RR vs placebo within 90 days (pooled GALATHEA+TERRANOVA, benralizumab 100 mg only): RR 0.48 (95% CI: 0.16-1.43), P=0.1862 (not statistically significant); benralizumab 8 events vs placebo 24 events, same pooled 145-patient responder subgroup. Eosinophil ≥300 cells/µL + ≥3 exacerbations/year responder subgroup - recurrent moderate/severe exacerbation RR vs placebo within 30 days (pooled GALATHEA+TERRANOVA, benralizumab 100 mg only): RR 0.40 (95% CI: 0.22-0.72), P=0.0022; benralizumab n=73 (14 recurrent events) vs placebo n=72 (62 recurrent events); pooled GALATHEA+TERRANOVA responder subgroup (~6.5% of combined trial population, benralizumab 73/1114, placebo 72/1118).Eosinophil ≥300 cells/µL + ≥3 exacerbations/year responder subgroup - recurrent moderate/severe exacerbation RR vs placebo within 90 days (pooled GALATHEA+TERRANOVA, benralizumab 100 mg only): RR 0.58 (95% CI: 0.41-0.81), P=0.0017, same pooled 145-patient (n=73/72) responder subgroup; benralizumab 48 recurrent events vs placebo 127 recurrent events. Exacerbation - rate ratio vs placebo, 100 mg, EOS≥220/µL subgroup: benralizumab 100 mg vs placebo RR 0.93 (95% CI: 0.78-1.10), P=0.3988. Exacerbation - rate ratio vs placebo, 30 mg, EOS≥220/µL subgroup: benralizumab 30 mg vs placebo RR 1.04 (95% CI: 0.88-1.23), P=0.6575. Exacerbation - rate ratio vs placebo, all 3 doses, EOS≥220/µL subgroup: benralizumab vs placebo: 10 mg RR 0.85 (95% CI: 0.71-1.01), P=0.0638; 30 mg RR 1.04 (0.88-1.23), P=0.6575; 100 mg RR 0.93 (0.78-1.10), P=0.3988.	CT.Gov + NEJM abstract reviewClinicalTrials.gov NCT02155660; PMID 31575508; DOI 10.1016/S2213-2600(19; PMC10390712; PMID 37533773; DOI 10.2147/COPD.S418944; PMID 31112385	No: transcribed as reported; S1 adds no calculation.	
	Active: STARTED: benralizumab 10 mg=562; benralizumab 30 mg=562; benralizumab 100 mg=562. Placebo: STARTED=568	Pre-bronchodilator FEV1. Outcome-specific timepoint NR; trial follow-up context: 56-week double-blind treatment period with primary/secondary efficacy endpoints at week 56; immunogenicity followed to week 60 per protocol; a secondary CT.Gov outcome (’Duration of Study Treatment Administration’) is defined against a 48-week per-protocol treatment duration.	FEV1: LS-mean change from baseline, active arm(s): benralizumab 10 mg=0.021; benralizumab 30 mg=0.011; benralizumab 100 mg=0.033 (L)FEV1: LS-mean change from baseline, placebo arm: placebo=0.016 (L)	FEV1: LS-mean difference vs placebo (mL), SE/95% CI: Benra10 vs Placebo: 0.015, CI: -0.029,0.059) p=0.5043; Benra30: -0.007, CI: -0.051,0.037) p=0.7691; Benra100: 0.02, CI: -0.024,0.064) p=0.3767 (liters)	ClinicalTrials.gov NCT02155660	No: transcribed as reported; S1 adds no calculation.	
	Active: STARTED: benralizumab 10 mg=562; benralizumab 30 mg=562; benralizumab 100 mg=562. Placebo: STARTED=568	Post-bronchodilator FEV1. Outcome-specific timepoint NR; trial follow-up context: 56-week double-blind treatment period with primary/secondary efficacy endpoints at week 56; immunogenicity followed to week 60 per protocol; a secondary CT.Gov outcome (’Duration of Study Treatment Administration’) is defined against a 48-week per-protocol treatment duration.	NR/not obtainable: no trial-specific numeric result for this domain was present in the extraction workbook.	NR/not obtainable: no trial-specific numeric result for this domain was present in the extraction workbook.	Extraction workbook (no matching trial-specific outcome row).	Not applicable: NR/not obtainable.	
	Active: STARTED: benralizumab 10 mg=562; benralizumab 30 mg=562; benralizumab 100 mg=562. Placebo: STARTED=568	SGRQ total score Outcome-specific timepoint NR; trial follow-up context: 56-week double-blind treatment period with primary/secondary efficacy endpoints at week 56; immunogenicity followed to week 60 per protocol; a secondary CT.Gov outcome (’Duration of Study Treatment Administration’) is defined against a 48-week per-protocol treatment duration.	SGRQ: LS-mean change from baseline, active arm(s): benralizumab 10 mg=-7.733; benralizumab 30 mg=-8.674; benralizumab 100 mg=-7.257SGRQ: LS-mean change from baseline, placebo arm: placebo=-6.863	SGRQ: LS-mean difference vs placebo, SE/95% CI: Benra10 vs Placebo: -1.011, CI: -3.192,1.171) p=0.3636; Benra30: -1.388, CI: -3.562,0.786) p=0.2106; Benra100: -0.602, CI: -2.763,1.560) p=0.5851	ClinicalTrials.gov NCT02155660	No: transcribed as reported; S1 adds no calculation.	
	Active: STARTED: benralizumab 10 mg=562; benralizumab 30 mg=562; benralizumab 100 mg=562. Placebo: STARTED=568	E-RS:COPD. Outcome-specific timepoint NR; trial follow-up context: 56-week double-blind treatment period with primary/secondary efficacy endpoints at week 56; immunogenicity followed to week 60 per protocol; a secondary CT.Gov outcome (’Duration of Study Treatment Administration’) is defined against a 48-week per-protocol treatment duration.	E-RS: LS-mean change from baseline, active arm(s): benralizumab 10 mg=-1.657; benralizumab 30 mg=-2.219; benralizumab 100 mg=-1.593E-RS: LS-mean change from baseline, placebo arm: placebo=-1.137	E-RS: LS-mean difference vs placebo, SE/95% CI: Benra10 vs Placebo: Mean Difference=-0.4, CI: -1.102,0.301) p=0.2636; Benra30: -0.296, CI: -0.998,0.406) p=0.4087; Benra100: -0.425, CI: -1.125,0.275) p=0.2336	ClinicalTrials.gov NCT02155660	No: transcribed as reported; S1 adds no calculation.	
	Active: STARTED: benralizumab 10 mg=562; benralizumab 30 mg=562; benralizumab 100 mg=562. Placebo: STARTED=568	Time to first exacerbation. Outcome-specific timepoint NR; trial follow-up context: 56-week double-blind treatment period with primary/secondary efficacy endpoints at week 56; immunogenicity followed to week 60 per protocol; a secondary CT.Gov outcome (’Duration of Study Treatment Administration’) is defined against a 48-week per-protocol treatment duration.	NR/not obtainable: no trial-specific numeric result for this domain was present in the extraction workbook.	NR/not obtainable: no trial-specific numeric result for this domain was present in the extraction workbook.	Extraction workbook (no matching trial-specific outcome row).	Not applicable: NR/not obtainable.	
	Active: STARTED: benralizumab 10 mg=562; benralizumab 30 mg=562; benralizumab 100 mg=562. Placebo: STARTED=568	Rescue-medication use. Outcome-specific timepoint NR; trial follow-up context: 56-week double-blind treatment period with primary/secondary efficacy endpoints at week 56; immunogenicity followed to week 60 per protocol; a secondary CT.Gov outcome (’Duration of Study Treatment Administration’) is defined against a 48-week per-protocol treatment duration.	NR/not obtainable: no trial-specific numeric result for this domain was present in the extraction workbook.	NR/not obtainable: no trial-specific numeric result for this domain was present in the extraction workbook.	Extraction workbook (no matching trial-specific outcome row).	Not applicable: NR/not obtainable.	
	Active: STARTED: benralizumab 10 mg=562; benralizumab 30 mg=562; benralizumab 100 mg=562. Placebo: STARTED=568	Any treatment-emergent adverse event. Outcome-specific timepoint NR; trial follow-up context: 56-week double-blind treatment period with primary/secondary efficacy endpoints at week 56; immunogenicity followed to week 60 per protocol; a secondary CT.Gov outcome (’Duration of Study Treatment Administration’) is defined against a 48-week per-protocol treatment duration.	Safety: TEAE, n/N per arm: Aggregate any-TEAE n/N remains not obtainable. The registry does contain an adverse-events module with a 3% reporting threshold and reports participants with threshold-reported other/non-serious events: benralizumab 10 mg 251/561, 30 mg 233/563, 100 mg 238/562, placebo 260/568. Those threshold-reported counts are not equivalent to any TEAE incidence. The NEJM abstract states qualitatively that adverse-event types and frequencies were similar (not obtainable).	NR/not obtainable: no comparative effect with precision was present; see per-arm summary.	ClinicalTrials.gov NCT02155660; PMID 31112385	Not applicable: NR/not obtainable.	
	Active: STARTED: benralizumab 10 mg=562; benralizumab 30 mg=562; benralizumab 100 mg=562. Placebo: STARTED=568	Serious adverse events. Outcome-specific timepoint NR; trial follow-up context: 56-week double-blind treatment period with primary/secondary efficacy endpoints at week 56; immunogenicity followed to week 60 per protocol; a secondary CT.Gov outcome (’Duration of Study Treatment Administration’) is defined against a 48-week per-protocol treatment duration.	Safety: SAE, n/N per arm: Benra 10 mg=144/561; Benra 30 mg=177/563; Benra 100 mg=127/562; placebo=158/568	NR/not obtainable: no comparative effect with precision was present; see per-arm summary.	ClinicalTrials.gov NCT02155660	No: transcribed as reported; S1 adds no calculation.	
	Active: STARTED: benralizumab 10 mg=562; benralizumab 30 mg=562; benralizumab 100 mg=562. Placebo: STARTED=568	AEs leading to discontinuation. Outcome-specific timepoint NR; trial follow-up context: 56-week double-blind treatment period with primary/secondary efficacy endpoints at week 56; immunogenicity followed to week 60 per protocol; a secondary CT.Gov outcome (’Duration of Study Treatment Administration’) is defined against a 48-week per-protocol treatment duration.	Safety: discontinuation due to AE, n/N per arm: benralizumab 10 mg 2/562 started; 30 mg 6/562; 100 mg 3/562; placebo 1/568 withdrew because of an adverse event.	NR/not obtainable: no comparative effect with precision was present; see per-arm summary.	ClinicalTrials.gov NCT02155660	No: transcribed as reported; S1 adds no calculation.	
	Active: STARTED: benralizumab 10 mg=562; benralizumab 30 mg=562; benralizumab 100 mg=562. Placebo: STARTED=568	All-cause death. Outcome-specific timepoint NR; trial follow-up context: 56-week double-blind treatment period with primary/secondary efficacy endpoints at week 56; immunogenicity followed to week 60 per protocol; a secondary CT.Gov outcome (’Duration of Study Treatment Administration’) is defined against a 48-week per-protocol treatment duration.	Safety: death, n/N per arm: Benra 10 mg=17/561; Benra 30 mg=21/563; Benra 100 mg=17/562; placebo=19/568	NR/not obtainable: no comparative effect with precision was present; see per-arm summary.	ClinicalTrials.gov NCT02155660	No: transcribed as reported; S1 adds no calculation.	
	Active: STARTED: benralizumab 10 mg=562; benralizumab 30 mg=562; benralizumab 100 mg=562. Placebo: STARTED=568	AEs of special interest. Outcome-specific timepoint NR; trial follow-up context: 56-week double-blind treatment period with primary/secondary efficacy endpoints at week 56; immunogenicity followed to week 60 per protocol; a secondary CT.Gov outcome (’Duration of Study Treatment Administration’) is defined against a 48-week per-protocol treatment duration.	NR/not obtainable: no trial-specific numeric result for this domain was present in the extraction workbook.	NR/not obtainable: no trial-specific numeric result for this domain was present in the extraction workbook.	Extraction workbook (no matching trial-specific outcome row).	Not applicable: NR/not obtainable.	
S1.8 ALIENTO (NCT05037929) [18]							
ALIENTO	Active: Actual randomized enrollment in the registry: 1334. Primary report initiated-treatment/as-treated counts: astegolimab Q2W n=433 and Q4W n=437 (total initiated treatment across all arms 1301). These are distinct denominator conventions. Placebo: Actual randomized enrollment in the registry: 1334. Primary report placebo initiated-treatment/as-treated n=431; initiated-treatment total across all arms 1301. These are distinct denominator conventions.	Moderate/severe exacerbation rate. Outcome-specific timepoint NR; trial follow-up context: 52-week treatment period (primary endpoint = annualized exacerbation rate over 52 weeks); primary completion 2025-02-24; study completion 2025-07-07 per CT.Gov	Exacerbation: adjusted annualized rate, active arm(s): NR/not obtainable: workbook value cell is empty (not obtainable). Exacerbation: adjusted annualized rate, placebo arm: NR/not obtainable: workbook value cell is empty (not obtainable).	Exacerbation: rate ratio vs placebo, 95% CI (ALIENTO primary endpoint): ALIENTO-specific primary endpoint (annualized rate of moderate/severe exacerbations, as-treated population): astegolimab Q2W vs placebo adjusted rate ratio 0.85 (95% CI: 0.72-1.00; p=0.049); astegolimab Q4W vs placebo adjusted rate ratio 0.93 (95% CI: 0.79-1.10; p=0.38). Distinct from ARNASA’s own RRs (0.85 (0.72-1.01; p=0.068) Q2W; 0.82 (0.70-0.98; p=0.024) Q4W) reported in the same paper, and distinct from the pooled ALIENTO+ARNASA figures in PMID 42148875: none of these should be misattributed to ALIENTO’s primary endpoint (EXTRA: pooled, NOT ALIENTO-alone). Exacerbation rate ratio vs placebo, ALIENTO+ARNASA pooled ITT (N=2682): Pooled ITT (N=2682): Q2W adjusted rate ratio 0.85 (95% CI: 0.76-0.96; p=0.0077), a 15% reduction; Q4W adjusted rate ratio 0.88 (95% CI: 0.78-0.99; p=0.0265), a 12% reduction. Pooled severe-exacerbation-only rate ratio for Q2W: 0.68 (95% CI: 0.52-0.87; p=0.0028). These are pooled ALIENTO+ARNASA figures from PMID 42148875 and must NOT be attributed to ALIENTO alone.	ClinicalTrials.gov NCT05037929; PMID 42150581; PMID 42148875	No: transcribed as reported; S1 adds no calculation.	
	Active: Actual randomized enrollment in the registry: 1334. Primary report initiated-treatment/as-treated counts: astegolimab Q2W n=433 and Q4W n=437 (total initiated treatment across all arms 1301). These are distinct denominator conventions. Placebo: Actual randomized enrollment in the registry: 1334. Primary report placebo initiated-treatment/as-treated n=431; initiated-treatment total across all arms 1301. These are distinct denominator conventions.	Pre-bronchodilator FEV1. Outcome-specific timepoint NR; trial follow-up context: 52-week treatment period (primary endpoint = annualized exacerbation rate over 52 weeks); primary completion 2025-02-24; study completion 2025-07-07 per CT.Gov	FEV1: LS-mean change from baseline, active arm(s): NR/not obtainable: workbook value cell is empty (not obtainable). FEV1: LS-mean change from baseline, placebo arm: NR/not obtainable: workbook value cell is empty (not obtainable).	Eosinophil subgroup: any other outcome (FEV1/SGRQ/E-RS/safety): Not obtainable: no eosinophil-stratified FEV1, SGRQ, E-RS, or safety subgroup data for ALIENTO found in either queried abstract (not obtainable). FEV1: LS-mean difference vs placebo (mL), SE/95% CI: NR/not obtainable: workbook value cell is empty (not obtainable).	Lancet + AJRCCM abstracts ClinicalTrials.gov NCT05037929	Not applicable: NR/not obtainable.	
	Active: Actual randomized enrollment in the registry: 1334. Primary report initiated-treatment/as-treated counts: astegolimab Q2W n=433 and Q4W n=437 (total initiated treatment across all arms 1301). These are distinct denominator conventions. Placebo: Actual randomized enrollment in the registry: 1334. Primary report placebo initiated-treatment/as-treated n=431; initiated-treatment total across all arms 1301. These are distinct denominator conventions.	Post-bronchodilator FEV1. Outcome-specific timepoint NR; trial follow-up context: 52-week treatment period (primary endpoint = annualized exacerbation rate over 52 weeks); primary completion 2025-02-24; study completion 2025-07-07 per CT.Gov	NR/not obtainable: no trial-specific numeric result for this domain was present in the extraction workbook.	NR/not obtainable: no trial-specific numeric result for this domain was present in the extraction workbook.	Extraction workbook (no matching trial-specific outcome row).	Not applicable: NR/not obtainable.	
	Active: Actual randomized enrollment in the registry: 1334. Primary report initiated-treatment/as-treated counts: astegolimab Q2W n=433 and Q4W n=437 (total initiated treatment across all arms 1301). These are distinct denominator conventions. Placebo: Actual randomized enrollment in the registry: 1334. Primary report placebo initiated-treatment/as-treated n=431; initiated-treatment total across all arms 1301. These are distinct denominator conventions.	SGRQ total score Outcome-specific timepoint NR; trial follow-up context: 52-week treatment period (primary endpoint = annualized exacerbation rate over 52 weeks); primary completion 2025-02-24; study completion 2025-07-07 per CT.Gov	SGRQ: LS-mean change from baseline, active arm(s): NR/not obtainable: workbook value cell is empty (not obtainable).SGRQ: LS-mean change from baseline, placebo arm: NR/not obtainable: workbook value cell is empty (not obtainable).	SGRQ: LS-mean difference vs placebo, SE/95% CI: NR/not obtainable: workbook value cell is empty (not obtainable).	ClinicalTrials.gov NCT05037929	Not applicable: NR/not obtainable.	
	Active: Actual randomized enrollment in the registry: 1334. Primary report initiated-treatment/as-treated counts: astegolimab Q2W n=433 and Q4W n=437 (total initiated treatment across all arms 1301). These are distinct denominator conventions. Placebo: Actual randomized enrollment in the registry: 1334. Primary report placebo initiated-treatment/as-treated n=431; initiated-treatment total across all arms 1301. These are distinct denominator conventions.	E-RS:COPD. Outcome-specific timepoint NR; trial follow-up context: 52-week treatment period (primary endpoint = annualized exacerbation rate over 52 weeks); primary completion 2025-02-24; study completion 2025-07-07 per CT.Gov	E-RS: LS-mean change from baseline, active arm(s): NR/not obtainable: workbook value cell is empty (not obtainable). E-RS: LS-mean change from baseline, placebo arm: NR/not obtainable: workbook value cell is empty (not obtainable).	E-RS: LS-mean difference vs placebo, SE/95% CI: NR/not obtainable: workbook value cell is empty (not obtainable).	ClinicalTrials.gov NCT05037929	Not applicable: NR/not obtainable.	
	Active: Actual randomized enrollment in the registry: 1334. Primary report initiated-treatment/as-treated counts: astegolimab Q2W n=433 and Q4W n=437 (total initiated treatment across all arms 1301). These are distinct denominator conventions. Placebo: Actual randomized enrollment in the registry: 1334. Primary report placebo initiated-treatment/as-treated n=431; initiated-treatment total across all arms 1301. These are distinct denominator conventions.	Time to first exacerbation. Outcome-specific timepoint NR; trial follow-up context: 52-week treatment period (primary endpoint = annualized exacerbation rate over 52 weeks); primary completion 2025-02-24; study completion 2025-07-07 per CT.Gov	NR/not obtainable: no trial-specific numeric result for this domain was present in the extraction workbook.	NR/not obtainable: no trial-specific numeric result for this domain was present in the extraction workbook.	Extraction workbook (no matching trial-specific outcome row).	Not applicable: NR/not obtainable.	
	Active: Actual randomized enrollment in the registry: 1334. Primary report initiated-treatment/as-treated counts: astegolimab Q2W n=433 and Q4W n=437 (total initiated treatment across all arms 1301). These are distinct denominator conventions. Placebo: Actual randomized enrollment in the registry: 1334. Primary report placebo initiated-treatment/as-treated n=431; initiated-treatment total across all arms 1301. These are distinct denominator conventions.	Rescue-medication use. Outcome-specific timepoint NR; trial follow-up context: 52-week treatment period (primary endpoint = annualized exacerbation rate over 52 weeks); primary completion 2025-02-24; study completion 2025-07-07 per CT.Gov	NR/not obtainable: no trial-specific numeric result for this domain was present in the extraction workbook.	NR/not obtainable: no trial-specific numeric result for this domain was present in the extraction workbook.	Extraction workbook (no matching trial-specific outcome row).	Not applicable: NR/not obtainable.	
	Active: Actual randomized enrollment in the registry: 1334. Primary report initiated-treatment/as-treated counts: astegolimab Q2W n=433 and Q4W n=437 (total initiated treatment across all arms 1301). These are distinct denominator conventions. Placebo: Actual randomized enrollment in the registry: 1334. Primary report placebo initiated-treatment/as-treated n=431; initiated-treatment total across all arms 1301. These are distinct denominator conventions.	Any treatment-emergent adverse event. Outcome-specific timepoint NR; trial follow-up context: 52-week treatment period (primary endpoint = annualized exacerbation rate over 52 weeks); primary completion 2025-02-24; study completion 2025-07-07 per CT.Gov	Safety: TEAE (any adverse event), n/N per arm: Only a whole-trial (not per-arm) figure is available: 1093/1301 (84.0%) of ALIENTO participants (all arms combined) had ≥1 adverse event; most common non-COPD AE was nasopharyngitis; AEs described in the abstract as “balanced between treatments” but no per-arm n/N was reported. CT.Gov posts no numeric results (has_results=false).	(EXTRA) Safety: any TEAE, ALIENTO overall: 1093/1301 (84.0%) of ALIENTO participants had ≥1 adverse event; most common non-COPD AE was nasopharyngitis.	PMID 42150581	No: transcribed as reported; S1 adds no calculation.	
	Active: Actual randomized enrollment in the registry: 1334. Primary report initiated-treatment/as-treated counts: astegolimab Q2W n=433 and Q4W n=437 (total initiated treatment across all arms 1301). These are distinct denominator conventions. Placebo: Actual randomized enrollment in the registry: 1334. Primary report placebo initiated-treatment/as-treated n=431; initiated-treatment total across all arms 1301. These are distinct denominator conventions.	Serious adverse events. Outcome-specific timepoint NR; trial follow-up context: 52-week treatment period (primary endpoint = annualized exacerbation rate over 52 weeks); primary completion 2025-02-24; study completion 2025-07-07 per CT.Gov	Safety: SAE, n/N per arm: NR/not obtainable: workbook value cell is empty (not obtainable).	NR/not obtainable: no comparative effect with precision was present; see per-arm summary.	ClinicalTrials.gov NCT05037929	Not applicable: NR/not obtainable.	
	Active: Actual randomized enrollment in the registry: 1334. Primary report initiated-treatment/as-treated counts: astegolimab Q2W n=433 and Q4W n=437 (total initiated treatment across all arms 1301). These are distinct denominator conventions. Placebo: Actual randomized enrollment in the registry: 1334. Primary report placebo initiated-treatment/as-treated n=431; initiated-treatment total across all arms 1301. These are distinct denominator conventions.	AEs leading to discontinuation. Outcome-specific timepoint NR; trial follow-up context: 52-week treatment period (primary endpoint = annualized exacerbation rate over 52 weeks); primary completion 2025-02-24; study completion 2025-07-07 per CT.Gov	Safety: discontinuation due to AE, n/N per arm: NR/not obtainable: workbook value cell is empty (not obtainable).	NR/not obtainable: no comparative effect with precision was present; see per-arm summary.	ClinicalTrials.gov NCT05037929	Not applicable: NR/not obtainable.	
	Active: Actual randomized enrollment in the registry: 1334. Primary report initiated-treatment/as-treated counts: astegolimab Q2W n=433 and Q4W n=437 (total initiated treatment across all arms 1301). These are distinct denominator conventions. Placebo: Actual randomized enrollment in the registry: 1334. Primary report placebo initiated-treatment/as-treated n=431; initiated-treatment total across all arms 1301. These are distinct denominator conventions.	All-cause death. Outcome-specific timepoint NR; trial follow-up context: 52-week treatment period (primary endpoint = annualized exacerbation rate over 52 weeks); primary completion 2025-02-24; study completion 2025-07-07 per CT.Gov	Safety: death, n/N per arm: Per-arm breakdown not reported in any source queried. ALIENTO-wide: 40/1301 (3.1%) deaths, described as “balanced across treatment groups.” Across ALIENTO+ARNASA combined: 3/2676 (0.1%) deaths investigator-assessed as treatment-related (2676 = ALIENTO 1301 + ARNASA 1375, both stated explicitly in the same abstract): this pooled figure must not be attributed to ALIENTO alone.	(EXTRA) Safety: death, ALIENTO overall: 40/1301 (3.1%) deaths in ALIENTO, “balanced across treatment groups” (no per-arm breakdown given) (EXTRA). Safety: investigator-assessed treatment-related deaths, ALIENTO+ARNASA combined: 3/2676 (0.1%) deaths across both trials combined were investigator-assessed as treatment-related; not broken out by trial or arm. (2676 = ALIENTO 1301 + ARNASA 1375, both stated explicitly in the same abstract.)	PMID 42150581	No: transcribed as reported; S1 adds no calculation.	
	Active: Actual randomized enrollment in the registry: 1334. Primary report initiated-treatment/as-treated counts: astegolimab Q2W n=433 and Q4W n=437 (total initiated treatment across all arms 1301). These are distinct denominator conventions. Placebo: Actual randomized enrollment in the registry: 1334. Primary report placebo initiated-treatment/as-treated n=431; initiated-treatment total across all arms 1301. These are distinct denominator conventions.	AEs of special interest. Outcome-specific timepoint NR; trial follow-up context: 52-week treatment period (primary endpoint = annualized exacerbation rate over 52 weeks); primary completion 2025-02-24; study completion 2025-07-07 per CT.Gov	NR/not obtainable: no trial-specific numeric result for this domain was present in the extraction workbook.	NR/not obtainable: no trial-specific numeric result for this domain was present in the extraction workbook.	Extraction workbook (no matching trial-specific outcome row).	Not applicable: NR/not obtainable.	
S1.9 ARNASA (NCT05595642) [18]							
ARNASA	Active: astegolimab every 2 weeks: n=459. Astegolimab every 4 weeks: n=459 (treatment initiators; Total ARNASA N=1375, initiated treatment: Jan 9, 2023-June 25, 2024; CT.Gov has no per-arm figure). Placebo: 457 (treatment initiators; the abstract does not use the word “randomized,” only “initiated treatment”; CT.Gov gives no per-arm breakdown, only an aggregate enrollment target of 1290, which is lower than and does not reconcile with the abstract's total of 1375 treatment initiators)	Moderate/severe exacerbation rate 52 weeks, 2 weeks, 4 weeks	Exacerbation: adjusted annualized rate, active arm(s): Not obtainable: same reason as placebo-arm row; abstract gives rate ratios only, not absolute per-arm annualized rates (not obtainable). Exacerbation: adjusted annualized rate, placebo arm: Not obtainable: ClinicalTrials.gov results module not posted (has_results=false, confirmed); Lancet abstract reports only the adjusted rate ratio, not the absolute annualized exacerbation rate per arm (not obtainable)	Eosinophil 150-<300 cells/µL: exacerbation RR vs placebo (95% CI), ARNASA: Not obtainable for ARNASA specifically: same reasoning and sources as the <150 cells/µL row: no ARNASA eosinophil-stratified subgroup data in CT.Gov, the index abstract, or the related pooled AJRCCM analysis (not obtainable).Eosinophil <150 cells/µL: exacerbation RR vs placebo (95% CI), ARNASA: Not obtainable for ARNASA specifically. No eosinophil-stratified subgroup exacerbation-rate-ratio data for ARNASA exists in CT.Gov (no results posted), the index Lancet abstract (states the eosinophil-agnostic enrollment premise but gives no subgroup numbers), or the related pooled ALIENTO+ARNASA analysis (Wedzicha et al., AJRCCM 2026, PMID 42148875), which reports only pooled overall ITT rate ratios (N=2682; q2w RR 0.85 (0.76-0.96) p=.0077; q4w RR 0.88 (0.78-0.99) p=.0265; severe-exacerbation q2w RR 0.68 (0.52-0.87) p=.0028): not eosinophil-stratified and not ARNASA-arm-specific (not obtainable).Eosinophil ≥300 cells/µL: exacerbation RR vs placebo (95% CI), ARNASA: Not obtainable for ARNASA specifically: same reasoning and sources as the <150 cells/µL row: no ARNASA eosinophil-stratified subgroup data in CT.Gov, the index abstract, or the related pooled AJRCCM analysis (not obtainable). Exacerbation: rate ratio vs placebo, 95% CI (primary endpoint: annualized rate of moderate/severe exacerbations, 52 weeks): ARNASA astegolimab every 2 weeks vs placebo: adjusted rate ratio 0.85 (95% CI: 0.72-1.01; p=0.068, not statistically significant). ARNASA astegolimab every 4 weeks vs placebo: adjusted rate ratio 0.82 (95% CI: 0.70-0.98; p=0.024, statistically significant).	PubMed PMIDs 42150581, 42148875 PubMed PMIDs 42150581, 42148875; no ARNASA-specific eosinophil-stratified data located PMID 42150581	No: transcribed as reported; S1 adds no calculation.	
	Active: astegolimab every 2 weeks: n=459. Astegolimab every 4 weeks: n=459 (treatment initiators; Total ARNASA N=1375, initiated treatment: Jan 9, 2023-June 25, 2024; CT.Gov has no per-arm figure). Placebo: 457 (treatment initiators; the abstract does not use the word “randomized,” only “initiated treatment”; CT.Gov gives no per-arm breakdown, only an aggregate enrollment target of 1290, which is lower than and does not reconcile with the abstract's total of 1375 treatment initiators)	Pre-bronchodilator FEV1week 52, 44 weeks, 48-week	FEV1: LS-mean change from baseline, active arm(s): Not obtainable: same reason as placebo-arm FEV1 row (not obtainable)FEV1: LS-mean change from baseline, placebo arm: Not obtainable: secondary outcome registered on CT.Gov (confirmed: “Absolute change from baseline in post-bronchodilator FEV1 at week 52”) but unresulted (has_results=false); no numeric value in the Lancet abstract; no PMCID/OA full text (confirmed) (not obtainable)	FEV1: LS-mean difference vs placebo (mL), SE/95% CI: Not obtainable for ARNASA: no results module on CT.Gov, no numeric value in the Lancet abstract, no accessible full text/supplement. Caution (independently confirmed via PMID 35339234): a distinct, non-ARNASA 40 mL FEV1 difference (95% CI: -10 to 90; p=0.094) belongs to the separate, single-center, earlier phase 2a COPD-ST2OP trial (NCT03615040, PMID 35339234; 490 mg astegolimab Q4W for 44 weeks, 48-week primary endpoint) and must not be substituted as an ARNASA estimate (not obtainable).	PubMed PMID 35339234	Not applicable: NR/not obtainable.	
		Post-bronchodilator FEV1. Outcome-specific timepoint NR; trial follow-up context: 52 weeks treatment period; primary endpoint (annualized rate of moderate/severe exacerbations) time_frame = “52 weeks”; all listed secondary endpoints (time to first exacerbation, SGRQ-C, FEV1, E-RS:COPD, annualized severe-exacerbation rate) time_frame = “52 weeks” or “week 52.” Abstract corroborates a 52-week treatment period.	NR/not obtainable: no trial-specific numeric result for this domain was present in the extraction workbook.	NR/not obtainable: no trial-specific numeric result for this domain was present in the extraction workbook.	Extraction workbook (no matching trial-specific outcome row).	Not applicable: NR/not obtainable.	
	Active: astegolimab every 2 weeks: n=459. Astegolimab every 4 weeks: n=459 (treatment initiators; Total ARNASA N=1375, initiated treatment: Jan 9, 2023-June 25, 2024; CT.Gov has no per-arm figure). Placebo: 457 (treatment initiators; the abstract does not use the word “randomized,” only “initiated treatment”; CT.Gov gives no per-arm breakdown, only an aggregate enrollment target of 1290, which is lower than and does not reconcile with the abstract's total of 1375 treatment initiators)	SGRQ total score Outcome-specific timepoint NR; trial follow-up context: 52 weeks treatment period; primary endpoint (annualized rate of moderate/severe exacerbations) time_frame = “52 weeks”; all listed secondary endpoints (time to first exacerbation, SGRQ-C, FEV1, E-RS:COPD, annualized severe-exacerbation rate) time_frame = “52 weeks” or “week 52.” Abstract corroborates a 52-week treatment period.	SGRQ: LS-mean change from baseline, active arm(s): Not obtainable: same reason as placebo-arm SGRQ row (not obtainable)SGRQ: LS-mean change from baseline, placebo arm: Not obtainable: SGRQ-C secondary outcome registered on CT.Gov but unresulted; no numeric value in the Lancet abstract (not obtainable)	SGRQ: LS-mean difference vs placebo, SE/95% CI: Not obtainable: same reason; full text/supplement inaccessible (no PMCID, no OA PDF, confirmed). Note: the distinct COPD-ST2OP trial (PMID 35339234) does report an SGRQ-C mean difference of -3.3 (95% CI: -6.4 to -0.2; p=0.039), but that is NOT ARNASA and must not be substituted (not obtainable).	NR/not obtainable: the source field is empty in the workbook.	Not applicable: NR/not obtainable.	
	Active: astegolimab every 2 weeks: n=459. Astegolimab every 4 weeks: n=459 (treatment initiators; Total ARNASA N=1375, initiated treatment: Jan 9, 2023-June 25, 2024; CT.Gov has no per-arm figure). Placebo: 457 (treatment initiators; the abstract does not use the word “randomized,” only “initiated treatment”; CT.Gov gives no per-arm breakdown, only an aggregate enrollment target of 1290, which is lower than and does not reconcile with the abstract's total of 1375 treatment initiators)	E-RS:COPD. Outcome-specific timepoint NR; trial follow-up context: 52 weeks treatment period; primary endpoint (annualized rate of moderate/severe exacerbations) time_frame = “52 weeks”; all listed secondary endpoints (time to first exacerbation, SGRQ-C, FEV1, E-RS:COPD, annualized severe-exacerbation rate) time_frame = “52 weeks” or “week 52.” Abstract corroborates a 52-week treatment period.	E-RS: LS-mean change from baseline, active arm(s): Not obtainable: same reason as placebo-arm E-RS row (not obtainable)E-RS: LS-mean change from baseline, placebo arm: Not obtainable: E-RS:COPD secondary outcome registered on CT.Gov but unresulted; no numeric value in the Lancet abstract (not obtainable)	E-RS: LS-mean difference vs placebo, SE/95% CI: Not obtainable: same reason; full text/supplement inaccessible (no PMCID, no OA PDF, confirmed) (not obtainable)	NR/not obtainable: the source field is empty in the workbook.	Not applicable: NR/not obtainable.	
	Active: astegolimab every 2 weeks: n=459. Astegolimab every 4 weeks: n=459 (treatment initiators; Total ARNASA N=1375, initiated treatment: Jan 9, 2023-June 25, 2024; CT.Gov has no per-arm figure). Placebo: 457 (treatment initiators; the abstract does not use the word “randomized,” only “initiated treatment”; CT.Gov gives no per-arm breakdown, only an aggregate enrollment target of 1290, which is lower than and does not reconcile with the abstract's total of 1375 treatment initiators)	Time to first exacerbation. Outcome-specific timepoint NR; trial follow-up context: 52 weeks treatment period; primary endpoint (annualized rate of moderate/severe exacerbations) time_frame = “52 weeks”; all listed secondary endpoints (time to first exacerbation, SGRQ-C, FEV1, E-RS:COPD, annualized severe-exacerbation rate) time_frame = “52 weeks” or “week 52.” Abstract corroborates a 52-week treatment period.	NR/not obtainable: no trial-specific numeric result for this domain was present in the extraction workbook.	NR/not obtainable: no trial-specific numeric result for this domain was present in the extraction workbook.	Extraction workbook (no matching trial-specific outcome row).	Not applicable: NR/not obtainable.	
	Active: astegolimab every 2 weeks: n=459. Astegolimab every 4 weeks: n=459 (treatment initiators; Total ARNASA N=1375, initiated treatment: Jan 9, 2023-June 25, 2024; CT.Gov has no per-arm figure). Placebo: 457 (treatment initiators; the abstract does not use the word “randomized,” only “initiated treatment”; CT.Gov gives no per-arm breakdown, only an aggregate enrollment target of 1290, which is lower than and does not reconcile with the abstract's total of 1375 treatment initiators)	Rescue-medication use. Outcome-specific timepoint NR; trial follow-up context: 52 weeks treatment period; primary endpoint (annualized rate of moderate/severe exacerbations) time_frame = “52 weeks”; all listed secondary endpoints (time to first exacerbation, SGRQ-C, FEV1, E-RS:COPD, annualized severe-exacerbation rate) time_frame = “52 weeks” or “week 52.” Abstract corroborates a 52-week treatment period.	NR/not obtainable: no trial-specific numeric result for this domain was present in the extraction workbook.	NR/not obtainable: no trial-specific numeric result for this domain was present in the extraction workbook.	Extraction workbook (no matching trial-specific outcome row).	Not applicable: NR/not obtainable.	
	Active: astegolimab every 2 weeks: n=459. Astegolimab every 4 weeks: n=459 (treatment initiators; Total ARNASA N=1375, initiated treatment: Jan 9, 2023-June 25, 2024; CT.Gov has no per-arm figure). Placebo: 457 (treatment initiators; the abstract does not use the word “randomized,” only “initiated treatment”; CT.Gov gives no per-arm breakdown, only an aggregate enrollment target of 1290, which is lower than and does not reconcile with the abstract's total of 1375 treatment initiators)	Any treatment-emergent adverse event. Outcome-specific timepoint NR; trial follow-up context: 52 weeks treatment period; primary endpoint (annualized rate of moderate/severe exacerbations) time_frame = “52 weeks”; all listed secondary endpoints (time to first exacerbation, SGRQ-C, FEV1, E-RS:COPD, annualized severe-exacerbation rate) time_frame = “52 weeks” or “week 52.” Abstract corroborates a 52-week treatment period.	Safety: TEAE, n/N per arm: Per-arm n/N is not reported in either source. Trial-total figure only: 1176/1375 (85.5%) of ARNASA participants (all 3 arms combined) had ≥1 adverse event, described as “balanced between treatments.” The most common non-COPD adverse event in ARNASA was “upper respiratory chest infection” (NOT “upper respiratory tract infection”: this exact wording is confirmed verbatim in the abstract and the alternate phrasing is a paraphrase drift that must not enter the workbook).	NR/not obtainable: no comparative effect with precision was present; see per-arm summary.	PMID 42150581	Not applicable: NR/not obtainable.	
	Active: astegolimab every 2 weeks: n=459. Astegolimab every 4 weeks: n=459 (treatment initiators; Total ARNASA N=1375, initiated treatment: Jan 9, 2023-June 25, 2024; CT.Gov has no per-arm figure). Placebo: 457 (treatment initiators; the abstract does not use the word “randomized,” only “initiated treatment”; CT.Gov gives no per-arm breakdown, only an aggregate enrollment target of 1290, which is lower than and does not reconcile with the abstract's total of 1375 treatment initiators)	Serious adverse events. Outcome-specific timepoint NR; trial follow-up context: 52 weeks treatment period; primary endpoint (annualized rate of moderate/severe exacerbations) time_frame = “52 weeks”; all listed secondary endpoints (time to first exacerbation, SGRQ-C, FEV1, E-RS:COPD, annualized severe-exacerbation rate) time_frame = “52 weeks” or “week 52.” Abstract corroborates a 52-week treatment period.	Safety: SAE, n/N per arm: Not obtainable: no SAE count (total or per-arm) appears in the Lancet abstract, and ClinicalTrials.gov has no results module posted (not obtainable)	NR/not obtainable: no comparative effect with precision was present; see per-arm summary.	NR/not obtainable: the source field is empty in the workbook.	Not applicable: NR/not obtainable.	
	Active: astegolimab every 2 weeks: n=459. Astegolimab every 4 weeks: n=459 (treatment initiators; Total ARNASA N=1375, initiated treatment: Jan 9, 2023-June 25, 2024; CT.Gov has no per-arm figure). Placebo: 457 (treatment initiators; the abstract does not use the word “randomized,” only “initiated treatment”; CT.Gov gives no per-arm breakdown, only an aggregate enrollment target of 1290, which is lower than and does not reconcile with the abstract's total of 1375 treatment initiators)	AEs leading to discontinuation. Outcome-specific timepoint NR; trial follow-up context: 52 weeks treatment period; primary endpoint (annualized rate of moderate/severe exacerbations) time_frame = “52 weeks”; all listed secondary endpoints (time to first exacerbation, SGRQ-C, FEV1, E-RS:COPD, annualized severe-exacerbation rate) time_frame = “52 weeks” or “week 52.” Abstract corroborates a 52-week treatment period.	Safety: discontinuation due to AE, n/N per arm: Not obtainable: not reported in the abstract; no results module on ClinicalTrials.gov (not obtainable)	NR/not obtainable: no comparative effect with precision was present; see per-arm summary.	NR/not obtainable: the source field is empty in the workbook.	Not applicable: NR/not obtainable.	
	Active: astegolimab every 2 weeks: n=459. Astegolimab every 4 weeks: n=459 (treatment initiators; Total ARNASA N=1375, initiated treatment: Jan 9, 2023-June 25, 2024; CT.Gov has no per-arm figure). Placebo: 457 (treatment initiators; the abstract does not use the word “randomized,” only “initiated treatment”; CT.Gov gives no per-arm breakdown, only an aggregate enrollment target of 1290, which is lower than and does not reconcile with the abstract's total of 1375 treatment initiators)	All-cause death. Outcome-specific timepoint NR; trial follow-up context: 52 weeks treatment period; primary endpoint (annualized rate of moderate/severe exacerbations) time_frame = “52 weeks”; all listed secondary endpoints (time to first exacerbation, SGRQ-C, FEV1, E-RS:COPD, annualized severe-exacerbation rate) time_frame = “52 weeks” or “week 52.” Abstract corroborates a 52-week treatment period.	Safety: death, n/N per arm: Per-arm n/N not reported in either source. Trial-total figure only: 44/1375 (3.2%) deaths in ARNASA, described as “balanced across treatment groups” in the abstract. Across ALIENTO+ARNASA combined (not ARNASA alone), 3 deaths total (0.1%) were judged treatment-related by investigators: pooled across both trials, not ARNASA-specific.	NR/not obtainable: no comparative effect with precision was present; see per-arm summary.	PMID 42150581	Not applicable: NR/not obtainable.	
	Active: astegolimab every 2 weeks: n=459. Astegolimab every 4 weeks: n=459 (treatment initiators; Total ARNASA N=1375, initiated treatment: Jan 9, 2023-June 25, 2024; CT.Gov has no per-arm figure). Placebo: 457 (treatment initiators; the abstract does not use the word “randomized,” only “initiated treatment”; CT.Gov gives no per-arm breakdown, only an aggregate enrollment target of 1290, which is lower than and does not reconcile with the abstract's total of 1375 treatment initiators)	AEs of special interest. Outcome-specific timepoint NR; trial follow-up context: 52 weeks treatment period; primary endpoint (annualized rate of moderate/severe exacerbations) time_frame = “52 weeks”; all listed secondary endpoints (time to first exacerbation, SGRQ-C, FEV1, E-RS:COPD, annualized severe-exacerbation rate) time_frame = “52 weeks” or “week 52.” Abstract corroborates a 52-week treatment period.	NR/not obtainable: no trial-specific numeric result for this domain was present in the extraction workbook.	NR/not obtainable: no trial-specific numeric result for this domain was present in the extraction workbook.	Extraction workbook (no matching trial-specific outcome row).	Not applicable: NR/not obtainable.	
S1.10 itepekimab PoC (NCT03546907) [37]							
itepekimab PoC	Active: itepekimab 300 mg SC: n=172 (placebo n=171; total randomized N=343), single active-dose arm. Placebo: 171	Moderate/severe exacerbation rate week 52	Exacerbation: adjusted annualized rate, active arm(s) (itepekimab 300 mg): itepekimab 300 mg: 1.30 (95% CI: 1.05-1.61) moderate-to-severe AECOPD per patient-year, baseline to week 52 (overall population); RR vs placebo 0.81 (95% CI: 0.61-1.07), p=0.13. Exacerbation: adjusted annualized rate, placebo arm: 1.61 (95% CI: 1.32-1.97) moderate-to-severe AECOPD per patient-year, baseline to week 52.	Eosinophil 150-<300: exacerbation RR vs placebo (95% CI): Not obtainable: same reason as the <150 eosinophil row; no such subgroup exists in either primary source (not obtainable).Eosinophil <150: exacerbation RR vs placebo (95% CI): Not obtainable: no eosinophil-stratified subgroup analysis exists in the CT.Gov record or PubMed abstract for this trial; the only prespecified/reported subgroup is by smoking status (not obtainable).Eosinophil ≥300: exacerbation RR vs placebo (95% CI): Not obtainable: same reason as the other eosinophil-threshold rows; no such subgroup exists in either primary source (not obtainable). Exacerbation: rate ratio vs placebo, 95% CI: RR 0.81 (95% CI: 0.61-1.07), p=0.13: overall population; primary endpoint not met. Smoking-status subgroup: exacerbation RR vs placebo (95% CI), current smokers: RR 1.09 (95% CI: 0.74-1.61), p=0.65: current smokers, prespecified subgroup analysis by smoking status; no exacerbation benefit observed. Smoking-status subgroup: exacerbation RR vs placebo (95% CI), former smokers: RR 0.58 (95% CI: 0.39-0.85), p=0.0061: former smokers, prespecified subgroup analysis by smoking status (no eosinophil-stratified subgroup exists for this trial)	ClinicalTrials.gov NCT03546907; PMID 34302758 abstract. PMID 34302758	No: transcribed as reported; S1 adds no calculation.	
	Active: itepekimab 300 mg SC: n=172 (placebo n=171; total randomized N=343), single active-dose arm. Placebo: 171	Pre-bronchodilator FEV1: weeks 16-24, weeks 16-24	FEV1: LS-mean change from baseline, active arm(s): itepekimab 300 mg LS-mean pre-BD FEV1 change, weeks 16-24: 0.06 L (spread 0.02). PubMed labels the parallel spread as SD; the registry labels both LS-mean spreads as SE, so the SD/SE source-label conflict remains explicit. FEV1: LS-mean change from baseline, placebo arm: placebo LS-mean pre-BD FEV1 change, weeks 16-24: 0.00 L (spread 0.02). PubMed explicitly labels 0.02 as SD; the registry labels it SE, so the source-label conflict remains explicit.	Eosinophil-subgroup FEV1 LS-mean difference vs placebo (any threshold): Not obtainable: no eosinophil-subgroup FEV1 result exists in the CT.Gov record or PubMed abstract; the trial’s only reported effect-modifier subgroup is smoking status (not obtainable). FEV1: LS-mean difference vs placebo (mL), SE/95% CI: Pre-BD LS-mean difference 0.06 L (60 mL), 95% CI: 0.01-0.10 L, P=0.024, weeks 16-24. Separately registered post-BD week-24 mean changes: placebo 0.01 L; itepekimab 0.04 L. Smoking-status subgroup: current smokers: FEV1 LS-mean difference vs placebo (95% CI): 0.02 L (20 mL) difference, 95% CI: -0.05 to 0.09 L, p=0.54: current smokers, pre-bronchodilator FEV1 weeks 16-24, prespecified subgroup by smoking status; no benefit observed. Same subgroup exacerbation RR 1.09 (95% CI: 0.74-1.61), p=0.65.Smoking-status subgroup: former smokers: FEV1 LS-mean difference vs placebo (95% CI): 0.09 L (90 mL) difference, 95% CI: 0.02-0.15 L (20-150 mL), p=0.0076: former smokers, pre-bronchodilator FEV1 weeks 16-24, prespecified subgroup by smoking status. Same subgroup also showed exacerbation RR 0.58 (95% CI: 0.39-0.85), p=0.0061.	ClinicalTrials.gov NCT03546907; PMID 34302758; PMID 34302758 abstract	No: transcribed as reported; S1 adds no calculation.	
	Active: itepekimab 300 mg SC: n=172 (placebo n=171; total randomized N=343), single active-dose arm. Placebo: 171	Post-bronchodilator FEV1: week 24	NR/not obtainable: no per-arm summary was present; see comparative/qualitative result.	FEV1: post-bronchodilator change at week 24 (registered secondary outcome): change from baseline in post-bronchodilator forced expiratory volume (FEV1) in 1 second at week 24; param=MEAN disp=liters: placebo=0.01; SAR440340=0.04	ClinicalTrials.gov NCT03546907	No: transcribed as reported; S1 adds no calculation.	
	Active: itepekimab 300 mg SC: n=172 (placebo n=171; total randomized N=343), single active-dose arm. Placebo: 171	SGRQ total score Outcome-specific timepoint NR; trial follow-up context: Treatment period 24-52 weeks (variable by enrollment timing); primary exacerbation endpoint assessed baseline to week 52; key secondary FEV1 endpoint assessed weeks 16-24; total study participation (up to 10 days-4 weeks screening + 24-52-week treatment + 20-week post-treatment follow-up) = 46-76 weeks.	SGRQ: LS-mean change from baseline, active arm(s): Not obtainable: same reason as placebo-arm SGRQ row (not obtainable).SGRQ: LS-mean change from baseline, placebo arm: Not obtainable: SGRQ is not a registered outcome measure for this trial per CT.Gov protocol record and is not mentioned in the PubMed abstract (not obtainable).	SGRQ: LS-mean difference vs placebo, SE/95% CI: Not obtainable: same reason as above (not obtainable).	ClinicalTrials.gov NCT03546907; PMID 34302758	Not applicable: NR/not obtainable.	
	Active: itepekimab 300 mg SC: n=172 (placebo n=171; total randomized N=343), single active-dose arm. Placebo: 171	E-RS:COPD. Outcome-specific timepoint NR; trial follow-up context: Treatment period 24-52 weeks (variable by enrollment timing); primary exacerbation endpoint assessed baseline to week 52; key secondary FEV1 endpoint assessed weeks 16-24; total study participation (up to 10 days-4 weeks screening + 24-52-week treatment + 20-week post-treatment follow-up) = 46-76 weeks.	E-RS: LS-mean change from baseline, active arm(s): Not obtainable: same reason as above (not obtainable).E-RS: LS-mean change from baseline, placebo arm: Not obtainable: E-RS is not a registered outcome measure for this trial per CT.Gov protocol record and is not mentioned in the PubMed abstract (not obtainable).	E-RS: LS-mean difference vs placebo, SE/95% CI: Not obtainable: same reason as above (not obtainable).	ClinicalTrials.gov NCT03546907; PMID 34302758	Not applicable: NR/not obtainable.	
	Active: itepekimab 300 mg SC: n=172 (placebo n=171; total randomized N=343), single active-dose arm. Placebo: 171	Time to first exacerbation. Outcome-specific timepoint NR; trial follow-up context: Treatment period 24-52 weeks (variable by enrollment timing); primary exacerbation endpoint assessed baseline to week 52; key secondary FEV1 endpoint assessed weeks 16-24; total study participation (up to 10 days-4 weeks screening + 24-52-week treatment + 20-week post-treatment follow-up) = 46-76 weeks.	NR/not obtainable: no trial-specific numeric result for this domain was present in the extraction workbook.	NR/not obtainable: no trial-specific numeric result for this domain was present in the extraction workbook.	Extraction workbook (no matching trial-specific outcome row).	Not applicable: NR/not obtainable.	
	Active: itepekimab 300 mg SC: n=172 (placebo n=171; total randomized N=343), single active-dose arm. Placebo: 171	Rescue-medication use. Outcome-specific timepoint NR; trial follow-up context: Treatment period 24-52 weeks (variable by enrollment timing); primary exacerbation endpoint assessed baseline to week 52; key secondary FEV1 endpoint assessed weeks 16-24; total study participation (up to 10 days-4 weeks screening + 24-52-week treatment + 20-week post-treatment follow-up) = 46-76 weeks.	NR/not obtainable: no trial-specific numeric result for this domain was present in the extraction workbook.	NR/not obtainable: no trial-specific numeric result for this domain was present in the extraction workbook.	Extraction workbook (no matching trial-specific outcome row).	Not applicable: NR/not obtainable.	
	Active: itepekimab 300 mg SC: n=172 (placebo n=171; total randomized N=343), single active-dose arm. Placebo: 171	Any treatment-emergent adverse event. Outcome-specific timepoint NR; trial follow-up context: Treatment period 24-52 weeks (variable by enrollment timing); primary exacerbation endpoint assessed baseline to week 52; key secondary FEV1 endpoint assessed weeks 16-24; total study participation (up to 10 days-4 weeks screening + 24-52-week treatment + 20-week post-treatment follow-up) = 46-76 weeks.	Safety: TEAE, n/N per arm: Any TEAE: itepekimab 135/172 (78%) vs Placebo 136/171 (80%). Most common TEAEs: nasopharyngitis (itepekimab 28/172 (16%) vs placebo 29/171 (17%)), bronchitis (18/172 (10%) vs 14/171 (8%)), headache (14/172 (8%) vs 23/171 (13%)), upper respiratory tract infection (13/172 (8%) vs 15/171 (9%))	NR/not obtainable: no comparative effect with precision was present; see per-arm summary.	PMID 34302758	No: transcribed as reported; S1 adds no calculation.	
	Active: itepekimab 300 mg SC: n=172 (placebo n=171; total randomized N=343), single active-dose arm. Placebo: 171	Serious adverse events. Outcome-specific timepoint NR; trial follow-up context: Treatment period 24-52 weeks (variable by enrollment timing); primary exacerbation endpoint assessed baseline to week 52; key secondary FEV1 endpoint assessed weeks 16-24; total study participation (up to 10 days-4 weeks screening + 24-52-week treatment + 20-week post-treatment follow-up) = 46-76 weeks.	Safety: SAE, n/N per arm: placebo: serious. Affected=36/171; SAR440340: serious. Affected=29/172	NR/not obtainable: no comparative effect with precision was present; see per-arm summary.	ClinicalTrials.gov NCT03546907	No: transcribed as reported; S1 adds no calculation.	
	Active: itepekimab 300 mg SC: n=172 (placebo n=171; total randomized N=343), single active-dose arm. Placebo: 171	AEs leading to discontinuation. Outcome-specific timepoint NR; trial follow-up context: Treatment period 24-52 weeks (variable by enrollment timing); primary exacerbation endpoint assessed baseline to week 52; key secondary FEV1 endpoint assessed weeks 16-24; total study participation (up to 10 days-4 weeks screening + 24-52-week treatment + 20-week post-treatment follow-up) = 46-76 weeks.	Safety: discontinuation due to AE, n/N per arm: placebo 7/171 started; itepekimab 10/172 started; withdrew because of an adverse event.	NR/not obtainable: no comparative effect with precision was present; see per-arm summary.	ClinicalTrials.gov NCT03546907	No: transcribed as reported; S1 adds no calculation.	
	Active: itepekimab 300 mg SC: n=172 (placebo n=171; total randomized N=343), single active-dose arm. Placebo: 171	All-cause death. Outcome-specific timepoint NR; trial follow-up context: Treatment period 24-52 weeks (variable by enrollment timing); primary exacerbation endpoint assessed baseline to week 52; key secondary FEV1 endpoint assessed weeks 16-24; total study participation (up to 10 days-4 weeks screening + 24-52-week treatment + 20-week post-treatment follow-up) = 46-76 weeks.	Safety: death, n/N per arm: placebo: deaths=2/171; SAR440340: deaths=3/172	NR/not obtainable: no comparative effect with precision was present; see per-arm summary.	ClinicalTrials.gov NCT03546907	No: transcribed as reported; S1 adds no calculation.	
	Active: itepekimab 300 mg SC: n=172 (placebo n=171; total randomized N=343), single active-dose arm. Placebo: 171	AEs of special interest. Outcome-specific timepoint NR; trial follow-up context: Treatment period 24-52 weeks (variable by enrollment timing); primary exacerbation endpoint assessed baseline to week 52; key secondary FEV1 endpoint assessed weeks 16-24; total study participation (up to 10 days-4 weeks screening + 24-52-week treatment + 20-week post-treatment follow-up) = 46-76 weeks.	NR/not obtainable: no trial-specific numeric result for this domain was present in the extraction workbook.	NR/not obtainable: no trial-specific numeric result for this domain was present in the extraction workbook.	Extraction workbook (no matching trial-specific outcome row).	Not applicable: NR/not obtainable.	
S1.11 FRONTIER-4 (NCT04631016) [19]							
FRONTIER-4	Active: 69 randomized to tozorakimab 600 mg (Figure 1 legend, total randomized N=137); 2 randomized tozorakimab patients excluded post-randomization; ITT/efficacy/safety population; tozorakimab n=67 (used throughout Tables 1, 2, 4). Same 135-vs-1. Placebo: 68 randomized to placebo (total randomized N=137: tozorakimab 69/placebo 68, per Figure 1 legend). ITT population placebo n=68 (no placebo-arm patients excluded from ITT). Note: the article’s results narrative states “Of 466 patients screened, 135 were randomized, comprising the ITT population (tozorakimab, n=67; placebo, n=68)”: this sentence conflates the randomized total with the ITT total; Figure 1 and Table 1 correctly and consistently distinguish 137 randomized vs 135 ITT.	Moderate/severe exacerbation rate. Outcome-specific timepoint NR; trial follow-up context: Minimum 4-week screening/run-in period; 28-week intervention period (dosing q4w for 24 weeks, primary endpoint at week 12, end-of-treatment analysis at week 28); plus 8 weeks safety follow-up after last dose. CT.Gov secondary-outcome time_frame fields specify "Day 1 through 253 days (maximum observed duration)," i.e., ~36 weeks total observation.	Exacerbation: adjusted annualized rate, active arm(s): Not applicable/not obtainable: same reason as placebo-arm row (not obtainable). Exacerbation: adjusted annualized rate, placebo arm: Not applicable/not obtainable: FRONTIER-4 did not use an annualized exacerbation rate design; endpoint was time-to-first COPDCompEx composite event (Cox proportional-hazards model) (not obtainable).	Exacerbation: rate ratio vs placebo, 95% CI: Not applicable: FRONTIER-4 did not report a true annualized exacerbation rate ratio. Closest available analog, ITT population: time-to-first COPDCompEx event, Cox HR 0.79 (80% CI 0.57-1.11), p=0.186; events 28/67 (41.8%) tozorakimab vs 36/68 (52.9%) placebo. Reported at an 80% CI (not 95%) per this signal-finding trial’s prespecified one-sided 10% significance threshold: must be handled as a hazard-ratio proxy, not pooled directly with annualized-rate-ratio trials in the NMA.	CT.Gov secondary_outcomes; fulltext MethodsCT.Gov secondary_outcomes + Europe PMC full text Methods ‘Statistical analysis’PMC12256803	No: transcribed as reported; S1 adds no calculation.	
	Active: 69 randomized to tozorakimab 600 mg (Figure 1 legend, total randomized N=137); 2 randomized tozorakimab patients excluded post-randomization; ITT/efficacy/safety population; tozorakimab n=67 (used throughout Tables 1, 2, 4). Same 135-vs-1. Placebo: 68 randomized to placebo (total randomized N=137: tozorakimab 69/placebo 68, per Figure 1 legend). ITT population placebo n=68 (no placebo-arm patients excluded from ITT). Note: the article’s results narrative states “Of 466 patients screened, 135 were randomized, comprising the ITT population (tozorakimab, n=67; placebo, n=68)”: this sentence conflates the randomized total with the ITT total; Figure 1 and Table 1 correctly and consistently distinguish 137 randomized vs 135 ITT.	Pre-bronchodilator FEV1: week 12	FEV1 (pre-bronchodilator, primary endpoint): LS-mean change from baseline, active arm(s): +19±26 mL (mean±SE), pre-BD FEV1 change from baseline to week 12, ITT population, tozorakimab 600 mg n=67. FEV1 (pre-bronchodilator, primary endpoint): LS-mean change from baseline, placebo arm: −5±25 mL (mean±SE), pre-BD FEV1 change from baseline to week 12, ITT population, placebo n=68.	Eosinophil <150 cells/µL: Pre-BD FEV1 LS-mean change and difference vs placebo (80% CI): Tozorakimab (n=37): −12±32 mL. Placebo (n=36): +12±31 mL. LSMD −25 mL, 80% CI −77 to 28 mL, p=0.275 (not significant; numerically favors placebo in this subgroup). Eosinophil ≥150 cells/µL: Pre-BD FEV1 LS-mean change and difference vs placebo (80% CI): Tozorakimab (n=30): +57±35 mL. Placebo (n=32): −25±35 mL. LSMD 82 mL, 80% CI 26-138 mL, p=0.031 (statistically significant at the trial’s 10% one-sided/80%-CI threshold). FEV1 (pre-bronchodilator, primary endpoint): LS-mean difference vs placebo (mL), SE/CI: LSMD 24 mL, 80% CI −15 to 63 mL, p=0.216: primary endpoint NOT statistically significant. 80% CI per the prespecified one-sided 10% significance threshold; no separate SE reported for the difference estimate itself.	PMC12256803	No: transcribed as reported; S1 adds no calculation.	
	Active: 69 randomized to tozorakimab 600 mg (Figure 1 legend, total randomized N=137); 2 randomized tozorakimab patients excluded post-randomization; ITT/efficacy/safety population; tozorakimab n=67 (used throughout Tables 1, 2, 4). Same 135-vs-1. Placebo: 68 randomized to placebo (total randomized N=137: tozorakimab 69/placebo 68, per Figure 1 legend). ITT population placebo n=68 (no placebo-arm patients excluded from ITT). Note: the article’s results narrative states “Of 466 patients screened, 135 were randomized, comprising the ITT population (tozorakimab, n=67; placebo, n=68)”: this sentence conflates the randomized total with the ITT total; Figure 1 and Table 1 correctly and consistently distinguish 137 randomized vs 135 ITT.	Post-bronchodilator FEV1: week 12, week 28	FEV1 (post-bronchodilator, key secondary): LS-mean change from baseline, active arm(s): +41±36 mL (mean±SE), post-BD FEV1 change from baseline to week 12, ITT population, tozorakimab n=67. FEV1 (post-bronchodilator, key secondary): LS-mean change from baseline, placebo arm: −25±34 mL (mean±SE), post-BD FEV1 change from baseline to week 12, ITT population, placebo n=68.	Eosinophil <150 cells/µL: Post-BD FEV1 LS-mean change and difference vs placebo (80% CI): Tozorakimab (n=37): +5±42 mL. Placebo (n=36): +8±41 mL. LSMD −3 mL, 80% CI −71 to 64 mL, p=0.474 (not significant).Eosinophil ≥150 cells/µL: Post-BD FEV1 LS-mean change and difference vs placebo (80% CI): Tozorakimab (n=30): +97±46 mL. Placebo (n=32): −59±45 mL. LSMD 155 mL, 80% CI 84-227 mL, p=0.003 (statistically significant; largest effect size of any subgroup reported). FEV1 (post-bronchodilator, key secondary): LS-mean difference vs placebo, 80% CI: week 12: LSMD 67 mL, 80% CI 17-116 mL, p=0.044 (statistically significant at the trial’s prespecified 10% one-sided threshold). Sustained to week 28: LSMD 70 mL, 80% CI 20-120 mL, p=0.036.Prespecified subgroup (≥2 AECOPD in prior 12 months): pre-BD/post-BD FEV1 LSMD and COPDCompEx HR vs placebo: Pre-BD FEV1: tozorakimab n=26 +61±38 mL vs placebo n=33 −8±34 mL, LSMD +69 mL (80% CI 9-130 mL), p=0.072. Post-BD FEV1: tozorakimab +103±50 mL vs placebo −21±44 mL, LSMD +124 mL (80% CI 47-201 mL), p=0.020. COPDCompEx: tozorakimab 11/26 (42.3%) vs placebo 22/33 (66.7%) with event, HR 0.61 (80% CI 0.37-1.00), p=0.098.	PMC12256803	No: transcribed as reported; S1 adds no calculation.	
	Active: 69 randomized to tozorakimab 600 mg (Figure 1 legend, total randomized N=137); 2 randomized tozorakimab patients excluded post-randomization; ITT/efficacy/safety population; tozorakimab n=67 (used throughout Tables 1, 2, 4). Same 135-vs-1. Placebo: 68 randomized to placebo (total randomized N=137: tozorakimab 69/placebo 68, per Figure 1 legend). ITT population placebo n=68 (no placebo-arm patients excluded from ITT). Note: the article’s results narrative states “Of 466 patients screened, 135 were randomized, comprising the ITT population (tozorakimab, n=67; placebo, n=68)”: this sentence conflates the randomized total with the ITT total; Figure 1 and Table 1 correctly and consistently distinguish 137 randomized vs 135 ITT.	SGRQ total score week 12	SGRQ: LS-mean change from baseline, active arm(s): change from baseline to week 12 in Saint George’s Respiratory Questionnaire (SGRQ) Total Score: param=LEAST_SQUARES_MEAN disp=Units on a scale: Tozorakimab=-9.177SGRQ: LS-mean change from baseline, placebo arm: change from Baseline bo week 12 in Saint George’s Respiratory Questionnaire (SGRQ) Total Score: param=LEAST_SQUARES_MEAN disp=Units on a scale: placebo=-8.273	SGRQ: LS-mean difference vs placebo, SE/95% CI: Not obtainable: reported only narratively as “not statistically different between treatment groups”; no numeric LSMD, SE, or CI in retrieved main text (supplement not accessed) (not obtainable).	ClinicalTrials.gov NCT04631016	No: transcribed as reported; S1 adds no calculation.	
	Active: 69 randomized to tozorakimab 600 mg (Figure 1 legend, total randomized N=137); 2 randomized tozorakimab patients excluded post-randomization; ITT/efficacy/safety population; tozorakimab n=67 (used throughout Tables 1, 2, 4). Same 135-vs-1. Placebo: 68 randomized to placebo (total randomized N=137: tozorakimab 69/placebo 68, per Figure 1 legend). ITT population placebo n=68 (no placebo-arm patients excluded from ITT). Note: the article’s results narrative states “Of 466 patients screened, 135 were randomized, comprising the ITT population (tozorakimab, n=67; placebo, n=68)”: this sentence conflates the randomized total with the ITT total; Figure 1 and Table 1 correctly and consistently distinguish 137 randomized vs 135 ITT.	E-RS:COPD week 12	E-RS: LS-mean change from baseline, active arm(s): change from baseline to week 12 in 4-weekly Mean Evaluating Respiratory Symptoms of COPD (E-RS:COPD). Total score: param=LEAST_SQUARES_MEAN disp=Units on a scale: Tozorakimab=-6.09E-RS: LS-mean change from baseline, placebo arm: change from baseline to week 12 in 4-weekly Mean Evaluating Respiratory Symptoms of COPD (E-RS:COPD) Total Score: param=LEAST_SQUARES_MEAN disp=Units on a scale: placebo=-6.06	E-RS: LS-mean difference vs placebo, SE/95% CI: Not obtainable: narrative-only statement; no numeric LSMD, SE, or CI given in retrieved main text (not obtainable).	ClinicalTrials.gov NCT04631016	No: transcribed as reported; S1 adds no calculation.	
	Active: 69 randomized to tozorakimab 600 mg (Figure 1 legend, total randomized N=137); 2 randomized tozorakimab patients excluded post-randomization; ITT/efficacy/safety population; tozorakimab n=67 (used throughout Tables 1, 2, 4). Same 135-vs-1. Placebo: 68 randomized to placebo (total randomized N=137: tozorakimab 69/placebo 68, per Figure 1 legend). ITT population placebo n=68 (no placebo-arm patients excluded from ITT). Note: the article’s results narrative states “Of 466 patients screened, 135 were randomized, comprising the ITT population (tozorakimab, n=67; placebo, n=68)”: this sentence conflates the randomized total with the ITT total; Figure 1 and Table 1 correctly and consistently distinguish 137 randomized vs 135 ITT.	Time to first exacerbation. Outcome-specific timepoint NR; trial follow-up context: Minimum 4-week screening/run-in period; 28-week intervention period (dosing q4w for 24 weeks, primary endpoint at week 12, end-of-treatment analysis at week 28); plus 8 weeks safety follow-up after last dose. CT.Gov secondary-outcome time_frame fields specify 'Day 1 through 253 days (maximum observed duration)', i.e., ~36 weeks total observation.	NR/not obtainable: no per-arm summary was present; see comparative/qualitative result.	COPDCompEx time-to-first-event hazard ratio vs placebo, ITT population (dedicated row; note 80% CI not 95%): HR 0.79 (80% CI 0.57-1.11), p=0.186, not statistically significant. Events: tozorakimab 28/67 (41.8%), placebo 36/68 (52.9%). Exacerbation: COPDCompEx hazard ratio vs placebo (80% CI), ITT population: HR 0.79 (80% CI 0.57-1.11), p=0.186; events 28/67 (41.8%) tozorakimab vs 36/68 (52.9%) placebo.	PMC12256803	No: transcribed as reported; S1 adds no calculation.	
	Active: 69 randomized to tozorakimab 600 mg (Figure 1 legend, total randomized N=137); 2 randomized tozorakimab patients excluded post-randomization; ITT/efficacy/safety population; tozorakimab n=67 (used throughout Tables 1, 2, 4). Same 135-vs-1. Placebo: 68 randomized to placebo (total randomized N=137: tozorakimab 69/placebo 68, per Figure 1 legend). ITT population placebo n=68 (no placebo-arm patients excluded from ITT). Note: the article’s results narrative states “Of 466 patients screened, 135 were randomized, comprising the ITT population (tozorakimab, n=67; placebo, n=68)”: this sentence conflates the randomized total with the ITT total; Figure 1 and Table 1 correctly and consistently distinguish 137 randomized vs 135 ITT.	Rescue-medication use. Outcome-specific timepoint NR; trial follow-up context: Minimum 4-week screening/run-in period; 28-week intervention period (dosing q4w for 24 weeks, primary endpoint at week 12, end-of-treatment analysis at week 28); plus 8 weeks safety follow-up after last dose. CT.Gov secondary-outcome time_frame fields specify 'Day 1 through 253 days (maximum observed duration)', i.e., ~36 weeks total observation.	NR/not obtainable: no trial-specific numeric result for this domain was present in the extraction workbook.	NR/not obtainable: no trial-specific numeric result for this domain was present in the extraction workbook.	Extraction workbook (no matching trial-specific outcome row).	Not applicable: NR/not obtainable.	
	Active: 69 randomized to tozorakimab 600 mg (Figure 1 legend, total randomized N=137); 2 randomized tozorakimab patients excluded post-randomization; ITT/efficacy/safety population; tozorakimab n=67 (used throughout Tables 1, 2, 4). Same 135-vs-1. Placebo: 68 randomized to placebo (total randomized N=137: tozorakimab 69/placebo 68, per Figure 1 legend). ITT population placebo n=68 (no placebo-arm patients excluded from ITT). Note: the article’s results narrative states “Of 466 patients screened, 135 were randomized, comprising the ITT population (tozorakimab, n=67; placebo, n=68)”: this sentence conflates the randomized total with the ITT total; Figure 1 and Table 1 correctly and consistently distinguish 137 randomized vs 135 ITT.	Any treatment-emergent adverse event. Outcome-specific timepoint NR; trial follow-up context: Minimum 4-week screening/run-in period; 28-week intervention period (dosing q4w for 24 weeks, primary endpoint at week 12, end-of-treatment analysis at week 28); plus 8 weeks safety follow-up after last dose. CT.Gov secondary-outcome time_frame fields specify 'Day 1 through 253 days (maximum observed duration)', i.e., ~36 weeks total observation.	Safety: TEAE, n/N per arm: Any TEAE: placebo 50/68 (73.5%); Tozorakimab 53/67 (79.1%).	NR/not obtainable: no comparative effect with precision was present; see per-arm summary.	Europe PMC full text, Table 4	No: transcribed as reported; S1 adds no calculation.	
	Active: 69 randomized to tozorakimab 600 mg (Figure 1 legend, total randomized N=137); 2 randomized tozorakimab patients excluded post-randomization; ITT/efficacy/safety population; tozorakimab n=67 (used throughout Tables 1, 2, 4). Same 135-vs-1. Placebo: 68 randomized to placebo (total randomized N=137: tozorakimab 69/placebo 68, per Figure 1 legend). ITT population placebo n=68 (no placebo-arm patients excluded from ITT). Note: the article’s results narrative states “Of 466 patients screened, 135 were randomized, comprising the ITT population (tozorakimab, n=67; placebo, n=68)”: this sentence conflates the randomized total with the ITT total; Figure 1 and Table 1 correctly and consistently distinguish 137 randomized vs 135 ITT.	Serious adverse events. Outcome-specific timepoint NR; trial follow-up context: Minimum 4-week screening/run-in period; 28-week intervention period (dosing q4w for 24 weeks, primary endpoint at week 12, end-of-treatment analysis at week 28); plus 8 weeks safety follow-up after last dose. CT.Gov secondary-outcome time_frame fields specify 'Day 1 through 253 days (maximum observed duration)', i.e., ~36 weeks total observation.	Safety: SAE (including events with outcome of death), n/N per arm: Tozorakimab 14/67 (20.9%); placebo 9/68 (13.2%).Safety: SAE, n/N per arm: Any SAE (including events with the outcome of death): placebo 9/68 (13.2%); Tozorakimab 14/67 (20.9%). No life-threatening or fatal TEAEs in either arm (0/68, 0/67).	NR/not obtainable: no comparative effect with precision was present; see per-arm summary.	PMC12256803	No: transcribed as reported; S1 adds no calculation.	
	Active: 69 randomized to tozorakimab 600 mg (Figure 1 legend, total randomized N=137); 2 randomized tozorakimab patients excluded post-randomization; ITT/efficacy/safety population; tozorakimab n=67 (used throughout Tables 1, 2, 4). Same 135-vs-1. Placebo: 68 randomized to placebo (total randomized N=137: tozorakimab 69/placebo 68, per Figure 1 legend). ITT population placebo n=68 (no placebo-arm patients excluded from ITT). Note: the article’s results narrative states “Of 466 patients screened, 135 were randomized, comprising the ITT population (tozorakimab, n=67; placebo, n=68)”: this sentence conflates the randomized total with the ITT total; Figure 1 and Table 1 correctly and consistently distinguish 137 randomized vs 135 ITT.	AEs leading to discontinuation. Outcome-specific timepoint NR; trial follow-up context: Minimum 4-week screening/run-in period; 28-week intervention period (dosing q4w for 24 weeks, primary endpoint at week 12, end-of-treatment analysis at week 28); plus 8 weeks safety follow-up after last dose. CT.Gov secondary-outcome time_frame fields specify 'Day 1 through 253 days (maximum observed duration)', i.e., ~36 weeks total observation.	Safety: discontinuation due to AE, n/N per arm: TEAE leading to discontinuation of investigational product: placebo 0/68 (0.0%); Tozorakimab 4/67 (6.0%). TEAE leading to withdrawal from study (identical counts): placebo 0/68 (0.0%); Tozorakimab 4/67 (6.0%).	NR/not obtainable: no comparative effect with precision was present; see per-arm summary.	Europe PMC full text, Table 4	No: transcribed as reported; S1 adds no calculation.	
	Active: 69 randomized to tozorakimab 600 mg (Figure 1 legend, total randomized N=137); 2 randomized tozorakimab patients excluded post-randomization; ITT/efficacy/safety population; tozorakimab n=67 (used throughout Tables 1, 2, 4). Same 135-vs-1. Placebo: 68 randomized to placebo (total randomized N=137: tozorakimab 69/placebo 68, per Figure 1 legend). ITT population placebo n=68 (no placebo-arm patients excluded from ITT). Note: the article’s results narrative states “Of 466 patients screened, 135 were randomized, comprising the ITT population (tozorakimab, n=67; placebo, n=68)”: this sentence conflates the randomized total with the ITT total; Figure 1 and Table 1 correctly and consistently distinguish 137 randomized vs 135 ITT.	All-cause death. Outcome-specific timepoint NR; trial follow-up context: Minimum 4-week screening/run-in period; 28-week intervention period (dosing q4w for 24 weeks, primary endpoint at week 12, end-of-treatment analysis at week 28); plus 8 weeks safety follow-up after last dose. CT.Gov secondary-outcome time_frame fields specify 'Day 1 through 253 days (maximum observed duration)', i.e., ~36 weeks total observation.	Safety: death, n/N per arm: Any TEAE with the outcome of death: placebo 0/68 (0.0%); Tozorakimab 0/67 (0.0%). No life-threatening or fatal TEAEs reported in either arm.	NR/not obtainable: no comparative effect with precision was present; see per-arm summary.	Europe PMC full text, Table 4	No: transcribed as reported; S1 adds no calculation.	
	Active: 69 randomized to tozorakimab 600 mg (Figure 1 legend, total randomized N=137); 2 randomized tozorakimab patients excluded post-randomization; ITT/efficacy/safety population; tozorakimab n=67 (used throughout Tables 1, 2, 4). Same 135-vs-1. Placebo: 68 randomized to placebo (total randomized N=137: tozorakimab 69/placebo 68, per Figure 1 legend). ITT population placebo n=68 (no placebo-arm patients excluded from ITT). Note: the article’s results narrative states “Of 466 patients screened, 135 were randomized, comprising the ITT population (tozorakimab, n=67; placebo, n=68)”: this sentence conflates the randomized total with the ITT total; Figure 1 and Table 1 correctly and consistently distinguish 137 randomized vs 135 ITT.	AEs of special interest. Outcome-specific timepoint NR; trial follow-up context: Minimum 4-week screening/run-in period; 28-week intervention period (dosing q4w for 24 weeks, primary endpoint at week 12, end-of-treatment analysis at week 28); plus 8 weeks safety follow-up after last dose. CT.Gov secondary-outcome time_frame fields specify “Day 1 through 253 days (maximum observed duration)”, i.e., ~36 weeks total observation.	Safety: AESI (adverse event of special interest), n/N per arm: Table 4 (“Any AESI,” authoritative): placebo 24/68 (35.3%); Tozorakimab 33/67 (49.3%). The article’s Results narrative text states different, non-matching figures for the same nominal endpoint (“a higher proportion of patients experienced at least one TEAE of special interest in the tozorakimab group than in the placebo group (43.3% vs 29.4%, respectively)”): an unresolved internal inconsistency within the published article itself (Table 4 vs narrative), not an extraction artifact. Table 4 values are used as authoritative.	NR/not obtainable: no comparative effect with precision was present; see per-arm summary.	PMC12256803	No: transcribed as reported; S1 adds no calculation.	
S1.12 COPD-ST2OP (NCT03615040) [38]							
COPD-ST2OP	Active: STARTED: Anti-ST2=42 (baseline denom anti-ST2=42; total=81). Placebo: STARTED: placebo=39 (baseline denom. Placebo=39; total=81)	Moderate/severe exacerbation rate0-48 weeks	Exacerbation: adjusted annualized rate, active arm(s): 2.18 exacerbations/year (95% CI: 1.59-2.78), astegolimab 490 mg SC q4w, ITT population, 0-48 weeks, negative binomial count model. Exacerbation: adjusted annualized rate, placebo arm: 2.81 exacerbations/year (95% CI: 2.05-3.58), ITT population, 0-48 weeks, negative binomial count model. Note: eosinophil-subgroup absolute exacerbation rates per arm (not just rate ratios): Not obtainable: the Lancet Respir Med abstract reports only one summary figure per eosinophil subgroup (0.69 for ≤170; 0.83 for >170), not the separate absolute annualized exacerbation rate for the placebo arm and the astegolimab arm within each subgroup. No PMCID/open-access full text exists to retrieve a per-arm breakdown (not obtainable).	Eosinophil >170 cells/µL: exacerbation figure vs placebo (95% CI), prespecified subgroup: 0.83 (95% CI: 0.49-1.40) for the baseline blood eosinophil >170 cells/µL prespecified subgroup. Same rate-ratio inference and same residual unit-labeling ambiguity as the ≤170 stratum. Eosinophil ≤170 cells/µL: exacerbation figure vs placebo (95% CI), prespecified subgroup: 0.69 (95% CI: 0.39-1.21) for the baseline blood eosinophil ≤170 cells/µL prespecified subgroup. Value/CI confirmed digit-for-digit. Unit/interpretation genuinely ambiguous in the primary source: the abstract sentence “patients with 170 or fewer cells per µL had 0.69 exacerbations (0.39 to 1.21)” never restates “rate ratio,” though the parallel sentence structure and the magnitude (well below both arms’ absolute ITT rates of 2.18-2.81/yr) strongly suggest it is the astegolimab-vs-placebo rate ratio within that subgroup, analogous to the overall 0.78 rate ratio. Exacerbation: rate ratio vs placebo, 95% CI: Rate ratio 0.78 (95% CI: 0.53-1.14); p=0.19 (not statistically significant).	PubMed PMID 35339234	No: transcribed as reported; S1 adds no calculation.	
	Active: STARTED: Anti-ST2=42 (baseline denom anti-ST2=42; total=81). Placebo: STARTED: placebo=39 (baseline denom. Placebo=39; total=81)	Pre-bronchodilator FEV1. Outcome-specific timepoint NR; trial follow-up context: 48-week randomized treatment period plus 12-week follow-up = 60 weeks total observation; primary exacerbation endpoint assessed 0-48 weeks; safety assessed through week 60	FEV1: LS-mean change from baseline, active arm(s): Not obtainable: the raw registry reports outcome levels and a between-group analysis, but not the requested active-arm LS-mean change from baseline (not obtainable)FEV1: LS-mean change from baseline, placebo arm: Not obtainable: the raw registry and abstract report levels/contrast, but not the requested placebo-arm LS-mean change from baseline (not obtainable)	Eosinophil subgroup: SGRQ or FEV1 stratified estimates (by baseline blood eosinophil count): Not obtainable: CT.Gov registers SGRQ-C and FEV1 as subgroup objectives by baseline blood eosinophil count (design-level entries only, no numeric results), but neither the abstract nor the CT.Gov record gives a numeric subgroup breakdown for SGRQ or FEV1 (only for exacerbation rate); full text/supplement inaccessible (not obtainable). FEV1: LS-mean difference vs placebo (mL), SE/95% CI: Mean difference +40 mL (astegolimab vs placebo), 95% CI: −10 to 90 mL; p=0.094 (not statistically significant). SE not reported in the abstract.	ClinicalTrials.gov NCT03615040PubMed abstract, PubMed PMID 35339234	Not applicable: NR/not obtainable.	
	Active: STARTED: Anti-ST2=42 (baseline denom anti-ST2=42; total=81). Placebo: STARTED: placebo=39 (baseline denom. Placebo=39; total=81)	Post-bronchodilator FEV1. Outcome-specific timepoint NR; trial follow-up context: 48-week randomized treatment period plus 12-week follow-up = 60 weeks total observation; primary exacerbation endpoint assessed 0-48 weeks; safety assessed through week 60	NR/not obtainable: no trial-specific numeric result for this domain was present in the extraction workbook.	NR/not obtainable: no trial-specific numeric result for this domain was present in the extraction workbook.	Extraction workbook (no matching trial-specific outcome row).	Not applicable: NR/not obtainable.	
	Active: STARTED: Anti-ST2=42 (baseline denom anti-ST2=42; total=81). Placebo: STARTED: placebo=39 (baseline denom. Placebo=39; total=81)	SGRQ total score Outcome-specific timepoint NR; trial follow-up context: 48-week randomized treatment period plus 12-week follow-up = 60 weeks total observation; primary exacerbation endpoint assessed 0-48 weeks; safety assessed through week 60	SGRQ: LS-mean change from baseline, active arm(s): Not obtainable: the raw registry reports outcome levels and a between-group analysis, but not the requested active-arm LS-mean change from baseline (not obtainable)SGRQ: LS-mean change from baseline, placebo arm: Not obtainable: the raw registry and abstract report levels/contrast, but not the requested placebo-arm LS-mean change from baseline (not obtainable)	SGRQ: LS-mean difference vs placebo (SGRQ-C), SE/95% CI: SGRQ-C mean difference −3.3 (astegolimab vs placebo), 95% CI: −6.4 to −0.2; p=0.039, statistically significant improvement with astegolimab.	ClinicalTrials.gov NCT03615040PubMed PMID 35339234	No: transcribed as reported; S1 adds no calculation.	
	Active: STARTED: Anti-ST2=42 (baseline denom anti-ST2=42; total=81). Placebo: STARTED: placebo=39 (baseline denom. Placebo=39; total=81)	E-RS:COPD. Outcome-specific timepoint NR; trial follow-up context: 48-week randomized treatment period plus 12-week follow-up = 60 weeks total observation; primary exacerbation endpoint assessed 0-48 weeks; safety assessed through week 60	E-RS: LS-mean change from baseline, active arm(s): Not obtainable: same reason; E-RS not among this trial’s outcome measures (not obtainable)E-RS: LS-mean change from baseline, placebo arm: Not obtainable: this trial did not use the E-RS instrument; CT.Gov secondary/PRO outcomes list SGRQ-C, CAT, mMRC, and VAS dyspnea/cough/sputum, but no E-RS measure, and the abstract does not mention E-RS (not obtainable)	E-RS: LS-mean difference vs placebo, SE/95% CI: Not obtainable: same reason; E-RS not measured in COPD-ST2OP (trial used SGRQ-C/CAT/mMRC/VAS instead) (not obtainable)	ClinicalTrials.gov NCT03615040	Not applicable: NR/not obtainable.	
	Active: STARTED: Anti-ST2=42 (baseline denom anti-ST2=42; total=81). Placebo: STARTED: placebo=39 (baseline denom. Placebo=39; total=81)	Time to first exacerbation. Outcome-specific timepoint NR; trial follow-up context: 48-week randomized treatment period plus 12-week follow-up = 60 weeks total observation; primary exacerbation endpoint assessed 0-48 weeks; safety assessed through week 60	NR/not obtainable: no trial-specific numeric result for this domain was present in the extraction workbook.	NR/not obtainable: no trial-specific numeric result for this domain was present in the extraction workbook.	Extraction workbook (no matching trial-specific outcome row).	Not applicable: NR/not obtainable.	
	Active: STARTED: Anti-ST2=42 (baseline denom anti-ST2=42; total=81). Placebo: STARTED: placebo=39 (baseline denom. Placebo=39; total=81)	Rescue-medication use. Outcome-specific timepoint NR; trial follow-up context: 48-week randomized treatment period plus 12-week follow-up = 60 weeks total observation; primary exacerbation endpoint assessed 0-48 weeks; safety assessed through week 60	NR/not obtainable: no trial-specific numeric result for this domain was present in the extraction workbook.	NR/not obtainable: no trial-specific numeric result for this domain was present in the extraction workbook.	Extraction workbook (no matching trial-specific outcome row).	Not applicable: NR/not obtainable.	
	Active: STARTED: Anti-ST2=42 (baseline denom anti-ST2=42; total=81). Placebo: STARTED: placebo=39 (baseline denom. Placebo=39; total=81)	Any treatment-emergent adverse event48 weeks	Safety: TEAE, n/N per arm: Anti-ST2: other; Affected=34/42; placebo: other; Affected=28/39 (also “Adverse Event Rate in the 48 weeks” outcome: Anti-ST2=34; placebo=28)	NR/not obtainable: no comparative effect with precision was present; see per-arm summary.	ClinicalTrials.gov NCT03615040	No: transcribed as reported; S1 adds no calculation.	
	Active: STARTED: Anti-ST2=42 (baseline denom anti-ST2=42; total=81). Placebo: STARTED: placebo=39 (baseline denom. Placebo=39; total=81)	Serious adverse events. Outcome-specific timepoint NR; trial follow-up context: 48-week randomized treatment period plus 12-week follow-up = 60 weeks total observation; primary exacerbation endpoint assessed 0-48 weeks; safety assessed through week 60	Safety: SAE, n/N per arm: Anti-ST2: serious. Affected=12/42; placebo: serious. Affected=16/39 (also SAE Rate outcome: Anti-ST2=12; placebo=16)	NR/not obtainable: no comparative effect with precision was present; see per-arm summary.	ClinicalTrials.gov NCT03615040	No: transcribed as reported; S1 adds no calculation.	
	Active: STARTED: Anti-ST2=42 (baseline denom anti-ST2=42; total=81). Placebo: STARTED: placebo=39 (baseline denom. Placebo=39; total=81)	AEs leading to discontinuation. Outcome-specific timepoint NR; trial follow-up context: 48-week randomized treatment period plus 12-week follow-up = 60 weeks total observation; primary exacerbation endpoint assessed 0-48 weeks, safety assessed through week 60	Safety: discontinuation due to AE, n/N per arm: Not obtainable: the raw registry contains participant-flow, outcome, and adverse-events modules, but none report per-arm discontinuation specifically due to adverse events; the abstract also omits those counts (not obtainable)	NR/not obtainable: no comparative effect with precision was present; see per-arm summary.	ClinicalTrials.gov NCT03615040	Not applicable: NR/not obtainable.	
	Active: STARTED: Anti-ST2=42 (baseline denom anti-ST2=42; total=81). Placebo: STARTED: placebo=39 (baseline denom. Placebo=39; total=81)	All-cause death. Outcome-specific timepoint NR; trial follow-up context: 48-week randomized treatment period plus 12-week follow-up = 60 weeks total observation; primary exacerbation endpoint assessed 0-48 weeks; safety assessed through week 60	Safety: death, n/N per arm: Anti-ST2: deaths=0/42; placebo: deaths=2/39	NR/not obtainable: no comparative effect with precision was present; see per-arm summary.	ClinicalTrials.gov NCT03615040	No: transcribed as reported; S1 adds no calculation.	
	Active: STARTED: Anti-ST2=42 (baseline denom anti-ST2=42; total=81). Placebo: STARTED: placebo=39 (baseline denom. Placebo=39; total=81)	AEs of special interest. Outcome-specific timepoint NR; trial follow-up context: 48-week randomized treatment period plus 12-week follow-up = 60 weeks total observation; primary exacerbation endpoint assessed 0-48 weeks; safety assessed through week 60	NR/not obtainable: no trial-specific numeric result for this domain was present in the extraction workbook.	NR/not obtainable: no trial-specific numeric result for this domain was present in the extraction workbook.	Extraction workbook (no matching trial-specific outcome row).	Not applicable: NR/not obtainable.	
S1.13 COURSE (NCT04039113) [26]							
COURSE	Active: 169 randomized to tezepelumab; 165 received treatment (4 randomized-in-error, never dosed, all in the tezepelumab arm). Placebo arm: 168 randomized = 168 treated (symmetric). Placebo: 168 randomized and treated with placebo. Total CT.Gov registry enrollment=337; as-treated efficacy/safety analysis population=333 (165+168): this 337-vs-333 gap is a real, expected registry-vs-as-treated discrepancy, not an extraction error.	Moderate/severe exacerbation rate52 weeks	Eosinophil 150-<300 cells/µL: exacerbation rate, active arm (tezepelumab): 1.64 exacerbations/year; eosinophil 150-<300 cells/µL: exacerbation rate, placebo arm: 2.47 exacerbations/year; eosinophil 150-<300 cells/µL: exacerbation adjusted annualized rate and RR vs placebo (95% CI) (prespecified subgroup): Tezepelumab 1.64 vs placebo 2.47 exacerbations/year; rate ratio 0.66 (95% CI: 0.42-1.04); prespecified subgroup analysis. Eosinophil <150 cells/µL: exacerbation-adjusted annualized rate and RR vs placebo (95% CI) (prespecified subgroup): Tezepelumab 2.04 vs placebo 1.71 exacerbations/year; rate ratio 1.19 (95% CI: 0.75-1.90); favors placebo (no benefit/possible harm signal in the lowest-BEC prespecified subgroup). Eosinophil <150 cells/µL: exacerbation rate, active arm (tezepelumab): 2.04 exacerbations/year; eosinophil <150 cells/µL: exacerbation rate, placebo arm: 1.71 exacerbations/year; eosinophil ≥300 cells/µL: exacerbation adjusted annualized rate and RR vs placebo (95% CI) (prespecified subgroup): Tezepelumab 1.20 vs placebo 2.24 exacerbations/year; rate ratio 0.54 (95% CI: 0.25-1.15): largest apparent (non-significant) benefit signal, in the highest-BEC prespecified subgroup. Eosinophil ≥300 cells/µL: exacerbation rate, active arm (tezepelumab): 1.20 exacerbations/year; eosinophil ≥300 cells/µL: exacerbation rate, placebo arm: 2.24 exacerbations/year; Exacerbation - adjusted annualized rate, placebo arm: 2.11 exacerbations/year (moderate-or-severe, over 52 weeks, negative binomial model). Exacerbation: adjusted annualized rate, active arm(s): Tezepelumab 420 mg: 1.75 exacerbations/year (moderate-or-severe, over 52 weeks, negative binomial model) vs 2.11/year placebo.	Eosinophil ≥150 cells/µL (pooled post-hoc subgroup): exacerbation RR vs placebo (95% CI): Rate ratio 0.63 (95% CI: 0.43-0.93): statistically significant post-hoc (non-prespecified) pooled subgroup; explicitly described by the trialists/editorialists as hypothesis-generating, not confirmatory. Eosinophil ≥300 cells/µL AND FeNO ≥25 ppb (post-hoc combined biomarker subgroup): exacerbation RR vs placebo (95% CI): Rate ratio 0.37 (95% CI: 0.06-0.45): post-hoc analysis in an extremely small dual-biomarker-positive subgroup; hypothesis-generating only, not confirmatory. Exacerbation: rate ratio vs placebo, 95%/90% CI: RR 0.83; prespecified two-sided 90% CI 0.64-1.06 with one-sided P=0.1042. The same registry analysis posts a native two-sided 95% CI: 0.61-1.11 with two-sided P=0.2085. Primary endpoint not met.	PMID 39653044; PMC12433091; PMID 40266168PubMed abstract ()PMC12433091; PMC12340292; ClinicalTrials.gov NCT04039113	No: transcribed as reported; S1 adds no calculation.	
	Active: 169 randomized to tezepelumab; 165 received treatment (4 randomized-in-error, never dosed, all in the tezepelumab arm). Placebo arm: 168 randomized = 168 treated (symmetric). Placebo: 168 randomized and treated with placebo. Total CT.Gov registry enrollment=337; as-treated efficacy/safety analysis population=333 (165+168): this 337-vs-333 gap is a real, expected registry-vs-as-treated discrepancy, not an extraction error.	Pre-bronchodilator FEV1. Outcome-specific timepoint NR; trial follow-up context: 52-week treatment period plus 12-week post-treatment follow-up (total ~64 weeks)	FEV1 - LS-mean change from baseline, active arm: Tezepelumab 0.026 L (SE 0.015)FEV1 - LS-mean change from baseline, placebo arm: placebo -0.029 L (SE 0.015)	FEV1 - LS-mean difference vs placebo (mL), SE/95% CI: Arithmetic arm difference: 0.026 − (−0.029) = 0.055 L (55 mL) favoring tezepelumab. No native between-group SE or confidence interval is posted; none is reconstructed because covariance between the model-derived arm estimates is unknown.	ClinicalTrials.gov NCT04039113	Yes: arithmetic arm difference only; no inferential SE/CI reconstructed.	
	Active: 169 randomized to tezepelumab; 165 received treatment (4 randomized-in-error, never dosed, all in the tezepelumab arm). Placebo arm: 168 randomized = 168 treated (symmetric). Placebo: 168 randomized and treated with placebo. Total CT.Gov registry enrollment=337; as-treated efficacy/safety analysis population=333 (165+168): this 337-vs-333 gap is a real, expected registry-vs-as-treated discrepancy, not an extraction error.	Post-bronchodilator FEV1. Outcome-specific timepoint NR; trial follow-up context: 52-week treatment period plus 12-week post-treatment follow-up (total ~64 weeks)	NR/not obtainable: no trial-specific numeric result for this domain was present in the extraction workbook.	NR/not obtainable: no trial-specific numeric result for this domain was present in the extraction workbook.	Extraction workbook (no matching trial-specific outcome row).	Not applicable: NR/not obtainable.	
	Active: 169 randomized to tezepelumab; 165 received treatment (4 randomized-in-error, never dosed, all in the tezepelumab arm). Placebo arm: 168 randomized = 168 treated (symmetric). Placebo: 168 randomized and treated with placebo. Total CT.Gov registry enrollment=337; as-treated efficacy/safety analysis population=333 (165+168): this 337-vs-333 gap is a real, expected registry-vs-as-treated discrepancy, not an extraction error.	SGRQ total score Outcome-specific timepoint NR; trial follow-up context: 52-week treatment period plus 12-week post-treatment follow-up (total ~64 weeks)	SGRQ - LS-mean change from baseline, active arm: Tezepelumab -4.796 (SE 1.176)SGRQ - LS-mean change from baseline, placebo arm: placebo -1.863 (SE 1.189)	SGRQ - LS-mean difference vs placebo, SE/95% CI: Arithmetic arm difference: −4.796 − (−1.863) = −2.933 SGRQ points favoring tezepelumab. No native between-group SE or confidence interval is posted; none is reconstructed because covariance between the model-derived arm estimates is unknown.	ClinicalTrials.gov NCT04039113	Yes: arithmetic arm difference only; no inferential SE/CI reconstructed.	
	Active: 169 randomized to tezepelumab; 165 received treatment (4 randomized-in-error, never dosed, all in the tezepelumab arm). Placebo arm: 168 randomized = 168 treated (symmetric). Placebo: 168 randomized and treated with placebo. Total CT.Gov registry enrollment=337; as-treated efficacy/safety analysis population=333 (165+168): this 337-vs-333 gap is a real, expected registry-vs-as-treated discrepancy, not an extraction error.	E-RS:COPD. Outcome-specific timepoint NR; trial follow-up context: 52-week treatment period plus 12-week post-treatment follow-up (total ~64 weeks)	E-RS - LS-mean change from baseline, active arm: Not obtainable - see placebo-arm E-RS row for registration-status resolution (not obtainable)E-RS - LS-mean change from baseline, placebo arm: Not obtainable (numeric). Registration status resolved: E-RS is reportedly assessed as a secondary outcome per Afrose et al. commentary text (“Evaluating Respiratory Symptoms score”) but is NOT among CT.Gov’s 11 registered secondary_outcomes ( and fully enumerated : CAT total score, SGRQ total score + MCID responder proportion, pre-BD FEV1, three exacerbation-timing/severity outcomes, PK, ADA - no E-RS entry). CAT is the registered symptom PRO, not E-RS (not obtainable).	E-RS - LS-mean difference vs placebo, SE/95% CI: Not obtainable - see placebo-arm E-RS row for registration-status resolution (not obtainable)	Same as placebo-arm E-RS rowPMC12340292	Not applicable: NR/not obtainable.	
	Active: 169 randomized to tezepelumab; 165 received treatment (4 randomized-in-error, never dosed, all in the tezepelumab arm). Placebo arm: 168 randomized = 168 treated (symmetric). Placebo: 168 randomized and treated with placebo. Total CT.Gov registry enrollment=337; as-treated efficacy/safety analysis population=333 (165+168): this 337-vs-333 gap is a real, expected registry-vs-as-treated discrepancy, not an extraction error.	Time to first exacerbation. Outcome-specific timepoint NR; trial follow-up context: 52-week treatment period plus 12-week post-treatment follow-up (total ~64 weeks)	NR/not obtainable: no trial-specific numeric result for this domain was present in the extraction workbook.	NR/not obtainable: no trial-specific numeric result for this domain was present in the extraction workbook.	Extraction workbook (no matching trial-specific outcome row).	Not applicable: NR/not obtainable.	
	Active: 169 randomized to tezepelumab; 165 received treatment (4 randomized-in-error, never dosed, all in the tezepelumab arm). Placebo arm: 168 randomized = 168 treated (symmetric). Placebo: 168 randomized and treated with placebo. Total CT.Gov registry enrollment=337; as-treated efficacy/safety analysis population=333 (165+168): this 337-vs-333 gap is a real, expected registry-vs-as-treated discrepancy, not an extraction error.	Rescue-medication use. Outcome-specific timepoint NR; trial follow-up context: 52-week treatment period plus 12-week post-treatment follow-up (total ~64 weeks)	NR/not obtainable: no trial-specific numeric result for this domain was present in the extraction workbook.	NR/not obtainable: no trial-specific numeric result for this domain was present in the extraction workbook.	Extraction workbook (no matching trial-specific outcome row).	Not applicable: NR/not obtainable.	
	Active: 169 randomized to tezepelumab; 165 received treatment (4 randomized-in-error, never dosed, all in the tezepelumab arm). Placebo arm: 168 randomized = 168 treated (symmetric). Placebo: 168 randomized and treated with placebo. Total CT.Gov registry enrollment=337; as-treated efficacy/safety analysis population=333 (165+168): this 337-vs-333 gap is a real, expected registry-vs-as-treated discrepancy, not an extraction error.	Any treatment-emergent adverse event. Outcome-specific timepoint NR; trial follow-up context: 52-week treatment period plus 12-week post-treatment follow-up (total ~64 weeks)	Safety - TEAE, n/N per arm: Tezepelumab 133/165 (81%); placebo 126/168 (75%)	NR/not obtainable: no comparative effect with precision was present; see per-arm summary.	PubMed abstract ()	No: transcribed as reported; S1 adds no calculation.	
	Active: 169 randomized to tezepelumab; 165 received treatment (4 randomized-in-error, never dosed, all in the tezepelumab arm). Placebo arm: 168 randomized = 168 treated (symmetric). Placebo: 168 randomized and treated with placebo. Total CT.Gov registry enrollment=337; as-treated efficacy/safety analysis population=333 (165+168): this 337-vs-333 gap is a real, expected registry-vs-as-treated discrepancy, not an extraction error.	Serious adverse events; week 64, 52-week, 12-week	Safety - SAE, n/N per arm: Abstract-derived (on-treatment framing): Tezepelumab 49/165 (30%); placebo 50/168 (30%). CT.Gov Results-module adverseEventsModule (timeFrame “From first dose of study drug until end of study at week 64,” i.e., full 52-week treatment + 12-week follow-up): Tezepelumab 56/165; placebo 58/168. Both figures are accurate transcriptions of their respective sources; the higher registry figure reflects the longer ascertainment window, not an error in either source.	NR/not obtainable: no comparative effect with precision was present; see per-arm summary.	ClinicalTrials.gov NCT04039113; PMID 39653044	No: transcribed as reported; S1 adds no calculation.	
	Active: 169 randomized to tezepelumab; 165 received treatment (4 randomized-in-error, never dosed, all in the tezepelumab arm). Placebo arm: 168 randomized = 168 treated (symmetric). Placebo: 168 randomized and treated with placebo. Total CT.Gov registry enrollment=337; as-treated efficacy/safety analysis population=333 (165+168): this 337-vs-333 gap is a real, expected registry-vs-as-treated discrepancy, not an extraction error.	AEs leading to discontinuation. Outcome-specific timepoint NR; trial follow-up context: 52-week treatment period plus 12-week post-treatment follow-up (total ~64 weeks)	Safety - discontinuation due to AE, n/N per arm: Tezepelumab 2/165; placebo 2/168 (discontinuation due to adverse event)	NR/not obtainable: no comparative effect with precision was present; see per-arm summary.	ClinicalTrials.gov NCT04039113	No: transcribed as reported; S1 adds no calculation.	
	Active: 169 randomized to tezepelumab; 165 received treatment (4 randomized-in-error, never dosed, all in the tezepelumab arm). Placebo arm: 168 randomized = 168 treated (symmetric). Placebo: 168 randomized and treated with placebo. Total CT.Gov registry enrollment=337; as-treated efficacy/safety analysis population=333 (165+168): this 337-vs-333 gap is a real, expected registry-vs-as-treated discrepancy, not an extraction error.	All-cause death; week 64	Safety - death, n/N per arm: Abstract-derived (on-treatment framing): Tezepelumab 2/165; placebo 3/168 (5 total deaths on study treatment; none investigator-assessed as treatment-related). CT.Gov Results-module adverseEventsModule (timeFrame “From first dose of study drug until end of study at week 64”): Tezepelumab deathsNumAffected=3/165; placebo deathsNumAffected=6/168 (9 total). Both figures are accurate transcriptions of their respective sources; the near-doubled placebo-arm registry figure reflects the longer ascertainment window, not an error in either source.	NR/not obtainable: no comparative effect with precision was present; see per-arm summary.	ClinicalTrials.gov NCT04039113; PMID 39653044	No: transcribed as reported; S1 adds no calculation.	
	Active: 169 randomized to tezepelumab; 165 received treatment (4 randomized-in-error, never dosed, all in the tezepelumab arm). Placebo arm: 168 randomized = 168 treated (symmetric). Placebo: 168 randomized and treated with placebo. Total CT.Gov registry enrollment=337; as-treated efficacy/safety analysis population=333 (165+168): this 337-vs-333 gap is a real, expected registry-vs-as-treated discrepancy, not an extraction error.	AEs of special interest. Outcome-specific timepoint NR; trial follow-up context: 52-week treatment period plus 12-week post-treatment follow-up (total ~64 weeks)	NR/not obtainable: no trial-specific numeric result for this domain was present in the extraction workbook.	NR/not obtainable: no trial-specific numeric result for this domain was present in the extraction workbook.	Extraction workbook (no matching trial-specific outcome row).	Not applicable: NR/not obtainable.	
S1.14 Benralizumab ph2a (NCT01227278) [39]							
Benralizumab ph2a	Active: benralizumab 100 mg SC: 51 (single active-dose arm; no other doses tested). Placebo: 50 (of 101 total randomized: 51 benralizumab, 50 placebo), ITT population. CT.Gov enrollment field independently confirmed at 421 (a trial-level total, almost certainly reflecting patients screened rather than randomized, given the paper’s own description of substantial screen failure under the sputum-eosinophilia eligibility gate): this is a real but explainable registry/publication mismatch, not an extraction error.	Moderate/severe exacerbation rate; week 56	Exacerbation: adjusted annualized rate, active arm(s): benralizumab 100 mg: 0.95 (95% CI: 0.68-1.29), per-protocol population, n=40, at week 56Exacerbation: adjusted annualized rate, placebo arm: 0.92 (95% CI: 0.67-1.25), per-protocol population, n=42, at week 56	Eosinophil <150 cells/µL: exacerbation rate, benralizumab vs placebo: benralizumab 1.02 vs placebo 0.80 (benralizumab numerically higher rate below this cutoff), p=0.70 (non-significant); no formal rate-ratio value/CI given, only per-arm rates and p-value; eosinophil <200 cells/µL: exacerbation rate, benralizumab vs placebo: benralizumab 1.15 vs placebo 0.70 (benralizumab numerically higher), p=0.27 (non-significant).Eosinophil <300 cells/µL: exacerbation rate, benralizumab vs placebo: benralizumab 1.25 vs placebo 0.87 (benralizumab numerically higher), p=0.28 (non-significant).Eosinophil ≥150 cells/µL subgroup: exacerbation-rate result vs placebo (significance): Per-protocol subgroup n=54 (66%): 26 placebo, 28 benralizumab. Reduction in exacerbation rate with benralizumab vs placebo non-significant, p=0.84. No explicit rate-ratio point estimate or CI given in accessible text. Eosinophil ≥200 cells/µL subgroup: exacerbation-rate result vs placebo (significance): Per-protocol subgroup ≥200 cells/µL: n=39/82 (48%), placebo 20 and benralizumab 19; exacerbation-rate reduction was not significant, P=0.26.Eosinophil ≥300 cells/µL subgroup: exacerbation-rate result vs placebo (significance): Per-protocol subgroup ≥300 cells/µL: n=21/82 (26%), placebo 7 and benralizumab 14; exacerbation-rate reduction was not significant, p=0.28. Exacerbation: rate ratio/rate reduction vs placebo, 95% CI: Reported as rate reduction (not rate ratio): benralizumab vs placebo = −3% (95% CI: −58 to 33), p=0.94, by van Elteren test (primary analysis, per-protocol population); qualitatively corroborated by Poisson regression and negative binomial models, but exact numeric estimates from those secondary models were not present in the extracted text.	PMC5082845	No: transcribed as reported; S1 adds no calculation.	
	Active: benralizumab 100 mg SC: 51 (single active-dose arm; no other doses tested). Placebo: 50 (of 101 total randomized: 51 benralizumab, 50 placebo), ITT population. CT.Gov enrollment field independently confirmed at 421 (a trial-level total, almost certainly reflecting patients screened rather than randomized, given the paper’s own description of substantial screen failure under the sputum-eosinophilia eligibility gate): this is a real but explainable registry/publication mismatch, not an extraction error.	Pre-bronchodilator FEV1: week 56, week 4, week 80, 32 weeks	FEV1 (pre-bronchodilator): mean/LS-mean change from baseline, active arm(s): benralizumab: pre-bronchodilator FEV1 mean change +0.13 L (SD 0.41), baseline to week 56, p=0.014 vs placebo. Post-bronchodilator FEV1: mean change +0.09 L (SD 0.39), p=0.014 vs placebo. Improvements evident by week 4 and sustained to week 80 (32 weeks after last dose); per-protocol population. FEV1 (pre-bronchodilator): mean/LS-mean change from baseline, placebo arm: placebo: pre-bronchodilator FEV1 mean change −0.06 L (SD 0.24), baseline to week 56 (reported as “mean change,” not explicitly labelled LS-mean, though between-group comparisons used ANCOVA per Methods). Post-bronchodilator FEV1: mean change −0.08 L (SD 0.21). Per-protocol population.	Eosinophil ≥150 cells/µL subgroup: FEV1 (pre-bronchodilator) improvement vs placebo (significance): Significant improvement in pre-bronchodilator FEV1 at week 56 with benralizumab vs placebo, p=0.031; no explicit mL value or CI given in extracted text; eosinophil ≥200 cells/µL subgroup: FEV1 (pre-bronchodilator) improvement vs placebo (significance): Statistically significant improvement in pre-bronchodilator FEV1 at week 56 with benralizumab vs placebo, p=0.035. No exact mL value or CI given in accessible text. Eosinophil ≥300 cells/µL subgroup: FEV1 (pre-bronchodilator) improvement vs placebo (significance): Non-significant improvement in pre-bronchodilator FEV1 at week 56 with benralizumab vs placebo, p=0.22. No exact mL value or CI given in accessible text. FEV1 (pre-bronchodilator): difference vs placebo (mL), SE/95% CI: Not obtainable from any available source: the accessible full text gives only per-arm mean changes (with SD) and p=0.014; no explicit between-group LS-mean difference in mL with SE or 95% CI is reported anywhere in the extracted text (not obtainable).	PMC5082845	No: transcribed as reported; S1 adds no calculation.	
	Active: benralizumab 100 mg SC: 51 (single active-dose arm; no other doses tested). Placebo: 50 (of 101 total randomized: 51 benralizumab, 50 placebo), ITT population. CT.Gov enrollment field independently confirmed at 421 (a trial-level total, almost certainly reflecting patients screened rather than randomized, given the paper's own description of substantial screen failure under the sputum-eosinophilia eligibility gate): this is a real but explainable registry/publication mismatch, not an extraction error.	Post-bronchodilator FEV1: week 56	FEV1 (post-bronchodilator): mean/LS-mean change from baseline, active arm(s): benralizumab: post-bronchodilator FEV1 mean change +0.09 L (SD 0.39), baseline to week 56, p=0.014 vs placebo. FEV1 (post-bronchodilator): mean/LS-mean change from baseline, placebo arm: placebo: post-bronchodilator FEV1 mean change −0.08 L (SD 0.21), baseline to week 56, per-protocol population.	FEV1 (post-bronchodilator): difference vs placebo (mL), SE/95% CI: Not obtainable: per-arm post-BD changes are reported, but no explicit between-group difference in mL with SE or 95% CI is supplied (not obtainable).	not obtainable: confirmed: no mL/SE/CI between-group difference reported in extracted text for the post-BD comparison, same as pre-BDPMC5082845	No: transcribed as reported; S1 adds no calculation.	
	Active: benralizumab 100 mg SC: 51 (single active-dose arm; no other doses tested). Placebo: 50 (of 101 total randomized: 51 benralizumab, 50 placebo), ITT population. CT.Gov enrollment field independently confirmed at 421 (a trial-level total, almost certainly reflecting patients screened rather than randomized, given the paper's own description of substantial screen failure under the sputum-eosinophilia eligibility gate): this is a real but explainable registry/publication mismatch, not an extraction error.	SGRQ total score; week 56	SGRQ-C total score: LS-mean/mean change from baseline, active arm(s): benralizumab: mean change −5.51 in SGRQ-C total score, baseline to week 56 (reported as “mean change,” not explicitly labelled LS-mean). Placebo: −4.43. Difference between groups was not statistically significant (no exact p-value or difference figure with CI given in the accessible text).SGRQ-C total score: LS-mean/mean change from baseline, placebo arm: −4.43 (SGRQ-C total score), change from baseline to week 56	Eosinophil subgroup (≥150/≥200/≥300 cells/µL): SGRQ-C and CRQ-SAS numeric estimates vs placebo (qualitative): SGRQ-C change from baseline, mean (SD; n), placebo vs benralizumab: overall −4.43 (11.71;42) vs −5.51 (16.64;37), P=0.739; <200 cells/µL −4.26 (10.17;21) vs −2.10 (16.47;20), P=0.614; ≥200 −4.79 (13.64;20) vs −9.52 (16.42;17), P=0.345; <300 −4.40 (11.04;34) vs −1.90 (14.97;25), P=0.463; ≥300 −5.11 (16.24;7) vs −13.03 (18.08;12), P=0.354. CRQ-SAS numeric subgroup estimates remain unavailable. SGRQ-C: LS-mean/mean difference vs placebo, SE/95% CI: Not obtainable: per-arm SGRQ-C changes and narrative nonsignificance are reported, but no explicit between-group difference with SE or 95% CI is supplied (not obtainable).	PMC5082845not obtainable: confirmed: text states only that “no significant differences between treatment groups were noted” for SGRQ-C/CRQ-SAS/BODE; no exp…	No: transcribed as reported; S1 adds no calculation.	
	Active: benralizumab 100 mg SC: 51 (single active-dose arm; no other doses tested). Placebo: 50 (of 101 total randomized: 51 benralizumab, 50 placebo), ITT population. CT.Gov enrollment field independently confirmed at 421 (a trial-level total, almost certainly reflecting patients screened rather than randomized, given the paper’s own description of substantial screen failure under the sputum-eosinophilia eligibility gate): this is a real but explainable registry/publication mismatch, not an extraction error.	E-RS:COPD. Outcome-specific timepoint NR; trial follow-up context: Dosing period 48 weeks (8 doses); efficacy assessments (including the primary endpoint) through week 56; safety monitoring continued a further 24 weeks (to week 80), i.e., 32 weeks after the last dose (week 48).	E-RS: LS-mean change from baseline, active arm(s): Not obtainable: E-RS was not used; this trial used EXACT-PRO (not obtainable).E-RS: LS-mean change from baseline, placebo arm: Not obtainable: E-RS was not used; this trial used EXACT-PRO (not obtainable).	E-RS: LS-mean difference vs placebo, SE/95% CI: Not obtainable: E-RS was not used; this trial used EXACT-PRO (not obtainable).	not obtainable: same reason (E-RS not used; trial used EXACT-PRO)not obtainable: confirmed this trial did not use E-RS; it used EXACT-PRO as an exploratory endpoint instead, and exploratory numeric results were…not obtainable: E-RS not used in this trial	Not applicable: NR/not obtainable.	
	Active: benralizumab 100 mg SC: 51 (single active-dose arm; no other doses tested). Placebo: 50 (of 101 total randomized: 51 benralizumab, 50 placebo), ITT population. CT.Gov enrollment field independently confirmed at 421 (a trial-level total, almost certainly reflecting patients screened rather than randomized, given the paper’s own description of substantial screen failure under the sputum-eosinophilia eligibility gate): this is a real but explainable registry/publication mismatch, not an extraction error.	Time to first exacerbation. Outcome-specific timepoint NR; trial follow-up context: Dosing period 48 weeks (8 doses); efficacy assessments (including the primary endpoint) through week 56; safety monitoring continued a further 24 weeks (to week 80), i.e., 32 weeks after the last dose (week 48).	NR/not obtainable: no trial-specific numeric result for this domain was present in the extraction workbook.	NR/not obtainable: no trial-specific numeric result for this domain was present in the extraction workbook.	Extraction workbook (no matching trial-specific outcome row).	Not applicable: NR/not obtainable.	
	Active: benralizumab 100 mg SC: 51 (single active-dose arm; no other doses tested). Placebo: 50 (of 101 total randomized: 51 benralizumab, 50 placebo), ITT population. CT.Gov enrollment field independently confirmed at 421 (a trial-level total, almost certainly reflecting patients screened rather than randomized, given the paper’s own description of substantial screen failure under the sputum-eosinophilia eligibility gate): this is a real but explainable registry/publication mismatch, not an extraction error.	Rescue-medication use. Outcome-specific timepoint NR; trial follow-up context: Dosing period 48 weeks (8 doses); efficacy assessments (including the primary endpoint) through week 56; safety monitoring continued a further 24 weeks (to week 80), i.e., 32 weeks after the last dose (week 48).	NR/not obtainable: no trial-specific numeric result for this domain was present in the extraction workbook.	NR/not obtainable: no trial-specific numeric result for this domain was present in the extraction workbook.	Extraction workbook (no matching trial-specific outcome row).	Not applicable: NR/not obtainable.	
	Active: benralizumab 100 mg SC: 51 (single active-dose arm; no other doses tested). Placebo: 50 (of 101 total randomized: 51 benralizumab, 50 placebo), ITT population. CT.Gov enrollment field independently confirmed at 421 (a trial-level total, almost certainly reflecting patients screened rather than randomized, given the paper’s own description of substantial screen failure under the sputum-eosinophilia eligibility gate): this is a real but explainable registry/publication mismatch, not an extraction error.	Any treatment-emergent adverse event. Outcome-specific timepoint NR; trial follow-up context: Dosing period 48 weeks (8 doses); efficacy assessments (including the primary endpoint) through week 56; safety monitoring continued a further 24 weeks (to week 80), i.e., 32 weeks after the last dose (week 48).	Safety: TEAE, overall n/N per arm (any TEAE): TEAEs : placebo=41; benralizumab 100 mg=45 (n/N = 41/50 placebo, 45/51 benralizumab, N from participant flow STARTED)	NR/not obtainable: no comparative effect with precision was present; see per-arm summary.	PMC5082845	No: transcribed as reported; S1 adds no calculation.	
	Active: benralizumab 100 mg SC: 51 (single active-dose arm; no other doses tested). Placebo: 50 (of 101 total randomized: 51 benralizumab, 50 placebo), ITT population. CT.Gov enrollment field independently confirmed at 421 (a trial-level total, almost certainly reflecting patients screened rather than randomized, given the paper’s own description of substantial screen failure under the sputum-eosinophilia eligibility gate): this is a real but explainable registry/publication mismatch, not an extraction error.	Serious adverse events. Outcome-specific timepoint NR; trial follow-up context: dosing period 48 weeks (8 doses); efficacy assessments (including the primary endpoint) through week 56; safety monitoring continued a further 24 weeks (to week 80), i.e., 32 weeks after the last dose (week 48).	Safety: SAE, n/N per arm: Serious TEAEs: benralizumab 14/51 (27%) vs placebo 9/50 (18%); numerically higher with benralizumab; none investigator-assessed as treatment-related.	NR/not obtainable: no comparative effect with precision was present; see per-arm summary.	PMC5082845; PMID 25208464	No: transcribed as reported; S1 adds no calculation.	
	Active: benralizumab 100 mg SC: 51 (single active-dose arm; no other doses tested). Placebo: 50 (of 101 total randomized: 51 benralizumab, 50 placebo), ITT population. CT.Gov enrollment field independently confirmed at 421 (a trial-level total, almost certainly reflecting patients screened rather than randomized, given the paper’s own description of substantial screen failure under the sputum-eosinophilia eligibility gate): this is a real but explainable registry/publication mismatch, not an extraction error.	AEs leading to discontinuation. Outcome-specific timepoint NR; trial follow-up context: Dosing period 48 weeks (8 doses); efficacy assessments (including the primary endpoint) through week 56; safety monitoring continued a further 24 weeks (to week 80), i.e., 32 weeks after the last dose (week 48).	Safety: discontinuation due to AE, n/N per arm: Treatment-emergent adverse events leading to discontinuation: placebo 2/50; benralizumab 3/51.	NR/not obtainable: no comparative effect with precision was present; see per-arm summary.	PMC5082845	No: transcribed as reported; S1 adds no calculation.	
	Active: benralizumab 100 mg SC: 51 (single active-dose arm; no other doses tested). Placebo: 50 (of 101 total randomized: 51 benralizumab, 50 placebo), ITT population. CT.Gov enrollment field independently confirmed at 421 (a trial-level total, almost certainly reflecting patients screened rather than randomized, given the paper’s own description of substantial screen failure under the sputum-eosinophilia eligibility gate): this is a real but explainable registry/publication mismatch, not an extraction error.	All-cause death. Outcome-specific timepoint NR; trial follow-up context: Dosing period 48 weeks (8 doses); efficacy assessments (including the primary endpoint) through week 56; safety monitoring continued a further 24 weeks (to week 80), i.e., 32 weeks after the last dose (week 48).	Safety: death, n/N per arm: benralizumab: 2/51 (4%) deaths: 1 myocardial infarction, 1 sudden death of unknown cause, neither treatment-related. Placebo: 1/50 (2%) death: bronchitis, occurring after study completion, not treatment-related.	NR/not obtainable: no comparative effect with precision was present; see per-arm summary.	PMC5082845	No: transcribed as reported; S1 adds no calculation.	
	Active: benralizumab 100 mg SC: 51 (single active-dose arm; no other doses tested). Placebo: 50 (of 101 total randomized: 51 benralizumab, 50 placebo), ITT population. CT.Gov enrollment field independently confirmed at 421 (a trial-level total, almost certainly reflecting patients screened rather than randomized, given the paper’s own description of substantial screen failure under the sputum-eosinophilia eligibility gate): this is a real but explainable registry/publication mismatch, not an extraction error.	AEs of special interest. Outcome-specific timepoint NR; trial follow-up context: Dosing period 48 weeks (8 doses); efficacy assessments (including the primary endpoint) through week 56; safety monitoring continued a further 24 weeks (to week 80), i.e., 32 weeks after the last dose (week 48).	NR/not obtainable: no trial-specific numeric result for this domain was present in the extraction workbook.	NR/not obtainable: no trial-specific numeric result for this domain was present in the extraction workbook.	Extraction workbook (no matching trial-specific outcome row).	Not applicable: NR/not obtainable.	
	Active: 119 (mepolizumab arm, STARTED milestone). Placebo: 119 (Placebo arm, STARTED milestone)	Moderate/severe exacerbation rate48 weeks, 48-week	Exacerbation: adjusted mean count over 48 weeks (as-reported, not annualized), active arm(s) (mepolizumab): 2.80 (95% CI: 2.36-3.23): adjusted mean number of moderate-or-severe exacerbations over 48 weeks, mepolizumab arm (explicitly not an annualized per-patient-year rate). Exacerbation: adjusted mean count over 48 weeks (as-reported, not annualized), placebo arm: 3.45 (95% CI: 2.94-3.95): adjusted mean number of moderate-or-severe exacerbations over 48 weeks, placebo arm (explicitly not an annualized per-patient-year rate). Exacerbation: adjusted mean number of moderate-or-severe exacerbations over 48 weeks, mepolizumab arm: adjusted mean number of moderate-or-severe exacerbations over 48 weeks, mepolizumab arm: 2.80 (95% CI: 2.36-3.23). This is the as-reported 48-week adjusted mean count, NOT a per-patient-year annualized rate: no annualized rate is reported in the abstract or the CT.Gov record. Exacerbation: adjusted mean number of moderate-or-severe exacerbations over 48 weeks, placebo arm: adjusted mean number of moderate-or-severe exacerbations over 48 weeks, placebo arm: 3.45 (95% CI: 2.94-3.95). This is the as-reported 48-week adjusted mean count, NOT a per-patient-year annualized rate.	Exacerbation: rate/risk ratio vs placebo, 95% CI: Risk ratio 0.81 (95% CI: 0.66-1.00) for moderate-or-severe exacerbations, mepolizumab vs placebo, over 48 weeks; upper CI bound reaches the null	PMID 40305842Abstract: ‘risk ratio 0.81; 95% CI, 0.66 to 1.00’	No: transcribed as reported; S1 adds no calculation.	
	Active: 119 (mepolizumab arm, STARTED milestone). Placebo: 119 (Placebo arm, STARTED milestone)	Pre-bronchodilator FEV1. Outcome-specific timepoint NR; trial follow-up context: 48-week treatment/follow-up period, monthly (every-4-week) dosing; absolute maximum 49 weeks allowing the ±1 week visit window at week 48. Secondary-outcome assessments at weeks 0, 4, 8, 12, 24, 36, 48.	FEV1: LS-mean change from baseline, active arm(s): NR/not obtainable: workbook value cell is empty (not obtainable) FEV1: LS-mean change from baseline, placebo arm: NR/not obtainable: workbook value cell is empty (not obtainable)	FEV1: LS-mean difference vs placebo (mL), SE/95% CI: NR/not obtainable: workbook value cell is empty (not obtainable)	Same re-check as aboveClinicalTrials.gov NCT04075331	Not applicable: NR/not obtainable.	
	Active: 119 (mepolizumab arm, STARTED milestone). Placebo: 119 (Placebo arm, STARTED milestone)	Post-bronchodilator FEV1. Outcome-specific timepoint NR; trial follow-up context: 48-week treatment/follow-up period, monthly (every-4-week) dosing; absolute maximum 49 weeks allowing the ±1 week visit window at week 48. Secondary-outcome assessments at weeks 0, 4, 8, 12, 24, 36, 48.	NR/not obtainable: no trial-specific numeric result for this domain was present in the extraction workbook.	NR/not obtainable: no trial-specific numeric result for this domain was present in the extraction workbook.	Extraction workbook (no matching trial-specific outcome row).	Not applicable: NR/not obtainable.	
	Active: 119 (mepolizumab arm, STARTED milestone). Placebo: 119 (placebo arm, STARTED milestone)	SGRQ total score; week 4, week 8, week 12, week 24, week 36, week 48	SGRQ: raw arm mean score by visit, active arm: mepolizumab raw mean (SD), n: baseline 69.8 (15.7), 111; week 4 65.5 (16.0), 102; week 8 64.3 (15.9), 86; week 12 64.3 (16.4), 79; week 24 64.3 (18.0), 73; week 36 61.2 (18.1), 60; week 48 60.7 (19.1), 67. These are raw scores, not LS-mean changes. SGRQ: raw arm mean score by visit, placebo arm: placebo raw mean (SD), n: baseline 72.0 (13.8), 117; week 4 66.2 (16.4), 95; week 8 64.6 (18.1), 84; week 12 63.3 (16.3), 85; week 24 61.7 (18.2), 76; week 36 60.4 (20.0), 67; week 48 62.4 (18.9), 77. These are raw scores, not LS-mean changes.	SGRQ: LS-mean difference vs placebo, SE/95% CI: week-48 SGRQ Mean Difference (Net), mepolizumab vs placebo: 0.57 (95% CI: -2.63 to 3.78), p=0.726 - from a mixed-effects model adjusting for baseline MRC dyspnea, prior hospitalization, visit, and baseline SGRQ.	ClinicalTrials.gov NCT04075331	No: transcribed as reported; S1 adds no calculation.	
	Active: 119 (mepolizumab arm, STARTED milestone). Placebo: 119 (Placebo arm, STARTED milestone)	E-RS:COPD. Outcome-specific timepoint NR; trial follow-up context: 48-week treatment/follow-up period, monthly (every-4-week) dosing; absolute maximum 49 weeks allowing the ±1 week visit window at week 48. Secondary-outcome assessments at weeks 0, 4, 8, 12, 24, 36, 48.	E-RS: LS-mean change from baseline, active arm(s): NR/not obtainable: workbook value cell is empty (not obtainable). E-RS: LS-mean change from baseline, placebo arm: NR/not obtainable: workbook value cell is empty (not obtainable).	E-RS: LS-mean difference vs placebo, SE/95% CI: NR/not obtainable: workbook value cell is empty (not obtainable).	Same re-check as aboveClinicalTrials.gov NCT04075331	Not applicable: NR/not obtainable.	
	Active: 119 (mepolizumab arm, STARTED milestone). Placebo: 119 (placebo arm, STARTED milestone)	Time to first exacerbation. Outcome-specific timepoint NR; trial follow-up context: 48-week treatment/follow-up period, monthly (every 4-week) dosing; absolute maximum 49 weeks allowing the ±1 week visit window at week 48. Secondary-outcome assessments at weeks 0, 4, 8, 12, 24, 36, 48.	NR/not obtainable: no trial-specific numeric result for this domain was present in the extraction workbook.	NR/not obtainable: no trial-specific numeric result for this domain was present in the extraction workbook.	Extraction workbook (no matching trial-specific outcome row).	Not applicable: NR/not obtainable.	
	Active: 119 (mepolizumab arm, STARTED milestone). Placebo: 119 (Placebo arm, STARTED milestone)	Rescue-medication use. Outcome-specific timepoint NR; trial follow-up context: 48-week treatment/follow-up period, monthly (every-4-week) dosing; absolute maximum 49 weeks allowing the ±1 week visit window at week 48. Secondary-outcome assessments at weeks 0, 4, 8, 12, 24, 36, 48.	NR/not obtainable: no trial-specific numeric result for this domain was present in the extraction workbook.	NR/not obtainable: no trial-specific numeric result for this domain was present in the extraction workbook.	Extraction workbook (no matching trial-specific outcome row).	Not applicable: NR/not obtainable.	
	Active: 119 (mepolizumab arm, STARTED milestone). Placebo: 119 (Placebo arm, STARTED milestone)	Any treatment-emergent adverse event. Outcome-specific timepoint NR; trial follow-up context: 48-week treatment/follow-up period, monthly (every-4-week) dosing; absolute maximum 49 weeks allowing the ±1 week visit window at week 48. Secondary-outcome assessments at weeks 0, 4, 8, 12, 24, 36, 48.	Safety: TEAE, n/N per arm: placebo 91/119 with >=1 AE; mepolizumab 91/118 with >=1 AE; incidence rate ratio 1.24 (95% CI: 0.98-1.56), p=0.070.	NR/not obtainable: no comparative effect with precision was present; see per-arm summary.	ClinicalTrials.gov NCT04075331	No: transcribed as reported; S1 adds no calculation.	
	Active: 119 (mepolizumab arm, STARTED milestone). Placebo: 119 (Placebo arm, STARTED milestone)	Serious adverse events. Outcome-specific timepoint NR; trial follow-up context: 48-week treatment/follow-up period, monthly (every-4-week) dosing; absolute maximum 49 weeks allowing the ±1 week visit window at week 48. Secondary-outcome assessments at weeks 0, 4, 8, 12, 24, 36, 48.	Safety: SAE, n/N per arm: placebo 3/119 with >=1 SAE (116/119 without); mepolizumab 2/118 with >=1 SAE (116/118 without); incidence rate ratio 0.69 (95% CI: 0.12-4.14), p=0.686. Safety population denominators 119/118 (differs from the randomized 119/119 by one mepolizumab-arm participant).	NR/not obtainable: no comparative effect with precision was present; see per-arm summary.	ClinicalTrials.gov NCT04075331	No: transcribed as reported; S1 adds no calculation.	
	Active: 119 (mepolizumab arm, STARTED milestone). Placebo: 119 (Placebo arm, STARTED milestone)	AEs leading to discontinuation. Outcome-specific timepoint NR; trial follow-up context: 48-week treatment/follow-up period, monthly (every-4-week) dosing; absolute maximum 49 weeks allowing the ±1 week visit window at week 48. Secondary-outcome assessments at weeks 0, 4, 8, 12, 24, 36, 48.	Safety: discontinuation due to AE, n/N per arm: NR/not obtainable: workbook value cell is empty (not obtainable).	NR/not obtainable: no comparative effect with precision was present; see per-arm summary.	ClinicalTrials.gov NCT04075331	Not applicable: NR/not obtainable.	
	Active: 119 (mepolizumab arm, STARTED milestone). Placebo: 119 (Placebo arm, STARTED milestone)	All-cause death. Outcome-specific timepoint NR; trial follow-up context: 48-week treatment/follow-up period, monthly (every-4-week) dosing; absolute maximum 49 weeks allowing the ±1 week visit window at week 48. Secondary-outcome assessments at weeks 0, 4, 8, 12, 24, 36, 48.	Safety: death, n/N per arm: All-cause death: placebo 11/119, mepolizumab 9/119 (HR 0.84, 95% CI: 0.35-2.04, p=0.705). Respiratory-cause death: placebo 8/119, mepolizumab 5/119 (HR 0.64, 95% CI: 0.21-1.95, p=0.432).	NR/not obtainable: no comparative effect with precision was present; see per-arm summary.	ClinicalTrials.gov NCT04075331	No: transcribed as reported; S1 adds no calculation.	
	Active: 119 (mepolizumab arm, STARTED milestone). Placebo: 119 (Placebo arm, STARTED milestone)	AEs of special interest. Outcome-specific timepoint NR; trial follow-up context: 48-week treatment/follow-up period, monthly (every-4-week) dosing; absolute maximum 49 weeks allowing the ±1 week visit window at week 48. Secondary-outcome assessments at weeks 0, 4, 8, 12, 24, 36, 48.	NR/not obtainable: no trial-specific numeric result for this domain was present in the extraction workbook.	NR/not obtainable: no trial-specific numeric result for this domain was present in the extraction workbook.	Extraction workbook (no matching trial-specific outcome row).	Not applicable: NR/not obtainable.	
	Active: benralizumab 100 mg SC: 51 (single active-dose arm; no other doses tested). Placebo: 50 (of 101 total randomized: 51 benralizumab, 50 placebo), ITT population. CT.Gov enrollment field independently confirmed at 421 (a trial-level total, almost certainly reflecting patients screened rather than randomized, given the paper’s own description of substantial screen failure under the sputum-eosinophilia eligibility gate): this is a real but explainable registry/publication mismatch, not an extraction error.	Moderate/severe exacerbation rate; week 56	Exacerbation: adjusted annualized rate, active arm(s): benralizumab 100 mg: 0.95 (95% CI: 0.68-1.29), per-protocol population, n=40, at week 56. Exacerbation: adjusted annualized rate, placebo arm: 0.92 (95% CI: 0.67-1.25), per-protocol population, n=42, at week 56	Eosinophil <150 cells/µL: exacerbation rate, benralizumab vs placebo: benralizumab 1.02 vs placebo 0.80 (benralizumab numerically higher rate below this cutoff), p=0.70 (non-significant); no formal rate-ratio value/CI given, only per-arm rates and p-value; eosinophil <200 cells/µL: exacerbation rate, benralizumab vs placebo: benralizumab 1.15 vs placebo 0.70 (benralizumab numerically higher), p=0.27 (non-significant).Eosinophil <300 cells/µL: exacerbation rate, benralizumab vs placebo: benralizumab 1.25 vs placebo 0.87 (benralizumab numerically higher), p=0.28 (non-significant).Eosinophil ≥150 cells/µL subgroup: exacerbation-rate result vs placebo (significance): Per-protocol subgroup n=54 (66%): 26 placebo, 28 benralizumab. Reduction in exacerbation rate with benralizumab vs placebo non-significant, p=0.84. No explicit rate-ratio point estimate or CI given in accessible text. Eosinophil ≥200 cells/µL subgroup: exacerbation-rate result vs placebo (significance): Per-protocol subgroup ≥200 cells/µL: n=39/82 (48%), placebo 20 and benralizumab 19; exacerbation-rate reduction was not significant, P=0.26.Eosinophil ≥300 cells/µL subgroup: exacerbation-rate result vs placebo (significance): Per-protocol subgroup ≥300 cells/µL: n=21/82 (26%), placebo 7 and benralizumab 14; exacerbation-rate reduction was not significant, p=0.28. Exacerbation: rate ratio/rate reduction vs placebo, 95% CI: Reported as rate reduction (not rate ratio): benralizumab vs placebo = −3% (95% CI: −58 to 33), p=0.94, by van Elteren test (primary analysis, per-protocol population); qualitatively corroborated by Poisson regression and negative binomial models, but exact numeric estimates from those secondary models were not present in the extracted text.	PMC5082845	No: transcribed as reported; S1 adds no calculation.	
	Active: benralizumab 100 mg SC: 51 (single active-dose arm; no other doses tested). Placebo: 50 (of 101 total randomized: 51 benralizumab, 50 placebo), ITT population. CT.Gov enrollment field independently confirmed at 421 (a trial-level total, almost certainly reflecting patients screened rather than randomized, given the paper’s own description of substantial screen failure under the sputum-eosinophilia eligibility gate): this is a real but explainable registry/publication mismatch, not an extraction error.	Pre-bronchodilator FEV1: week 56, week 4, week 80, 32 weeks	FEV1 (pre-bronchodilator): mean/LS-mean change from baseline, active arm(s): benralizumab: pre-bronchodilator FEV1 mean change +0.13 L (SD 0.41), baseline to week 56, p=0.014 vs placebo. Post-bronchodilator FEV1: mean change +0.09 L (SD 0.39), p=0.014 vs placebo. Improvements evident by week 4 and sustained to week 80 (32 weeks after last dose); per-protocol population. FEV1 (pre-bronchodilator): mean/LS-mean change from baseline, placebo arm: placebo: pre-bronchodilator FEV1 mean change −0.06 L (SD 0.24), baseline to week 56 (reported as “mean change,” not explicitly labelled LS-mean, though between-group comparisons used ANCOVA per Methods). Post-bronchodilator FEV1: mean change −0.08 L (SD 0.21). Per-protocol population.	Eosinophil ≥150 cells/µL subgroup: FEV1 (pre-bronchodilator) improvement vs placebo (significance): Significant improvement in pre-bronchodilator FEV1 at week 56 with benralizumab vs placebo, p=0.031; no explicit mL value or CI given in extracted text; eosinophil ≥200 cells/µL subgroup: FEV1 (pre-bronchodilator) improvement vs placebo (significance): Statistically significant improvement in pre-bronchodilator FEV1 at week 56 with benralizumab vs placebo, p=0.035. No exact mL value or CI given in accessible text. Eosinophil ≥300 cells/µL subgroup: FEV1 (pre-bronchodilator) improvement vs placebo (significance): Non-significant improvement in pre-bronchodilator FEV1 at week 56 with benralizumab vs placebo, p=0.22. No exact mL value or CI given in accessible text. FEV1 (pre-bronchodilator): difference vs placebo (mL), SE/95% CI: Not obtainable from any available source: the accessible full text gives only per-arm mean changes (with SD) and p=0.014; no explicit between-group LS-mean difference in mL with SE or 95% CI is reported anywhere in the extracted text (not obtainable).	PMC5082845	No: transcribed as reported; S1 adds no calculation.	
	Active: benralizumab 100 mg SC: 51 (single active-dose arm; no other doses tested). Placebo: 50 (of 101 total randomized: 51 benralizumab, 50 placebo), ITT population. CT.Gov enrollment field independently confirmed at 421 (a trial-level total, almost certainly reflecting patients screened rather than randomized, given the paper's own description of substantial screen failure under the sputum-eosinophilia eligibility gate): this is a real but explainable registry/publication mismatch, not an extraction error.	Post-bronchodilator FEV1: week 56	FEV1 (post-bronchodilator): mean/LS-mean change from baseline, active arm(s): benralizumab: post-bronchodilator FEV1 mean change +0.09 L (SD 0.39), baseline to week 56, p=0.014 vs placebo. FEV1 (post-bronchodilator): mean/LS-mean change from baseline, placebo arm: placebo: post-bronchodilator FEV1 mean change −0.08 L (SD 0.21), baseline to week 56, per-protocol population.	FEV1 (post-bronchodilator): difference vs placebo (mL), SE/95% CI: Not obtainable: per-arm post-BD changes are reported, but no explicit between-group difference in mL with SE or 95% CI is supplied (not obtainable).	not obtainable: confirmed: no mL/SE/CI between-group difference reported in extracted text for the post-BD comparison, same as pre-BDPMC5082845	No: transcribed as reported; S1 adds no calculation.	
	Active: benralizumab 100 mg SC: 51 (single active-dose arm; no other doses tested). Placebo: 50 (of 101 total randomized: 51 benralizumab, 50 placebo), ITT population. CT.Gov enrollment field independently confirmed at 421 (a trial-level total, almost certainly reflecting patients screened rather than randomized, given the paper's own description of substantial screen failure under the sputum-eosinophilia eligibility gate): this is a real but explainable registry/publication mismatch, not an extraction error.	SGRQ total score; week 56	SGRQ-C total score: LS-mean/mean change from baseline, active arm(s): benralizumab: mean change −5.51 in SGRQ-C total score, baseline to week 56 (reported as “mean change,” not explicitly labelled LS-mean). Placebo: −4.43. Difference between groups was not statistically significant (no exact p-value or difference figure with CI given in the accessible text).SGRQ-C total score: LS-mean/mean change from baseline, placebo arm: −4.43 (SGRQ-C total score), change from baseline to week 56	Eosinophil subgroup (≥150/≥200/≥300 cells/µL): SGRQ-C and CRQ-SAS numeric estimates vs placebo (qualitative): SGRQ-C change from baseline, mean (SD; n), placebo vs benralizumab: overall −4.43 (11.71;42) vs −5.51 (16.64;37), P=0.739; <200 cells/µL −4.26 (10.17;21) vs −2.10 (16.47;20), P=0.614; ≥200 −4.79 (13.64;20) vs −9.52 (16.42;17), P=0.345; <300 −4.40 (11.04;34) vs −1.90 (14.97;25), P=0.463; ≥300 −5.11 (16.24;7) vs −13.03 (18.08;12), P=0.354. CRQ-SAS numeric subgroup estimates remain unavailable. SGRQ-C: LS-mean/mean difference vs placebo, SE/95% CI: Not obtainable: per-arm SGRQ-C changes and narrative nonsignificance are reported, but no explicit between-group difference with SE or 95% CI is supplied (not obtainable).	PMC5082845not obtainable: confirmed: text states only that “no significant differences between treatment groups were noted” for SGRQ-C/CRQ-SAS/BODE; no exp…	No: transcribed as reported; S1 adds no calculation.	
	Active: benralizumab 100 mg SC: 51 (single active-dose arm; no other doses tested). Placebo: 50 (of 101 total randomized: 51 benralizumab, 50 placebo), ITT population. CT.Gov enrollment field independently confirmed at 421 (a trial-level total, almost certainly reflecting patients screened rather than randomized, given the paper’s own description of substantial screen failure under the sputum-eosinophilia eligibility gate): this is a real but explainable registry/publication mismatch, not an extraction error.	E-RS:COPD. Outcome-specific timepoint NR; trial follow-up context: Dosing period 48 weeks (8 doses); efficacy assessments (including the primary endpoint) through week 56; safety monitoring continued a further 24 weeks (to week 80), i.e., 32 weeks after the last dose (week 48).	E-RS: LS-mean change from baseline, active arm(s): Not obtainable: E-RS was not used; this trial used EXACT-PRO (not obtainable). E-RS: LS-mean change from baseline, placebo arm: Not obtainable: E-RS was not used; this trial used EXACT-PRO (not obtainable).	E-RS: LS-mean difference vs placebo, SE/95% CI: Not obtainable: E-RS was not used; this trial used EXACT-PRO (not obtainable).	not obtainable: same reason (E-RS not used; trial used EXACT-PRO)not obtainable: confirmed this trial did not use E-RS; it used EXACT-PRO as an exploratory endpoint instead, and exploratory numeric results were…not obtainable: E-RS not used in this trial	Not applicable: NR/not obtainable.	
	Active: benralizumab 100 mg SC: 51 (single active-dose arm; no other doses tested). Placebo: 50 (of 101 total randomized: 51 benralizumab, 50 placebo), ITT population. CT.Gov enrollment field independently confirmed at 421 (a trial-level total, almost certainly reflecting patients screened rather than randomized, given the paper’s own description of substantial screen failure under the sputum-eosinophilia eligibility gate): this is a real but explainable registry/publication mismatch, not an extraction error.	Time to first exacerbation. Outcome-specific timepoint NR; trial follow-up context: Dosing period 48 weeks (8 doses); efficacy assessments (including the primary endpoint) through week 56; safety monitoring continued a further 24 weeks (to week 80), i.e., 32 weeks after the last dose (week 48).	NR/not obtainable: no trial-specific numeric result for this domain was present in the extraction workbook.	NR/not obtainable: no trial-specific numeric result for this domain was present in the extraction workbook.	Extraction workbook (no matching trial-specific outcome row).	Not applicable: NR/not obtainable.	
	Active: benralizumab 100 mg SC: 51 (single active-dose arm; no other doses tested). Placebo: 50 (of 101 total randomized: 51 benralizumab, 50 placebo), ITT population. CT.Gov enrollment field independently confirmed at 421 (a trial-level total, almost certainly reflecting patients screened rather than randomized, given the paper’s own description of substantial screen failure under the sputum-eosinophilia eligibility gate): this is a real but explainable registry/publication mismatch, not an extraction error.	Rescue-medication use. Outcome-specific timepoint NR; trial follow-up context: Dosing period 48 weeks (8 doses); efficacy assessments (including the primary endpoint) through week 56; safety monitoring continued a further 24 weeks (to week 80), i.e., 32 weeks after the last dose (week 48).	NR/not obtainable: no trial-specific numeric result for this domain was present in the extraction workbook.	NR/not obtainable: no trial-specific numeric result for this domain was present in the extraction workbook.	Extraction workbook (no matching trial-specific outcome row).	Not applicable: NR/not obtainable.	
	Active: benralizumab 100 mg SC: 51 (single active-dose arm; no other doses tested). Placebo: 50 (of 101 total randomized: 51 benralizumab, 50 placebo), ITT population. CT.Gov enrollment field independently confirmed at 421 (a trial-level total, almost certainly reflecting patients screened rather than randomized, given the paper’s own description of substantial screen failure under the sputum-eosinophilia eligibility gate): this is a real but explainable registry/publication mismatch, not an extraction error.	Any treatment-emergent adverse event. Outcome-specific timepoint NR; trial follow-up context: Dosing period 48 weeks (8 doses); efficacy assessments (including the primary endpoint) through week 56; safety monitoring continued a further 24 weeks (to week 80), i.e., 32 weeks after the last dose (week 48).	Safety: TEAE, overall n/N per arm (any TEAE): TEAEs : placebo=41; benralizumab 100 mg=45 (n/N = 41/50 placebo, 45/51 benralizumab, N from participant flow STARTED)	NR/not obtainable: no comparative effect with precision was present; see per-arm summary.	PMC5082845	No: transcribed as reported; S1 adds no calculation.	
	Active: benralizumab 100 mg SC: 51 (single active-dose arm; no other doses tested). Placebo: 50 (of 101 total randomized: 51 benralizumab, 50 placebo), ITT population. CT.Gov enrollment field independently confirmed at 421 (a trial-level total, almost certainly reflecting patients screened rather than randomized, given the paper’s own description of substantial screen failure under the sputum-eosinophilia eligibility gate): this is a real but explainable registry/publication mismatch, not an extraction error.	Serious adverse events. Outcome-specific timepoint NR; trial follow-up context: dosing period 48 weeks (8 doses); efficacy assessments (including the primary endpoint) through week 56; safety monitoring continued a further 24 weeks (to week 80), i.e., 32 weeks after the last dose (week 48).	Safety: SAE, n/N per arm: Serious TEAEs: benralizumab 14/51 (27%) vs placebo 9/50 (18%); numerically higher with benralizumab; none investigator-assessed as treatment-related.	NR/not obtainable: no comparative effect with precision was present; see per-arm summary.	PMC5082845; PMID 25208464	No: transcribed as reported; S1 adds no calculation.	
	Active: benralizumab 100 mg SC: 51 (single active-dose arm; no other doses tested). Placebo: 50 (of 101 total randomized: 51 benralizumab, 50 placebo), ITT population. CT.Gov enrollment field independently confirmed at 421 (a trial-level total, almost certainly reflecting patients screened rather than randomized, given the paper’s own description of substantial screen failure under the sputum-eosinophilia eligibility gate): this is a real but explainable registry/publication mismatch, not an extraction error.	AEs leading to discontinuation. Outcome-specific timepoint NR; trial follow-up context: Dosing period 48 weeks (8 doses); efficacy assessments (including the primary endpoint) through week 56; safety monitoring continued a further 24 weeks (to week 80), i.e., 32 weeks after the last dose (week 48).	Safety: discontinuation due to AE, n/N per arm: Treatment-emergent adverse events leading to discontinuation: placebo 2/50; benralizumab 3/51.	NR/not obtainable: no comparative effect with precision was present; see per-arm summary.	PMC5082845	No: transcribed as reported; S1 adds no calculation.	
	Active: benralizumab 100 mg SC: 51 (single active-dose arm; no other doses tested). Placebo: 50 (of 101 total randomized: 51 benralizumab, 50 placebo), ITT population. CT.Gov enrollment field independently confirmed at 421 (a trial-level total, almost certainly reflecting patients screened rather than randomized, given the paper’s own description of substantial screen failure under the sputum-eosinophilia eligibility gate): this is a real but explainable registry/publication mismatch, not an extraction error.	All-cause death. Outcome-specific timepoint NR; trial follow-up context: Dosing period 48 weeks (8 doses); efficacy assessments (including the primary endpoint) through week 56; safety monitoring continued a further 24 weeks (to week 80), i.e., 32 weeks after the last dose (week 48).	Safety: death, n/N per arm: benralizumab: 2/51 (4%) deaths: 1 myocardial infarction, 1 sudden death of unknown cause, neither treatment-related. Placebo: 1/50 (2%) death: bronchitis, occurring after study completion, not treatment-related.	NR/not obtainable: no comparative effect with precision was present; see per-arm summary.	PMC5082845	No: transcribed as reported; S1 adds no calculation.	
	Active: benralizumab 100 mg SC: 51 (single active-dose arm; no other doses tested). Placebo: 50 (of 101 total randomized: 51 benralizumab, 50 placebo), ITT population. CT.Gov enrollment field independently confirmed at 421 (a trial-level total, almost certainly reflecting patients screened rather than randomized, given the paper’s own description of substantial screen failure under the sputum-eosinophilia eligibility gate): this is a real but explainable registry/publication mismatch, not an extraction error.	AEs of special interest. Outcome-specific timepoint NR; trial follow-up context: Dosing period 48 weeks (8 doses); efficacy assessments (including the primary endpoint) through week 56; safety monitoring continued a further 24 weeks (to week 80), i.e., 32 weeks after the last dose (week 48).	NR/not obtainable: no trial-specific numeric result for this domain was present in the extraction workbook.	NR/not obtainable: no trial-specific numeric result for this domain was present in the extraction workbook.	Extraction workbook (no matching trial-specific outcome row).	Not applicable: NR/not obtainable.	
S1.15 COPD-HELP (NCT04075331) [27]							
COPD-HELPCOPD-HELP	Active: 119 (mepolizumab arm, STARTED milestone). Placebo: 119 (Placebo arm, STARTED milestone)	Moderate/severe exacerbation rate48 weeks, 48-week	Exacerbation: adjusted mean count over 48 weeks (as-reported, not annualized), active arm(s) (mepolizumab): 2.80 (95% CI: 2.36-3.23): adjusted mean number of moderate-or-severe exacerbations over 48 weeks, mepolizumab arm (explicitly not an annualized per-patient-year rate). Exacerbation: adjusted mean count over 48 weeks (as-reported, not annualized), placebo arm: 3.45 (95% CI: 2.94-3.95); adjusted mean number of moderate-or-severe exacerbations over 48 weeks, placebo arm (explicitly not an annualized per-patient-year rate). Exacerbation: adjusted mean number of moderate-or-severe exacerbations over 48 weeks, mepolizumab arm: adjusted mean number of moderate-or-severe exacerbations over 48 weeks, mepolizumab arm: 2.80 (95% CI: 2.36-3.23). This is the as-reported 48-week adjusted mean count, NOT a per-patient-year annualized rate: no annualized rate is reported in the abstract or the CT.Gov record. Exacerbation: adjusted mean number of moderate-or-severe exacerbations over 48 weeks, placebo arm: adjusted mean number of moderate-or-severe exacerbations over 48 weeks, placebo arm: 3.45 (95% CI: 2.94-3.95). This is the as-reported 48-week adjusted mean count, NOT a per-patient-year annualized rate.	Exacerbation: rate/risk ratio vs placebo, 95% CI: Risk ratio 0.81 (95% CI: 0.66-1.00) for moderate-or-severe exacerbations, mepolizumab vs placebo, over 48 weeks; upper CI bound reaches the null	PMID 40305842Abstract: ‘risk ratio 0.81; 95% CI, 0.66 to 1.00’	No: transcribed as reported; S1 adds no calculation.	
	Active: 119 (mepolizumab arm, STARTED milestone). Placebo: 119 (Placebo arm, STARTED milestone)	Pre-bronchodilator FEV1. Outcome-specific timepoint NR; trial follow-up context: 48-week treatment/follow-up period, monthly (every-4-week) dosing; absolute maximum 49 weeks allowing the ±1 week visit window at week 48. Secondary-outcome assessments at weeks 0, 4, 8, 12, 24, 36, 48.	FEV1: LS-mean change from baseline, active arm(s): NR/not obtainable: workbook value cell is empty (not obtainable) FEV1: LS-mean change from baseline, placebo arm: NR/not obtainable: workbook value cell is empty (not obtainable)	FEV1: LS-mean difference vs placebo (mL), SE/95% CI: NR/not obtainable: workbook value cell is empty (not obtainable)	Same re-check as aboveClinicalTrials.gov NCT04075331	Not applicable: NR/not obtainable.	
	Active: 119 (mepolizumab arm, STARTED milestone). Placebo: 119 (Placebo arm, STARTED milestone)	Post-bronchodilator FEV1. Outcome-specific timepoint NR; trial follow-up context: 48-week treatment/follow-up period, monthly (every-4-week) dosing; absolute maximum 49 weeks allowing the ±1 week visit window at week 48. Secondary-outcome assessments at weeks 0, 4, 8, 12, 24, 36, 48.	NR/not obtainable: no trial-specific numeric result for this domain was present in the extraction workbook.	NR/not obtainable: no trial-specific numeric result for this domain was present in the extraction workbook.	Extraction workbook (no matching trial-specific outcome row).	Not applicable: NR/not obtainable.	
	Active: 119 (mepolizumab arm, STARTED milestone). Placebo: 119 (placebo arm, STARTED milestone)	SGRQ total score; week 4, week 8, week 12, week 24, week 36, week 48	SGRQ: raw arm mean score by visit, active arm: mepolizumab raw mean (SD), n: baseline 69.8 (15.7), 111; week 4 65.5 (16.0), 102; week 8 64.3 (15.9), 86; week 12 64.3 (16.4), 79; week 24 64.3 (18.0), 73; week 36 61.2 (18.1), 60; week 48 60.7 (19.1), 67. These are raw scores, not LS-mean changes. SGRQ: raw arm mean score by visit, placebo arm: placebo raw mean (SD), n: baseline 72.0 (13.8), 117; week 4 66.2 (16.4), 95; week 8 64.6 (18.1), 84; week 12 63.3 (16.3), 85; week 24 61.7 (18.2), 76; week 36 60.4 (20.0), 67; week 48 62.4 (18.9), 77. These are raw scores, not LS-mean changes.	SGRQ: LS-mean difference vs placebo, SE/95% CI: week-48 SGRQ Mean Difference (Net), mepolizumab vs placebo: 0.57 (95% CI: -2.63 to 3.78), p=0.726 - from a mixed-effects model adjusting for baseline MRC dyspnea, prior hospitalization, visit, and baseline SGRQ.	ClinicalTrials.gov NCT04075331	No: transcribed as reported; S1 adds no calculation.	
	Active: 119 (mepolizumab arm, STARTED milestone). Placebo: 119 (Placebo arm, STARTED milestone)	E-RS:COPD. Outcome-specific timepoint NR; trial follow-up context: 48-week treatment/follow-up period, monthly (every-4-week) dosing; absolute maximum 49 weeks allowing the ±1 week visit window at week 48. Secondary-outcome assessments at weeks 0, 4, 8, 12, 24, 36, 48.	E-RS: LS-mean change from baseline, active arm(s): NR/not obtainable: workbook value cell is empty (not obtainable). E-RS: LS-mean change from baseline, placebo arm: NR/not obtainable: workbook value cell is empty (not obtainable).	E-RS: LS-mean difference vs placebo, SE/95% CI: NR/not obtainable: workbook value cell is empty (not obtainable).	Same re-check as aboveClinicalTrials.gov NCT04075331	Not applicable: NR/not obtainable.	
	Active: 119 (mepolizumab arm, STARTED milestone). Placebo: 119 (placebo arm, STARTED milestone)	Time to first exacerbation. Outcome-specific timepoint NR; trial follow-up context: 48-week treatment/follow-up period, monthly (every 4-week) dosing; absolute maximum 49 weeks allowing the ±1 week visit window at week 48. Secondary-outcome assessments at weeks 0, 4, 8, 12, 24, 36, 48.	NR/not obtainable: no trial-specific numeric result for this domain was present in the extraction workbook.	NR/not obtainable: no trial-specific numeric result for this domain was present in the extraction workbook.	Extraction workbook (no matching trial-specific outcome row).	Not applicable: NR/not obtainable.	
	Active: 119 (mepolizumab arm, STARTED milestone). Placebo: 119 (Placebo arm, STARTED milestone)	Rescue-medication use. Outcome-specific timepoint NR; trial follow-up context: 48-week treatment/follow-up period, monthly (every-4-week) dosing; absolute maximum 49 weeks allowing the ±1 week visit window at week 48. Secondary-outcome assessments at weeks 0, 4, 8, 12, 24, 36, 48.	NR/not obtainable: no trial-specific numeric result for this domain was present in the extraction workbook.	NR/not obtainable: no trial-specific numeric result for this domain was present in the extraction workbook.	Extraction workbook (no matching trial-specific outcome row).	Not applicable: NR/not obtainable.	
	Active: 119 (mepolizumab arm, STARTED milestone). Placebo: 119 (Placebo arm, STARTED milestone)	Any treatment-emergent adverse event. Outcome-specific timepoint NR; trial follow-up context: 48-week treatment/follow-up period, monthly (every-4-week) dosing; absolute maximum 49 weeks allowing the ±1 week visit window at week 48. Secondary-outcome assessments at weeks 0, 4, 8, 12, 24, 36, 48.	Safety: TEAE, n/N per arm: placebo 91/119 with >=1 AE; mepolizumab 91/118 with >=1 AE; incidence rate ratio 1.24 (95% CI: 0.98-1.56), p=0.070.	NR/not obtainable: no comparative effect with precision was present; see per-arm summary.	ClinicalTrials.gov NCT04075331	No: transcribed as reported; S1 adds no calculation.	
	Active: 119 (mepolizumab arm, STARTED milestone). Placebo: 119 (Placebo arm, STARTED milestone)	Serious adverse events. Outcome-specific timepoint NR; trial follow-up context: 48-week treatment/follow-up period, monthly (every-4-week) dosing; absolute maximum 49 weeks allowing the ±1 week visit window at week 48. Secondary-outcome assessments at weeks 0, 4, 8, 12, 24, 36, 48.	Safety: SAE, n/N per arm: placebo 3/119 with >=1 SAE (116/119 without); mepolizumab 2/118 with >=1 SAE (116/118 without); incidence rate ratio 0.69 (95% CI: 0.12-4.14), p=0.686. Safety population denominators 119/118 (differs from the randomized 119/119 by one mepolizumab-arm participant).	NR/not obtainable: no comparative effect with precision was present; see per-arm summary.	ClinicalTrials.gov NCT04075331	No: transcribed as reported; S1 adds no calculation.	
	Active: 119 (mepolizumab arm, STARTED milestone). Placebo: 119 (Placebo arm, STARTED milestone)	AEs leading to discontinuation. Outcome-specific timepoint NR; trial follow-up context: 48-week treatment/follow-up period, monthly (every-4-week) dosing; absolute maximum 49 weeks allowing the ±1 week visit window at week 48. Secondary-outcome assessments at weeks 0, 4, 8, 12, 24, 36, 48.	Safety: discontinuation due to AE, n/N per arm: NR/not obtainable: workbook value cell is empty (not obtainable).	NR/not obtainable: no comparative effect with precision was present; see per-arm summary.	ClinicalTrials.gov NCT04075331	Not applicable: NR/not obtainable.	
	Active: 119 (mepolizumab arm, STARTED milestone). Placebo: 119 (Placebo arm, STARTED milestone)	All-cause death. Outcome-specific timepoint NR; trial follow-up context: 48-week treatment/follow-up period, monthly (every-4-week) dosing; absolute maximum 49 weeks allowing the ±1 week visit window at week 48. Secondary-outcome assessments at weeks 0, 4, 8, 12, 24, 36, 48.	Safety: death, n/N per arm: All-cause death: placebo 11/119, mepolizumab 9/119 (HR 0.84, 95% CI: 0.35-2.04, p=0.705). Respiratory-cause death: placebo 8/119, mepolizumab 5/119 (HR 0.64, 95% CI: 0.21-1.95, p=0.432).	NR/not obtainable: no comparative effect with precision was present; see per-arm summary.	ClinicalTrials.gov NCT04075331	No: transcribed as reported; S1 adds no calculation.	
	Active: 119 (mepolizumab arm, STARTED milestone). Placebo: 119 (Placebo arm, STARTED milestone)	AEs of special interest. Outcome-specific timepoint NR; trial follow-up context: 48-week treatment/follow-up period, monthly (every-4-week) dosing; absolute maximum 49 weeks allowing the ±1 week visit window at week 48. Secondary-outcome assessments at weeks 0, 4, 8, 12, 24, 36, 48.	NR/not obtainable: no trial-specific numeric result for this domain was present in the extraction workbook.	NR/not obtainable: no trial-specific numeric result for this domain was present in the extraction workbook.	Extraction workbook (no matching trial-specific outcome row).	Not applicable: NR/not obtainable.	

Certainty of evidence: GRADE-CINeMA certainty was summarised in Table 6 and is set out domain by domain in Table 7. Certainty was "Moderate" for dupilumab vs placebo (downgraded for indirectness, effect derives from an eosinophil-enriched population) and "Low" for astegolimab (high RoB; dose-schedule-dependent significance), mepolizumab ("Some concerns"; within-node eosinophil-population mixing) and benralizumab (imprecision, CI includes no effect; "Some concerns"; ≥220 subgroup). Across the network, incoherence is not evaluable (star geometry) and the cross-agent ranking of the three IL-5/5Rα/ST2 agents is of low certainty.

**Table 6:** Summary of findings - biologics versus placebo for moderate/severe COPD exacerbations (primary maintenance network) Certainty by applying the CINeMA/GRADE-NMA framework [16] to the network outputs and RoB 2 judgments; full reasoning in Table 7. Range of equivalence for imprecision RR: 0.80-1.25. Patients: adults with COPD on maintenance inhaled therapy. Intervention: biologic added to background therapy. Comparison: placebo. Outcome: annualized rate of moderate/severe COPD exacerbations (rate ratio), maintenance network k = 9 RCTs, random-effects netmeta. *Illustrative absolute effects assume a placebo rate of 1.00 event/patient-year (a round anchor within the trials' placebo range ≈1.0-1.4/yr). GRADE: ⊕⊕⊕⊕ High · ⊕⊕⊕◯ Moderate · ⊕⊕◯◯ Low · ⊕◯◯◯ Very low. Overarching caveats: star geometry means incoherence is not evaluable and the cross-agent ranking rests on untestable transitivity; eosinophil-population mixing is the leading indirectness threat; reporting bias could not be assessed statistically. Dupilumab's top rank is robust across all sensitivity analyses; the astegolimab/mepolizumab/benralizumab ordering is not robust ("clustered, order uncertain").

Comparison	Network RR (95% CI)	Illustrative absolute effect per 1000 patient-years*	Direct evidence (RoB 2)	GRADE-CINeMA certainty	Main downgrade reasons
Dupilumab vs placebo	0.68 (0.59-0.79)	319 fewer (210-413 fewer)	BOREAS, NOTUS (both Low)	⊕⊕⊕◯ Moderate	Indirectness −1: effect derives entirely from an eosinophil-enriched (≥300 cells/µL) population; applicability to eosinophil-low COPD uncertain.
Astegolimab vs placebo	0.85 (0.75-0.96)	150 fewer (37-250 fewer)	ALIENTO, ARNASA (both High)	⊕⊕◯◯ Low	Within-study bias −1 (both nodes High RoB); Imprecision/robustness −1 (significance is dose-schedule-dependent - non-significant under the Q4W-for-both convention, Sensitivity 6).
Mepolizumab vs placebo	0.88 (0.79-0.97)	124 fewer (29-209 fewer)	METREX, METREO, MATINEE (Some concerns ×3)	⊕⊕◯◯ Low	Within-study bias −1 (Some concerns ×3); Indirectness −1 (node pools fundamentally different eosinophil populations - unselected METREX RR 0.98 vs enriched METREO/MATINEE RR: 0.79-0.80 - and MATINEE’s variable up-to-104-wk window).
Benralizumab vs placebo	0.88 (0.77-1.01)	118 fewer (228 fewer to 7 more)	GALATHEA, TERRANOVA (Some concerns ×2)	⊕⊕◯◯ Low	Imprecision −1 (CI includes no effect); Within-study bias / indirectness −1 (Some concerns ×2; estimate restricted to the EOS ≥220/µL primary-analysis subgroup, not the whole randomized population).

**Table 7:** GRADE-CINeMA assessment Grading of Recommendations Assessment, Development and Evaluation (GRADE) via Confidence in Network Meta-Analysis (CINeMA) (GRADE-CINeMA) assessment [16] - certainty of the network estimates for moderate/severe exacerbations versus placebo. Six CINeMA domains assessed per comparison: within-study bias, reporting bias, indirectness, imprecision (range of equivalence: 0.80-1.25), heterogeneity and incoherence. RR and 95% CI as in Table 2; PI = 95% prediction interval. Incoherence is not evaluable for any comparison because the star network contains no closed loop. Certainty symbols: ⊕⊕⊕ Moderate, ⊕⊕ Low. Summary ratings are in Table 6; the full six-domain reasoning is here. Source data were deposited online.

Comparison (RR, 95% CI, 95% PI)	Within-study bias	Reporting bias	Indirectness	Imprecision (ROE 0.80–1.25)	Heterogeneity	Incoherence	Certainty
Dupilumab vs placebo - 0.68 (0.59-0.79); PI 0.55-0.84	No concerns - 100%. Low-RoB (BOREAS, NOTUS)	No concerns (primary outcome fully reported; not statistically testable)	Some concerns - evidence entirely from an eosinophil-enriched (≥300/µL) population; applicability to eosinophil-low COPD uncertain	No concerns - whole CI below 0.80 (clear important benefit)	No concerns - I²=9.68%, 2 consistent trials (0.70/0.66)	Not evaluable (star)	⊕⊕⊕◯ MODERATE (−1 indirectness)
Astegolimab vs placebo - 0.85 (0.75-0.96); PI 0.71-1.02	Major concerns - 100% High-RoB (ALIENTO, ARNASA)	No concerns	No concerns - eosinophil-unselected (broad applicability)	Some concerns - point estimate 0.85 lies inside the ROE; CI crosses 0.80; significance is dose-schedule-fragile (NS under Q4W, Sensitivity 6)	Some concerns - PI crosses null	Not evaluable	⊕⊕◯◯ LOW (−1 RoB, −1 imprecision; borderline Very Low given High RoB)
Mepolizumab vs placebo - 0.88 (0.79-0.97); PI 0.75–1.02	Some concerns - 100%. Some concerns (METREX, METREO, MATINEE)	No concerns	Some concerns - node pools distinct populations (unselected METREX 0.98 vs enriched METREO/MATINEE 0.79-0.80) + MATINEE variable up-to-104-wk window	Some concerns - point 0.88 inside ROE; CI crosses 0.80	Some concerns - real within-node heterogeneity (same driver as indirectness)	Not evaluable	⊕⊕◯◯ LOW (−1 RoB, −1 indirectness/heterogeneity)
Benralizumab vs placebo - 0.88 (0.77-1.01); PI 0.73-1.07	Some concerns - 100%. Some concerns (GALATHEA, TERRANOVA)	No concerns	Some concerns - estimate is the EOS ≥220/µL primary-analysis subgroup, not the whole randomized population	Major concerns - CI includes no effect (1.01) and crosses 0.80	Some concerns - PI crosses null	Not evaluable	⊕⊕◯◯ LOW (−1 RoB, −1 imprecision)

Table 7 records the six CINeMA domains - within-study bias, reporting bias, indirectness, imprecision against a range of equivalence of 0.80-1.25, heterogeneity and incoherence - for each of the four comparisons, together with the point estimate, confidence interval, prediction interval and the resulting certainty rating with its explicit reasons for downgrading. It shows that no two agents are downgraded for the same reason, which is why the ratings should not be read as a single quality gradient. Dupilumab is "Moderate," downgraded once and only for indirectness: its two trials are 100% "Low" RoB, the primary outcome is fully reported, heterogeneity is low across two consistent trials (0.70 and 0.66), and the whole confidence interval lies below 0.80, but the evidence comes entirely from an eosinophil-enriched population (≥300 cells/µL), so applicability to eosinophil-low COPD is uncertain. Astegolimab is "Low," downgraded for within-study bias (both contributing trials "High" RoB) and for imprecision, since the point estimate of 0.85 lies inside the range of equivalence and its significance is dose-schedule fragile, becoming non-significant under the Q4W substitution in Table 4. Its indirectness is the least concerning in the network, because the astegolimab trials are eosinophil-unselected. Mepolizumab is "Low," downgraded for within-study bias and for combined indirectness and heterogeneity: the node pools materially different populations, the eosinophil-unselected METREX at 0.98 against the enriched METREO and MATINEE at 0.79-0.80, with MATINEE contributing a variable up to a 104-week window. Benralizumab is "Low," downgraded for within-study bias and for imprecision, its confidence interval including no effect at 1.01, and further limited because the estimate is drawn from the eosinophil ≥220 cells/µL primary analysis subgroup rather than the whole randomized population. Reporting bias raised no concern for any comparison, since the primary annualized exacerbation rate was consistently reported in the registered pivotal trials. Incoherence is not evaluable for any comparison because the network is star-shaped and contains no closed loop, and the cross-agent ranking of the three IL-5, IL-5Rα, and ST2 agents is accordingly of low certainty.

Discussion

Summary of Evidence

Across completed COPD biologic RCTs reporting extractable exacerbation data, four agents reduced moderate/severe exacerbations versus placebo. Dupilumab had the largest estimated relative reduction and highest P-score (≈32% relative reduction; RR: 0.68) and was favored over each other agent in indirect network comparisons via the common placebo. Astegolimab, mepolizumab, and benralizumab produced smaller, closely clustered reductions (RR: 0.85-0.88) whose relative order was not robustly established. Dupilumab remained first across every sensitivity analysis and all three analytic engines; the complete ordering did not, because astegolimab and mepolizumab exchanged positions under the dose-schedule sensitivity.

Which Biologic, For Which Patient

The ranking is inseparable from population. Dupilumab's benefit derives entirely from an eosinophil-enriched (≥300 cells/µL) population and should be read as the option with the largest estimated benefit for that phenotype; its applicability to eosinophil-low COPD is untested here. The astegolimab (ST2) trials, by contrast, enrolled eosinophil-agnostically, so astegolimab's more modest effect speaks to an unselected population and to an alarmin mechanism that is largely eosinophil-independent, a pattern confirmed in the prespecified pooled ALIENTO+ARNASA analysis, which reported consistent exacerbation reduction irrespective of baseline eosinophil count [41]. Mepolizumab's node spans an unselected trial (METREX, RR: 0.98) and enriched trials (METREO/MATINEE, RR: 0.79-0.80), underscoring that eosinophil selection, not agent identity alone, drives much of the apparent between-agent difference. The prespecified eosinophil-stratified networks were not estimable because available subgroup results use incompatible thresholds, pooled versus trial-specific reporting, and sparse node coverage; this remains a priority for an update.

Comparison With Prior Reviews

Pitre et al. conducted a frequentist COPD biologics network meta-analysis with GRADE through July 2024 and reported a dupilumab RR of 0.68 [16], closely reproduced here. The incremental contribution of this review is the later 2025-2026 evidence wave, broader seven-agent/three-pathway scope, and explicit eosinophil transitivity and CINeMA assessment, not the first certainty-rated cross-agent NMA. Other relevant syntheses include the type 2 network analysis [42], the number-needed-to-treat review [43], a living review [44], the Matera et al. multi-criteria decision analysis [45], and a registered dupilumab-specific review protocol [46]. The planned eosinophil-stratified NMA remained unestimable.

Limitations

The following are the limitations of the study: (1) Star network, no head-to-head biologic trials, so no closed loops; inconsistency cannot be tested, and all active-active comparisons are indirect through placebo, resting on an untestable transitivity assumption. (2) Eosinophil-population mixing across nodes is a known effect modifier and the leading indirectness threat; the stratified networks that would mitigate it were not estimable. (3) The search was restricted to free-access sources because Embase/Scopus/WoS were unavailable, which may reduce retrieval completeness; fresh registry recovery improved secondary/safety coverage, but inconsistent definitions, timepoints, thresholds, and sparse comparable contrasts meant that those outcomes remained narrative, and some fields were genuinely unavailable. (4) A 33-record ICTRP export gap occurred before screening. (5) Duration heterogeneity (MATINEE up to 104 weeks) and the astegolimab dose-schedule choice affect the lower ranks. (6) The multinma corroborative fit had 11 divergent transitions despite acceptable R-hat and agreement with netmeta/gemtc. (7) Five pivotal phase 3 programs (AERIFY-1/2, TITANIA, OBERON, RESOLUTE) were results-pending at the search date and remained unpublished at update searches through 2026-07-18.

Implications and Ongoing Trials

For the eosinophil-high, exacerbation-prone patient already on triple therapy, dupilumab currently has the strongest and most certain exacerbation-reduction evidence. For eosinophil-unselected patients, the ST2 and IL-5-pathway options offer smaller benefits of lower certainty. Imminent read-outs (itepekimab AERIFY-1/2, tozorakimab TITANIA/OBERON and MIRANDA, benralizumab RESOLUTE, and dupilumab phase 4 trials) will connect and re-rank the alarmin nodes. A pre-submission update search, most recently repeated on 2026-07-18, confirmed that AERIFY-1 (NCT04701983), AERIFY-2 (NCT04751487), TITANIA (NCT05158387), OBERON (NCT05166889), and RESOLUTE (NCT04053634) are all marked completed on ClinicalTrials.gov, but none has posted registry results or a peer-reviewed primary publication, so all five remain E6 and the included set is unchanged; a further update immediately before submission is advised.

## Conclusions

In a placebo-anchored network meta-analysis of completed COPD biologic RCTs, dupilumab showed the largest estimated reduction in moderate/severe exacerbations within the available indirect network evidence (moderate certainty, eosinophil-enriched population), while astegolimab, mepolizumab, and benralizumab produced smaller, similar reductions of low certainty and uncertain relative order. The comparative evidence is constrained by a star-shaped network and by eosinophil-population heterogeneity; head-to-head and eosinophil-stratified data are needed to establish the relative ranking of biologics for COPD and to extend these findings to eosinophil-unselected patients.

## Appendices

**Table 8:** PRISMA 2020 checklist ☑ content present in this manuscript and its supplementary materials.

#	Item	Reported?	Location
1	Title (identifies as SR + NMA)	☑	Title
2	Abstract (structured)	☑	Abstract
3	Rationale	☑	§1 Introduction
4	Objectives	☑	§1 (Objective); §2.2
5	Eligibility criteria	☑	§2.2 (I1–I7/E1–E7)
6	Information sources	☑	§2.3
7	Search strategy (full)	☑	§2.3; full strings in the search log (PRISMA-S)
8	Selection process	☑	§2.4 (dual blinded screening with human adjudication; deviations #3–#4)
9	Data collection process	☑	§2.5 (dual independent extraction with reconciliation; deviation #5)
10a	Outcomes sought	☑	§2.2; §2.5 — all compatible prespecified outcomes, doses and time points sought; selection rule stated
10b	Other variables sought	☑	§2.5 — population, intervention, design, funding/COI and effect-modifier variables; missing values not imputed
11	Risk of bias assessment	☑	§2.6 (RoB 2; deviation #6); κ 0.464
12	Effect measures	☑	§2.7
13a	Eligibility for each synthesis	☑	§2.5; §2.8
13b	Data preparation	☑	§2.7 — log transformation and interval-derived standard errors
13c	Display methods	☑	§2.8; Figures 2, 5; Tables 2-3
13d	Synthesis methods and rationale	☑	§2.8 — generic inverse variance, random effects, common DerSimonian–Laird heterogeneity, netmeta 3.3.1
13e	Exploration of heterogeneity	☑	§2.8–§2.9 — phase, model, duration, eosinophil and pathway analyses; rank-deficient analysis disclosed
13f	Sensitivity analyses	☑	§2.8; §3.6 — six sensitivity families and two Bayesian engines
14	Reporting bias assessment	☑	§2.9; §3.7 (not estimable — no contrast ≥10 studies; narrative)
15	Certainty assessment (GRADE)	☑	§2.10; §3.8; Table 6; six-domain reasoning in Table 7 (CINeMA/GRADE-NMA framework; assessment-time ROE 0.80–1.25, disclosed as not protocol-pre-specified)
16	Study selection results + flow diagram	☑	§3.1; Figure 1
17	Study characteristics	☑	§3.2; Table 1
18	Risk of bias in studies	☑	§3.4
19	Results of individual studies	☑	Table 5 covers all 15 trials × 12 prespecified domains with explicit NR/not-obtainable cells
20	Results of syntheses	☑	§3.5–§3.6; Tables 2-3; Figures 2, 5
21	Reporting biases	☑	§3.7 (narrative; registry-vs-publication)
22	Certainty of evidence	☑	§3.8; Tables 6-7 (dupilumab: Moderate; others: Low)
23	Discussion	☑	§4 (summary, comparison, limitations, implications, conclusions)
24a	Registration	☑	§2.1; Declarations — PROSPERO CRD420261448694
24b	Protocol access	☑	§2.1 identifies the registered protocol (PROSPERO CRD420261448694, https://www.crd.york.ac.uk/prospero/); the full protocol (v0.3) is deposited at https://doi.org/10.5281/zenodo.21979837
24c	Amendments	☑	§2.11 documents and explains protocol amendments
25	Support/funding	☑	Declarations — None
26	Competing interests	☑	Declarations — None declared
27	Availability of data/code	☑	Data Availability statement: the protocol, extraction workbook, analysis code, per-trial provenance records, search log, screening decisions, risk-of-bias assessments and CINeMA input are openly deposited at https://doi.org/10.5281/zenodo.21979837 (CC BY 4.0)

**Table 9:** PRISMA 2020 statement and its extension for network meta-analysis (PRISMA-NMA) checklist Additional NMA reporting handled in the core checklist/methods: - Assumptions of the model (random-effects, common heterogeneity, placebo-anchored): §2.8. - Effect measures and how multi-arm/dose arms were handled (one node per agent at the pivotal dose; dose-schedule sensitivity): §2.7–§2.8; §3.6. - Software and engines (netmeta primary; multinma + gemtc Bayesian sensitivity): §2.8; results summary. - Certainty of network estimates via GRADE-CINeMA (per-comparison): §3.8; Tables 6-7.

NMA item	Requirement	Reported?	Location
S1 — Geometry of the network	Describe the network structure; present a network geometry diagram	☑	§3.3; Figure 2 (star: placebo + 4 agents)
S2 — Summary of network geometry	Describe any imbalance/notable features that may bias results	☑	§3.3; §4.4 — star-shaped, no closed loops, no head-to-head trials; three agents connect only via the phase-2 sensitivity layer; tozorakimab not connected
S3 — Summary results for each comparison	Present network estimates for all pairwise comparisons (league table) + ranking	☑	§3.5; Table 2 (RR vs placebo + P-score), Table 3 (full league table)
S4 — Ranking/hierarchy of treatments	Report ranking metrics (P-scores/SUCRA) and interpret cautiously	☑	§3.5 (P-scores); §3.6 (SUCRA 0.97 dupilumab, Bayesian); §4.1 (ranking of the lower three is uncertain)
S5 — Assessment of inconsistency/transitivity	Report inconsistency assessment or explain why not done; assess transitivity	☑	§2.9; §3.7; §4.4 — inconsistency not estimable (star network; transitivity assessed clinically; eosinophil-population mixing disclosed as the leading indirectness threat). The exact “Q=0, df=0” between-designs statistic is stated in Table 2 and Table 7

**Table 10:** PRISMA-S search log PRISMA-S checklist (Rethlefsen 2021) for transparent reporting of the literature search. All 16 PRISMA-S items are presented in one continuous table. Items 6 (Contacts) and 14 (formal information-specialist peer review) were not performed and are transparently reported as such. The complete search record - per-interface strings, execution dates, filters, result counts, the deduplication procedure with record-level output, the registry query procedures including handling of the 33-record WHO ICTRP export gap, and the Google Scholar screening limits - is deposited at https://doi.org/10.5281/zenodo.21979837 so that another investigator can execute the searches rather than reconstruct them. Status: All 16 PRISMA-S items are addressed; 14 are satisfied and 2 (item 6 Contacts and item 14 formal information-specialist peer review) are transparently marked not performed.

#	Item	Reported?	Location/detail
Information sources & methods			
1	Database name	☑	PubMed/MEDLINE (NCBI E-utilities esearch, live index 2026-07-12), Cochrane CENTRAL (Issue 6 of 12, June 2026), Europe PMC (REST search API, resultType=core). Search log §“Execution records” items 1–3.
2	Multi-database searching	☑	3 bibliographic databases + 2 registries searched; the canonical 7-agent synonym set was adapted to each interface’s syntax (exact per-interface strings recorded). Embase, Web of Science, and Scopus were excluded for lack of institutional access (disclosed as a deviation); CENTRAL recovers 168 Embase-sourced records, partly offsetting that exclusion (log §2).
3	Study registries	☑	ClinicalTrials.gov API v2 /studies (Interventional filter, per-agent query.intr, all statuses) → 54 unique records; WHO ICTRP Advanced Search (data through 29 June 2026, all recruitment statuses) → 256 records bridging to 93 unique trials. Log §“Execution records” items 4–5.
4	Online resources and browsing	☑ (supplement)	Google Scholar (signed-in Chrome; 40 hits captured, relevance-ranked) as an uncounted grey-literature supplement; ATS/ERS conference proceedings and manufacturer results records/CSR synopses flagged for screening/extraction. Log §“Execution records” item 6 + item 7.
5	Citation searching	☑	Backward + forward citation chasing of every candidate trial’s primary report and of prior biologic-in-COPD reviews (Pitre 2025; Hu 2025; Wang & Ma 2026; García Morales 2026; Xu 2025 protocol) via OpenAlex/Crossref/Semantic Scholar/scite (24 seeds, 324 related records). Discovery role only — never counted as a PRISMA database. Log §“Discovery layer” (item 7/9). No artificial-intelligence, machine-learning, or automated tools were used for screening, eligibility assessment, data extraction, risk-of-bias assessment, analysis, or synthesis; these were performed manually by two reviewers, and the above platforms were used solely for citation discovery and deduplication.
6	Contacts	☐ not performed	No study authors or subject experts were contacted for unpublished data. Not claimed. Outcome-completeness gaps are instead handled transparently as not obtainable/flagged rows in extraction (126 not-obtainable rows logged).
7	Other methods	☑	Manufacturer (Sanofi/Regeneron, GSK, AstraZeneca, Roche/Genentech) results records and CSR synopses were checked at extraction for outcome completeness (discovery role, uncounted). Log §“Execution records” item 7 / discovery layer.
Search strategies			
8	Full search strategies	☑	The complete, verbatim search string for every source (exactly as run, including PubMed’s parsed translation) is recorded in the log §“Execution records” items 1–5; the log and the raw exports are deposited at https://doi.org/10.5281/zenodo.21979837
9	Limits and restrictions	☑	No date or language limit on any source. Registries limited to Interventional study type. PubMed used the Cochrane HSSS RCT filter (item 10). The only source restriction — free-access databases only — is disclosed as a deviation. Log §“Execution records” (per-source “Filters/limits” lines).
10	Search filters	☑	PubMed: Cochrane Highly Sensitive Search Strategy (sensitivity-maximizing RCT filter, 2008 revision) substituted for the draft [ptyp] line (exact filter string in log §1). CENTRAL: no separate RCT filter (design inherent), All-Text field + word-variation lemmatization.
11	Prior work	☑	Search strings were built from the protocol/eligibility-rubric canonical synonym set and adapted per interface; not copied from a prior published systematic review. Scope-widening from the dupilumab-only draft to all 7 agents is documented.
12	Updates	☑	Pre-submission update search of the five pivotal phase-3 trials run 2026-07-14; to be repeated immediately before submission.
13	Dates of searches	☑	All sources run 2026-07-12; update search 2026-07-14. Database currency: CENTRAL Issue 6 of 12 (June 2026); ICTRP data through 29 June 2026; PubMed/Europe PMC/ClinicalTrials.gov live index 2026-07-12.
Peer review			
14	Peer review	☐ not performed (partial)	The per-database strings were labelled “draft — finalize with an information specialist” and then run verbatim on 2026-07-12; no formal PRESS/information-specialist peer review of the strategy was completed. This is disclosed as a limitation, not claimed as done. Independent count re-verification (a reproducibility check, not a PRESS review) did occur: PubMed 179, Europe PMC 1911, ClinicalTrials.gov 54 were each reproduced exactly by an independent re-derivation (log §“Discovery layer”).
Managing records			
15	Total records	☑	2719 identified pre-dedup = 2679 databases+registers (PubMed 179 · CENTRAL 279 · Europe PMC 1911 · ClinicalTrials.gov 54 · WHO ICTRP 256) + 40 Google Scholar supplement. Per-source counts in log §“Total identified”.
16	Deduplication	☑	365 confirmed duplicates removed + 33 ICTRP export-gap removals → 2321 unique records screened. Method and borderline-pair adjudication documented in the deduplication log.

**Table 11:** PRISMA-for-Abstracts checklist All PRISMA-for-Abstracts content items are present.

Item	Requirement	Reported?	Location
1	Identify the report as a systematic review	☑	Title
2	State the main objective	☑	Abstract — Objective
3	Describe eligibility criteria	☑	Abstract — Methods (placebo-controlled COPD biologic RCTs)
4	Name information sources and last search date	☑	Abstract — Methods (PubMed/MEDLINE, CENTRAL, Europe PMC, ClinicalTrials.gov, WHO ICTRP, Google Scholar; 12 July 2026)
5	State risk-of-bias method	☑	Abstract — Methods (Cochrane RoB 2)
6	State synthesis methods	☑	Abstract — Methods (netmeta, Bayesian sensitivities, P-score, GRADE-CINeMA)
7	Give included-study scale	☑	Abstract—Results (15 trials; 9 maintenance RCTs)
8	Present main outcome results with precision	☑	Abstract — Results (four RRs, 95% CIs and P-scores)
9	State evidence limitations	☑	Abstract — Conclusions (star geometry; transitivity; eosinophil mixing)
10	Give a general interpretation	☑	Abstract — Conclusions
11	State review funding	☑	Abstract — Funding (none)
12	Give registration	☑	Abstract — Methods (PROSPERO CRD420261448694)

The operational inclusion/exclusion rubric (codes I1-I7/E1-E7), with worked application notes, was deposited at https://doi.org/10.5281/zenodo.21979837.

## References

[1] Adeloye D, Song P, Zhu Y, Campbell H, Sheikh A, Rudan I (2022). Global, regional, and national prevalence of, and risk factors for, chronic obstructive pulmonary disease (COPD) in 2019: a systematic review and modelling analysis. Lancet Respir Med.

[2] Soriano JB, Abajobir AA, Abate KH (2017). Global, regional, and national deaths, prevalence, disability-adjusted life years, and years lived with disability for chronic obstructive pulmonary disease and asthma, 1990-2015: a systematic analysis for the Global Burden of Disease Study 2015. Lancet Respir Med.

[3] Rabe KF, Rennard S, Martinez FJ (2023). Targeting type 2 inflammation and epithelial alarmins in chronic obstructive pulmonary disease: a biologics outlook. Am J Respir Crit Care Med.

[4] Fieldes M, Bourguignon C, Assou S (2021). Targeted therapy in eosinophilic chronic obstructive pulmonary disease. ERJ Open Res.

[5] Agustí A, Celli BR, Criner GJ (2023). Global Initiative for Chronic Obstructive Lung Disease 2023 report: GOLD executive summary. Eur Respir J.

[6] Donaldson GC, Seemungal TA, Bhowmik A, Wedzicha JA (2002). Relationship between exacerbation frequency and lung function decline in chronic obstructive pulmonary disease. Thorax.

[7] Suissa S, Dell'Aniello S, Ernst P (2012). Long-term natural history of chronic obstructive pulmonary disease: severe exacerbations and mortality. Thorax.

[8] McCann MR, Kosloski MP, Xu C, Davis JD, Kamal MA (2024). Dupilumab: mechanism of action, clinical, and translational science. Clin Transl Sci.

[9] Hillas G, Fouka E, Papaioannou AI (2020). Antibodies targeting the interleukin-5 signaling pathway used as add-on therapy for patients with severe eosinophilic asthma: a review of the mechanism of action, efficacy, and safety of the subcutaneously administered agents, mepolizumab and benralizumab. Expert Rev Respir Med.

[10] Varricchi G, Poto R (2024). Towards precision medicine in COPD: targeting type 2 cytokines and alarmins. Eur J Intern Med.

[11] Pascoe S, Locantore N, Dransfield MT, Barnes NC, Pavord ID (2015). Blood eosinophil counts, exacerbations, and response to the addition of inhaled fluticasone furoate to vilanterol in patients with chronic obstructive pulmonary disease: a secondary analysis of data from two parallel randomised controlled trials. Lancet Respir Med.

[12] Harries TH, Rowland V, Corrigan CJ (2020). Blood eosinophil count, a marker of inhaled corticosteroid effectiveness in preventing COPD exacerbations in post-hoc RCT and observational studies: systematic review and meta-analysis. Respir Res.

[13] Cazzola M, Bajpai J, Calzetta L, Matera MG, Rogliani P (2026). GOLD 2026: transforming COPD management with early intervention, multi-dimensional assessment, and personalized care. Drugs.

[14] (2026). Dupilumab (Dupixent) for COPD. Med Lett Drugs Ther.

[15] Menzies-Gow A, Corren J, Bourdin A (2021). Tezepelumab in adults and adolescents with severe, uncontrolled asthma. N Engl J Med.

[16] Pitre T, Lupas D, Mah J, Stanbrook M, Blazer A, Zeraatkar D, Ho T (2025). Biologic therapies for chronic obstructive pulmonary disease: a systematic review and network meta-analysis of randomized controlled trials. COPD.

[17] Sciurba FC, Criner GJ, Christenson SA (2025). Mepolizumab to prevent exacerbations of COPD with an eosinophilic phenotype. N Engl J Med.

[18] Papi A, Singh D, Virchow JC (2026). Safety and efficacy of astegolimab for COPD with frequent exacerbations regardless of baseline blood eosinophil counts (ALIENTO and ARNASA): randomised, double-blind, placebo-controlled, phase 2b and 3 trials. Lancet.

[19] Singh D, Guller P, Reid F (2025). A phase 2a trial of the IL-33 monoclonal antibody tozorakimab in patients with COPD: FRONTIER-4. Eur Respir J.

[20] Page MJ, McKenzie JE, Bossuyt PM (2021). The PRISMA 2020 statement: an updated guideline for reporting systematic reviews. BMJ.

[21] Hutton B, Salanti G, Caldwell DM (2015). The PRISMA extension statement for reporting of systematic reviews incorporating network meta-analyses of health care interventions: checklist and explanations. Ann Intern Med.

[22] Rethlefsen ML, Kirtley S, Waffenschmidt S, Ayala AP, Moher D, Page MJ, Koffel JB (2021). PRISMA-S: an extension to the PRISMA statement for reporting literature searches in systematic reviews. Syst Rev.

[23] Sterne JA, Savović J, Page MJ (2019). RoB 2: a revised tool for assessing risk of bias in randomised trials. BMJ.

[24] McGuinness LA, Higgins JP (2021). Risk-of-bias VISualization (robvis): an R package and Shiny web app for visualizing risk-of-bias assessments. Res Synth Methods.

[25] Criner GJ, Celli BR, Brightling CE (2019). Benralizumab for the prevention of COPD exacerbations. N Engl J Med.

[26] Singh D, Brightling CE, Rabe KF (2025). Efficacy and safety of tezepelumab versus placebo in adults with moderate to very severe COPD (COURSE): a randomised, placebo-controlled, phase 2a trial. Lancet Respir Med.

[27] Flynn CA, McAuley HJ, Elneima O (2025). Mepolizumab for COPD with eosinophilic phenotype following hospitalization. NEJM Evid.

[28] Rücker G (2012). Network meta-analysis, electrical networks and graph theory. Res Synth Methods.

[29] Balduzzi S, Rücker G, Nikolakopoulou A (2023). netmeta: an R package for network meta-analysis using frequentist methods. J Stat Softw.

[30] Rücker G, Schwarzer G (2015). Ranking treatments in frequentist network meta-analysis works without resampling methods. BMC Med Res Methodol.

[31] Phillippo DM (2026). multinma: Bayesian network meta-analysis of individual and aggregate data. R package version 0.8.1. https://zenodo.org/records/19660088.

[32] van Valkenhoef G, Kuiper J (2026). gemtc: network meta-analysis using Bayesian methods. https://cran.r-project.org/web/packages/gemtc/index.html.

[33] Nikolakopoulou A, Higgins JP, Papakonstantinou T, Chaimani A, Del Giovane C, Egger M, Salanti G (2020). CINeMA: an approach for assessing confidence in the results of a network meta-analysis. PLoS Med.

[34] Bhatt SP, Rabe KF, Hanania NA (2023). Dupilumab for COPD with type 2 inflammation indicated by eosinophil counts. N Engl J Med.

[35] Bhatt SP, Rabe KF, Hanania NA (2024). Dupilumab for COPD with type 2 inflammation indicated by eosinophil counts. N Engl J Med.

[36] Pavord ID, Chanez P, Criner GJ (2017). Mepolizumab for eosinophilic chronic obstructive pulmonary disease. N Engl J Med.

[37] Rabe KF, Celli BR, Wechsler ME (2021). Safety and efficacy of itepekimab in patients with moderate-to-severe COPD: a genetic association study and randomised, double-blind, phase 2a trial. Lancet Respir Med.

[38] Yousuf AJ, Samani NJ, Brightling CE (2022). Astegolimab, an anti-ST2, in chronic obstructive pulmonary disease (COPD-ST2OP): a phase 2a, placebo-controlled trial. Lancet Respir Med.

[39] Brightling CE, Bleecker ER, Panettieri RA Jr (2014). Benralizumab for chronic obstructive pulmonary disease and sputum eosinophilia: a randomised, double-blind, placebo-controlled, phase 2a study. Lancet Respir Med.

[40] Salanti G, Ades AE, Ioannidis JP (2011). Graphical methods and numerical summaries for presenting results from multiple-treatment meta-analysis: an overview and tutorial. J Clin Epidemiol.

[41] Wedzicha JA, Agustí À, Brightling CE (2026). Astegolimab for COPD with frequent exacerbations: pooled analysis of the ALIENTO and ARNASA trials. Am J Respir Crit Care Med.

[42] Li S, Yi B, Wang H, Xu X, Yu L (2025). Efficacy and safety of biologics targeting type 2 inflammation in COPD: a systematic review and network meta-analysis. Int J Chron Obstruct Pulmon Dis.

[43] Wang Q, Ma Q (2026). Number needed to treat with biologics in type-2 inflammation COPD: a systematic review and meta-analysis. COPD.

[44] García Morales OM, Díaz L, Auladell-Rispau A, Poloni D, Torres-López LA, Cuchi GU, Rojas-Reyes MX (2026). Biological therapy for chronic obstructive pulmonary disease (COPD): effectiveness and safety. Respir Med.

[45] Matera MG, Calzetta L, Rogliani P, Cazzola M (2025). Multi-criteria decision analysis of biologics in chronic obstructive pulmonary disease. Int J Chron Obstruct Pulmon Dis.

[46] Xue B, Xiao Q, Huang Y, Wang M (2025). Dupilumab for the treatment of COPD: protocol for a systematic review and meta-analysis. Front Med (Lausanne).

